# The roles of HMGB1‐produced DNA gaps in DNA protection and aging biomarker reversal

**DOI:** 10.1096/fba.2021-00131

**Published:** 2022-03-28

**Authors:** Sakawdaurn Yasom, Papitchaya Watcharanurak, Narumol Bhummaphan, Jirapan Thongsroy, Charoenchai Puttipanyalears, Sirapat Settayanon, Kanwalat Chalertpet, Wilunplus Khumsri, Aphisek Kongkaew, Maturada Patchsung, Chutha Siriwattanakankul, Monnat Pongpanich, Piyapat Pin‐on, Depicha Jindatip, Rujira Wanotayan, Mingkwan Odton, Suangsuda Supasai, Thura Tun Oo, Busarin Arunsak, Wasana Pratchayasakul, Nipon Chattipakorn, Siriporn Chattipakorn, Apiwat Mutirangura

**Affiliations:** ^1^ Center of Excellence in Molecular Genetics of Cancer and Human Disease, Department of Anatomy, Faculty of Medicine Chulalongkorn University Bangkok Thailand; ^2^ Interdisciplinary Program of Biomedical Sciences, Graduate School Chulalongkorn University Bangkok Thailand; ^3^ School of Medicine Walailak University Nakhon Si Thammarat Thailand; ^4^ Research Administration Section, Faculty of Medicine Chiang Mai University Chiang Mai Thailand; ^5^ Department of Mathematics and Computer Science, Faculty of Science Chulalongkorn University Bangkok Thailand; ^6^ Omics Sciences and Bioinformatics Center, Faculty of Science Chulalongkorn University Bangkok Thailand; ^7^ Department of Radiological Technology, Faculty of Medical Technology Mahidol University Nakhon Pathom Thailand; ^8^ Department of Molecular Tropical Medicine and Genetics, Faculty of Tropical Medicine Mahidol University Bangkok Thailand; ^9^ Neurophysiology Unit, Cardiac Electrophysiology Research and Training Center, Faculty of Medicine Chiang Mai University Chiang Mai Thailand; ^10^ Center of Excellence in Cardiac Electrophysiology Research Chiang Mai University Chiang Mai Thailand; ^11^ Cardiac Electrophysiology Unit, Department of Physiology, Faculty of Medicine Chiang Mai University Chiang Mai Thailand

**Keywords:** aging, DNA damage, DNA gap, rejuvenation, RIND‐EDSB, senescence, youth‐DNA‐GAP

## Abstract

The endogenous DNA damage triggering an aging progression in the elderly is prevented in the youth, probably by naturally occurring DNA gaps. Decreased DNA gaps are found during chronological aging in yeast. So we named the gaps “Youth‐DNA‐GAPs.” The gaps are hidden by histone deacetylation to prevent DNA break response and were also reduced in cells lacking either the high‐mobility group box (HMGB) or the NAD‐dependent histone deacetylase, SIR2. A reduction in DNA gaps results in shearing DNA strands and decreasing cell viability. Here, we show the roles of DNA gaps in genomic stability and aging prevention in mammals. The number of Youth‐DNA‐GAPs were low in senescent cells, two aging rat models, and the elderly. Box A domain of HMGB1 acts as molecular scissors in producing DNA gaps. Increased gaps consolidated DNA durability, leading to DNA protection and improved aging features in senescent cells and two aging rat models similar to those of young organisms. Like the naturally occurring Youth‐DNA‐GAPs, Box A‐produced DNA gaps avoided DNA double‐strand break response by histone deacetylation and SIRT1, a Sir2 homolog. In conclusion, Youth‐DNA‐GAPs are a biomarker determining the DNA aging stage (young/old). Box A‐produced DNA gaps ultimately reverse aging features. Therefore, DNA gap formation is a potential strategy to monitor and treat aging‐associated diseases.

**Abbreviations**
8‐OHdG: 8‐hydroxy‐2′‐deoxyguanosine
ALP: alkaline phosphatase
ALT: alanine aminotransferase
AP: Apurinic/apyrimidinic
AST: aspartate aminotransferase
DDR: DNA damage response
D‐gal: D‐galactose
DIP: DNA immunoprecipitation
DI‐PLA: DNA damage in situ ligation detected by a proximity ligation assay
DMEM: Dulbecco's Modified Eagle's Medium
FBS: fetal bovine serum
FLAG: anti‐DDDDK tag or anti‐DYKDDDDK tag
H&E: hematoxylin & eosin
H2O2: hydrogen peroxide
HMGB: high‐mobility group box
HMWDNA: High‐molecular‐weight DNA
HRP: horseradish peroxidase
IHC: immunohistochemical staining
IRSs: interspersed repetitive sequences
LM‐PCR: ligation‐mediated PCR
m: month
MMS: methyl methanesulfonate
MWM: Morris water maze
n: number
NCDs: noncommunicable diseases
NSS: normal saline solution
PBS: phosphate‐buffered saline
PC: scrambled sequence plasmid control
PI: propidium iodine
RIND‐EDSB: replication‐independent EDSBs
SA‐β‐gal: senescence‐associated β‐galactosidase
SSBs: DNA single‐strand breaks
TP: total protein
TSA: Trichostatin A
Youth‐DNA‐GAPs: youth‐associated genome‐stabilizing DNA gaps
γ‐H2A.X: phosphorylated histone variant H2A.X

## 1 INTRODUCTION

**FIGURE 1:**
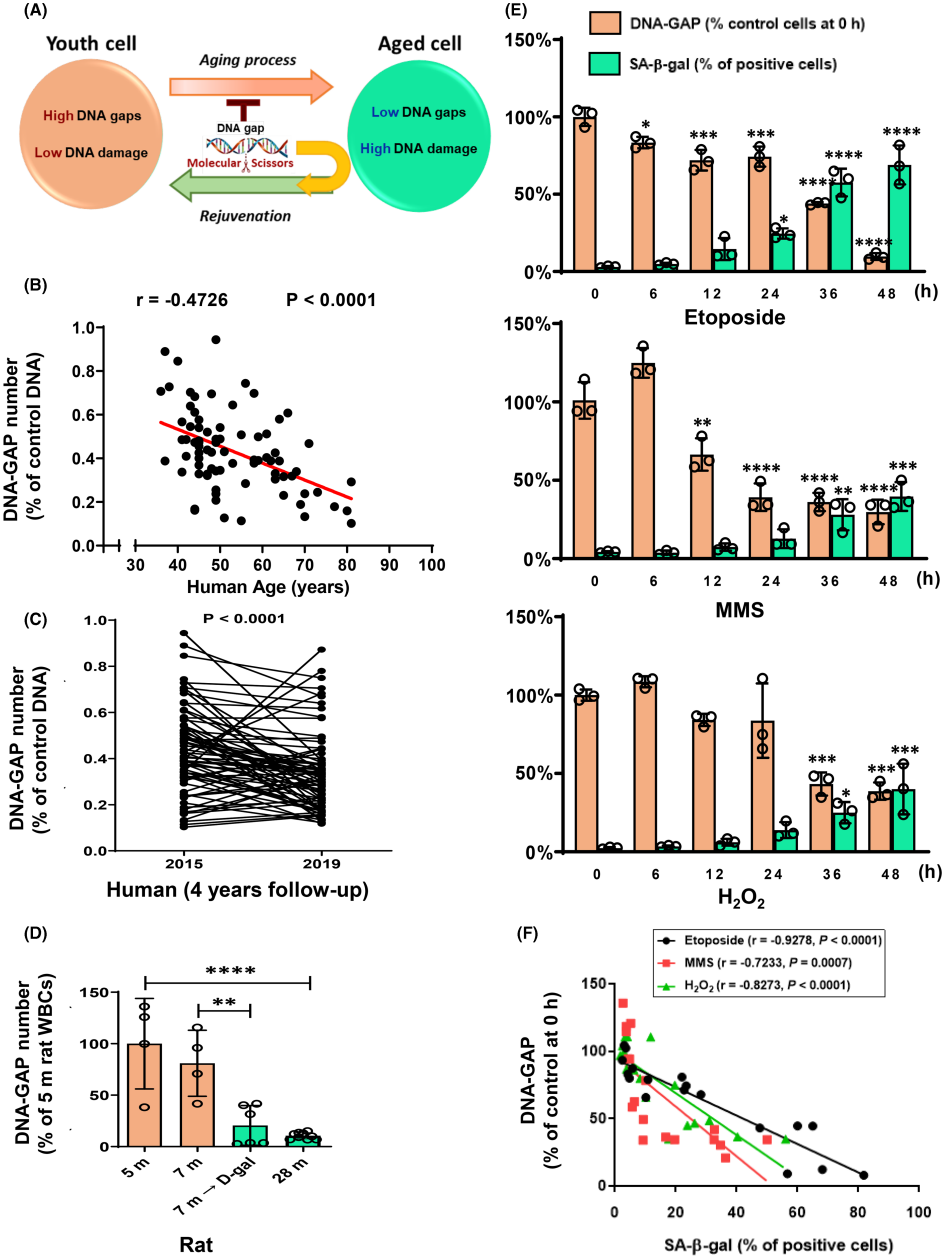
Reduction in Youth‐DNA‐GAPs during chronological aging in mammals. We measured Youth‐DNA‐GAPs (DNA‐GAP) in healthy aging elderly, naturally aging rats, D‐galactose (D‐gal)‐induced aging rats, and different chemicals‐caused senescent cells. (A) A diagram representing the homeostasis of DNA gaps and DNA damage. Molecular scissors represent DNA gap‐forming proteins. (B) Correlation between the number of DNA gaps and age (*n* = 80). Pearson's correlation coefficient (R) with the P‐value is indicated. (C) Youth‐DNA‐GAP levels at the 4‐year follow‐up (2015–2019) in the same person (*n* = 76). P‐value is indicated (paired *t*‐test). (D) The numbers of DNA gaps in 5‐month‐old (*n* = 4), 7‐month‐old (*n* = 4), 7‐month‐old D‐gal induced (*n* = 6), 30‐month‐old (*n* = 14) rats. (E) The relative percentage of DNA gaps and SA‐β‐gal‐positive cells in the cells exposed to 2.5 μM etoposide, 50 μM MMS, and 100 μM H_2_O_2_ at different time points from 0 to 48 h (each data point is the mean of six experiments under intra‐assay conditions). (F) Correlation between the number of DNA gaps and SA‐β‐gal‐positive cells. Pearson's correlation coefficient (r) with the P‐value is indicated. Data represented in (B) and (C) are the percentage of the number of DNA gaps of control DNA. The number of DNA gaps in senescent cells in 5‐month‐old rats (D) and at 0 h (E) was normalized to 100%. All experimental data were independent biological samples. (D) and (E) data represent mean ± SEM. *p ≤ 0.05, **p ≤ 0.01, ***p ≤ 0.001, ****p ≤ 0.0001 from one‐way ANOVA followed by post hoc analysis

Elderly adults have a higher accumulation of damaged DNA than young adults (Figure 1A).^1^, ^2^ The DNA damage in aging cells causes impaired biological function, frailty, disability, and health decline.^1^ DNA destruction drives the aging process via the DNA damage response (DDR). The persistent DDR activity can promote cellular senescence, a stable arrest of the cell cycle accompanied by stereotyped phenotypic changes, and consequently aging‐related characteristics from the molecular to the clinical levels, as well as an increased risk of noncommunicable diseases (NCDs).^1^, ^3^, ^4^, ^5^, ^6^, ^7^, ^8^, ^9^, ^10^, ^11^, ^12^ However, the underlying mechanism of DNA damage in the elderly is unknown.

One of the cellular mechanisms to maintain DNA integrity is the formation of DNA gaps. Our genome possesses naturally occurring DNA gaps that maintain genomic integrity and these gaps decrease in old yeast.^13^, ^14^ Hereafter we referred to the DNA gaps as youth‐associated genome‐stabilizing DNA gaps (Youth‐DNA‐GAPs).^15^ Youth‐DNA‐GAP role is similar to the gaps left between successive rails on a railway track, relieving torsion force to prevent the track damage. Previously, we proved that reducing the DNA gap led to DNA shearing.^14^ However, the DNA appearance of the gap is the same as the DNA double strand break (DSB). So to prevent DNA gaps causing genomic instability, cells must fix the DNA ends of a gap from separation, that is, maintain their adjacency and hide the DNA gap from DSB response. During gaps of railway track construction, the engineer uses a joint railroad bar to fix the gap to prevent railway tracks misplaced. We demonstrated that Youth‐DNA‐GAP ends are retained within deacetylated histone, and the heterochromatin formation hides Youth‐DNA‐GAP ends from DSB response.^16^

The biology of Youth‐DNA‐GAP is similar to topoisomerase‐creating DNA gap.^17^ Topoisomerase II involves the relaxation of positive supercoiling of DNA and limits DNA torsional energy via topoisomerase‐creating DNA gap.^18^ As a result, relieving the torsional force through DNA gap activity can prevent DNA structure from damage. Treating cells with topoisomerase II inhibitor increases DNA damage.^19^ In 2008, we reported another type of naturally occurring DNA gaps.^13^ The gaps are a distinctive type of endogenous DSBs (EDSBs), previously named physiological replication‐independent EDSBs (RIND‐EDSBs).^14^ They possess beneficial and essential roles in DNA modifications.^13^, ^17^ They are evolutionarily conserved, produced and maintained by cellular proteins, stabilize DNA, and are present in all eukaryotic cells and cell cycle phases.^13^, ^14^, ^17^ In yeast, aging DNA has a lower level of naturally occurring DNA gaps than youthful DNA (Figure 1A).^14^ Moreover, a reduction in DNA gaps induces spontaneous DNA shearing and cell mortality.^14^ Hereafter to avoid confusion between pathologic DSBs and physiologic DSBs we referred to the physiologic RIND‐EDSBs as DNA gaps or in this case, Youth‐DNA‐GAPs.^15^ DNA gaps are the same as pathological DSBs that if they are present in the G1 phase before DNA replication, they must be repaired prior to DNA replication fork pass through the DNA modifications. However, unlike pathological DSBs that are predominantly repaired by Ku‐mediated nonhomologous end joining, the physiological RIND‐EDSBs are repaired by a more precise ATM‐dependent pathway.^16^ Here, in this article, we will identify the Youth‐DNA‐GAP producer and prove if producing Youth‐DNA‐GAP can improve DNA durability and consequently revitalize age of DNA.

In addition to chronological aging in yeast, decreased Youth‐DNA‐GAPs are found in cells lacking either high‐mobility group box (HMGB) or NAD‐dependent histone deacetylase SIR2.^14^, ^17^ We speculated that Box A of HMGB1 is a key protein that can form Youth‐DNA‐GAPs due to the following possibilities. As mentioned above, the level of Youth‐DNA‐GAPs is low in cells lacking one intact HMGB (i.e., its mutants).^17^ Moreover, based on the DSB structure, the proteins forming Youth‐DNA‐GAPs must be a nuclease. HMGB1 possesses deoxyribose phosphate lyase activity that can cleave DNA.^20^ HMGB1 can bend DNA and protect DNA from denaturation.^21^ Without a DNA gap, DNA bending usually reduces the strength of hydrogen bonds of DNA. So HMGB1 may form DNA gaps so that DNA can be bent and stabilized simultaneously. The HMGB1 gene contains two DNA‐binding domains (Box A and Box B) and an acidic tail (Box C). Phe37 in rats (Phe38 in humans) in the Box A domain plays a vital role in kinking DNA,^22^ suggesting that this amino acid may play a critical role in DNA gap formation. Thus, we will investigate whether the formation of DNA gaps occurs by human Box A and is dependent on its Phe38.

Chromatin condenses to mask Youth‐DNA‐GAPs from the DSB response—phosphorylated histone variant H2A.X (γ‐H2A.X).^16^ Interestingly, we recently demonstrated a decrease in Youth‐DNA‐GAPs in cells without NAD‐dependent histone deacetylase SIR2.^17^ Moreover, HMGB1 Box A can bind to SIRT1, a human Sir2 homolog.^23^ In this regard, the functions of HMGB1 in producing DNA gaps may solely depend on Box A sequence as the molecular scissors and its function in binding to SIRT1 is to prevent the DSB response and repair.

Intracellular reduction of HMGB1 and HMGB1 release is extensively described in aging studies in vivo and in vitro.^24^, ^25^, ^26^, ^27^, ^28^, ^29^, ^30^, ^31^, ^32^, ^33^ A large number of studies evaluated the role of extracellular HMGB1 primarily associated with pathological aging process and inflammation.^28^, ^29^, ^30^, ^31^, ^32^, ^33^ In contrast, intranuclear HMGB1 plays a protective role in genomic stabilization. Yeast cells with an incomplete *HMGB1* homolog gene—its mutants—showed increases in DNA damage, pathological DSBs, and vulnerability to UV light.^14^, ^34^, ^35^ In mammals, nuclear HMGB1 prevents heart failure via DDR inhibition.^25^ Furthermore, a reduction in HMGB1 positively correlates with the accumulation of γH2A.X in the mouse brain.^27^ Thus, HMGB1 can prevent DNA damage and DDR, suggesting that loss of intranuclear HMGB1 in aging cells may cause DNA damage.

Based on this evidence, we hypothesized that HMGB1 Box A plays a role in Youth‐DNA‐GAP formation, and Box A‐produced DNA gaps protect DNA from being damaged. To prove these concepts, we first investigated changes in DNA gaps in experimental aging model organisms and clinical aging humans. Second, we introduced exogenous Box A into cells, senescence cells and two models of aging rats to test whether Box A can increase DNA gaps, decrease DNA damage and, in turn, decelerate the aging process.

## 2 MATERIAL AND METHODS

### 2.1 Participants

We recruited all subjects from the Tambon Health Promoting Hospital service, Nakhon Si Thammarat, Thailand, in 2015 and 2019. The participants' age was more than 35 years. Hemoglobin A1C levels were assessed to exclude individuals with diabetes mellitus. All subjects voluntarily participated in the study. The Ethics Clearance Committee on Human Rights Related to Research Involving Human Subjects at Walailak University, Nakhon Si Thammarat, Thailand, reviewed and approved the study. We carried out all methods involving human participants following the WHO guidelines and the Declaration of Helsinki. All of the participants provided written informed consent.

### 2.2 Cell culture

The cell lines used in this study, HEK293 ATCC® CRL‐1573 (human embryonic kidney cell line) and HK‐2 ATCC® CRl‐2190 (kidney proximal tubule epithelial cell line), were purchased from the American Type Culture Collection (ATCC). They were cultured in Dulbecco's modified Eagle's medium (DMEM) (Gibco) supplemented with 10% fetal bovine serum (FBS) (Gibco) and 1% antibiotic‐antimycotic (Gibco) in 25‐ and 75‐cm^3^ culture flasks and were maintained in a humidified atmosphere with 5% CO_2_ at 37°C.

### 2.3 Senescence induction

For senescence induction, we treated cells with 2.5 μM etoposide (Sigma‐Aldrich), 50 μM Methyl methanesulfonate (MMS) (Merck Millipore), and 100 μM hydrogen peroxide (H_2_O_2_) (Sigma‐Aldrich) for 0–48 h.

### 2.4 High‐molecular‐weight DNA (HMWDNA) preparation

To preserve the integrity of genomic DNA, HMWDNA was prepared. Cells (approximately 1 × 10^6^ cells) were collected as previously described.^13^ Briefly, cells were mixed and embedded in 70 μl of 1% low‐melting‐point agarose (MO BIO Laboratories), and cell‐containing gels (plugs) were lysed and digested in 400 μl lysis buffer (50 mM Tris pH 8.0, 20 mM EDTA, 1% sodium lauryl sarcosine, and 1 mg/ml proteinase K) and incubated overnight at 37°C. The next day, the digested plugs were washed with Tris‐EDTA (T10E2) buffer 6 times for 40 min. Cohesive end‐DNA was then polished using T4 DNA polymerase (New England Biolabs) and dNTP (New England Biolabs) addition.

### 2.5 HMWDNA preparation for DNA‐GAP PCR

Plugs were incubated at 17°C for 90 min and washed four times with T10E2 for 20 min. Ligation‐mediated PCR (LM‐PCR) linkers (5′‐AGGTAACGAGTCAGACCACCGATCGCTC‐GGAAGCTTACCT‐CGTGGACGT‐3′ and 5′‐ACGTCCACGAG‐3′) were prepared and ligated to the polished DNA in plugs using T4 DNA ligase (New England Biolabs), and plugs were incubated at room temperature for two nights. Then, HMWDNA was extracted from the plugs using a TIANgel Midi Purification Kit (Tiangen Biotech). HMWDNA from each plug was diluted to 20 ng/μl for DNA‐GAP PCR.

### 2.6 DNA‐GAP measurement

DNA‐GAP PCR or interspersed repetitive sequences (IRSs)‐EDSB‐LM‐PCR was prepared as previously reported.^13^ To determine EDSBs in cells, HMWDNA was obtained for DNA‐GAP PCR by using a QuanStudio™ 6 Flex Real‐Time PCR system (Thermo Fisher Scientific). The PCR components were 1x TaqMan™ Universal PCR Master Mix (Applied Biosystems); 0.5 U of HotStarTaq DNA polymerase (Qiagen, Hilden, Germany); 0.3 μM f probe homologous to the 3′‐linker sequence (6‐fam) ACGTCCACGAGGTAAGCTTCCGAGCGA (tamra) (phosphate); 0.5 μM of human interspersed repetitive sequences (IRSs) primer (LINE‐1) (5′‐CTCCCAGCGTGAGCGAC‐3′) for human subjects, (Alu) (5′‐ACTGCACTCCAGCCTGGGC‐3′) for in vitro experiments, or (B1) (5′‐AATCCGCCTGCCTCTGCCTCC‐3′) for rat subjects; 0.5 μM linker primer (5′‐AGGTAACGAGTCAGACCACCGA‐3′); and 40 ng of HMWDNA. Control DNA digested by EcoRV and AluI (Thermo Fisher Scientific) and ligated with linkers was used to generate a standard curve. The PCR cycle was set as follows: 1 cycle of 50°C for 2 min followed by 95°C for 10 min and 60 cycles of 95°C for 15 s along with 60°C for 2 min. For human subjects, the amount of DNA‐GAP PCR in each test was compared to that of the digested ligated control DNA and reported as the percentage of DNA‐GAP PCR amplicons of control DNA (%DNA‐GAP number of control DNA). For in vitro and animal studies, the amount of DNA‐GAPs was calculated from a standard curve of control DNA and reported as %DNA‐GAP PCR, which was 100 times the experimental group and divided by the control group (%DNA‐GAP number of control cells or subjects).

### 2.7 Senescence‐Associated β‐Galactosidase (SA‐β‐gal) assay

Cells were seeded in 24‐well plates at a density of approximately ×10^4^ cells/well before treatment with etoposide, MMS, and H_2_O_2_. Then, the cells were washed with 1× phosphate‐buffered saline (PBS), fixed, and stained using the SA‐β‐gal Staining Kit (Cell Signaling Technology) according to the manufacturer's instructions. The cells were incubated overnight at 37°C without CO_2_. Finally, cells were visualized and inspected for blue staining under a bright‐field microscope and imaged by fluorescence microscopy (Nikon Eclipse Ts2)(Nikon). We calculated the percentage of SAβ‐gal‐positive cells by counting the cells in five random fields.

### 2.8 Plasmid construction

We used full‐length human HMGB1, Box A, Box B, Box BC, scrambled sequence plasmid control (PC), and three Box A sequences with point mutations corresponding to the p.F38Y, p.F38W, p. F38G, and pcDNA™3.1(+)(Thermo Fisher Scientific) plasmids in this study. The mutant plasmids were constructed by GeneArt Gene Synthesis (Thermo Fisher Scientific). We transformed the plasmids into *Escherichia coli* (DH5α) (Invitrogen), specifically, NEB® 5‐alpha competent *E. coli* (New England BioLabs). For all plasmid selection, transformed cells were grown on LB agar with ampicillin. The selected colony was further cultured in LB broth with 100 μg/ml ampicillin and incubated on an incubator shaker at 37°C for 16 h. We extracted the plasmids using the GeneJET Plasmid Miniprep Kit (Thermo Fisher Scientific).

We also constructed a Box A‐GFP plasmid using the pLenti‐C‐mGFP‐P2A‐Puro vector (Origene Technologies) containing GFP with Box A of the HMGB1 sequence inserted. A scramble‐GFP plasmid provided by Origene Technologies, Inc. was utilized as a GFP transfection control. We transformed each plasmid into competent *E. coli* (DH5α) cells. For animal experiments, after selective bacterial culture, the plasmids were extracted and purified using a GeneJet Plasmid Maxiprep Kit (Thermo Fisher Scientific) according to the manufacturer's instructions. Sequence fidelity was confirmed by Sanger sequencing.

### 2.9 Plasmid transfection

HEK293 and HK‐2 cells (3 × 10^5^ cells/ml) were seeded into 6‐well plates containing growth medium (DMEM) for 24 h. Then, 2500 ng of each plasmid was transfected using Lipofectamine 3000 (Thermo Fisher Scientific) according to the manufacturer's instructions.

### 2.10 Flow cytometry for cell cycle analysis using propidium iodine (PI)

The pellets of transfected cells were collected into 1.5 ml Eppendorf tube. Add dropwise 500 μl cold 70% ethanol (Sigma Chemical) into the pellet while vortexing for fixation step. The samples were incubated at 4°C for 30 min. The pellets were washed twice with 500 μl of 1× PBS and centrifuged at 4°C, 500 g for 10 min. Then, the supernatants were discarded. The pellets were treated with 50 μl of 10 μg/ml ribonuclease (RNase) (Merck) and incubated at room temperature for 30 min. Add 200 μl of PI (ab14083, Abcam co., Ltd) into the samples and incubated at room temperature for 15 min in dark condition. Transfer the sample into 5 ml round bottom polystyrene tube for flow cytometry (FACs tube, Falcon® Corning). The fluorescent signal was detected through the fluorescent PE channel by DxFLEX Flow cytometer (Beckman Coulter, Inc.). The percentages of transfected cell population in the phase of cell cycle were calculated using CytExpert Software for DxFLEX version 2.0.0.283 (Beckman Coulter, Inc.).

### 2.11 Polypeptide preparation

We purchased HMGB1 protein and human Box A, Box A, p.F38Y, p.F38W, p. F38G, and Box B polypeptides from TECAN (Tecan, Maennedorf, Switzerland). For DNA preparation for next‐generation sequencing (NGS), HMGB1 was produced from HMGB1 cDNA (NM_001313893.1) in the pRSET A vector (Thermo Fisher Scientific). We also synthesized the HMGB1 protein with a pRSETA‐HMGB1 plasmid constructed by GeneArt Gene Synthesis (Thermo Fisher Scientific) into BL21(DE3) pLysS competent cells (Promega). The transformed cells were cultured in 2YT medium when the OD600 reached 0.8, and 1 mM IPTG (Sigma‐Aldrich) was used to induce overexpression. Cells were further cultured at 20°C for 20 h in a 250‐rpm shaking incubator and collected by 12 000 rpm centrifugation. We performed HMGB1 protein purification by using a HISTrap™HP column (GE Healthcare).

### 2.12 In vitro DNA digestion

We tested various concentrations of each polypeptide for treating HMWDNA with 1x CutSmart® buffer (New England Biolabs) in a total volume of 400 μl at 37°C for 12 h. Then, we washed the DNA with T10E2 for 20 min four times. HMWDNA was subjected to 1% agarose gel electrophoresis stained with SYBR® Green II Nucleic Acid Gel stain (Lonza, Basel, Switzerland). DNA fragmentation was observed as smear bands on gel visualized by an Azure c150 Gel Imaging System (Azure Biosystems). To compare DSB generation quantitatively, we incubated each of these proteins with each plug. The treated HMWDNA was prepared and assessed by DNA‐GAP PCR. We performed a statistical analysis using a paired‐sample *t*‐test.

### 2.13 Establishment of stable knockdown of HMGB1 and SIRT1

We obtained three shRNA constructs in a lentiviral GFP vector targeting HMGB1 (HMGB1‐Human, four unique 29‐mer shRNAs; Cat. No. TL316576), SIRT1 (SIRT1‐Human, four unique 29 mer shRNAs; Cat. No. TL309433), and a scrambled negative control (noneffective 29‐mer shRNA cassette in pGFP‐C‐shLenti vector; cat. no. TR30021) from Origene Technologies Inc. (Rockville, MD, USA). We produced lentiviral particles by transfecting the shRNA constructs into HEK293T cells with lentiviral packaging vectors (Lentivpak packaging kit; cat. no. TR30037; OriGene) using Turbofectin transfection reagent (Origene) according to the manufacturer's instructions. After transfection for 48 h, the virus‐containing supernatants were collected and filtered through a 0.45‐μm‐pore‐size filter. Before transduction, HEK293 cells were seeded at a density of 5 × 10^4^ cells/well in a 24‐well plate for 24 h. HEK293 cells were then transduced with lentiviral particles at an MOI (multiplicity of infection) of 5 in the presence of 8 μg/μl polybrene (Sigma‐Aldrich). After 24 h, we removed the culture medium and added a fresh medium to the cells. Seventy‐two hours after transduction, we added 1 μg/ml puromycin to the medium for stable cell line selection. The knockdown efficacy was assessed by real‐time PCR and western blot analysis.

To observe the mRNA expression of *HMGB1* and *SIRT1*, total cellular RNA was isolated by TRIzol reagent (Invitrogen) according to the manufacturer's protocol. ComplementaryDNA was synthesized using the RevertAidTM first‐strand cDNA synthesis kit (Thermo Fisher Scientific). Oligonucleotide primer sequences were as follows: HMGB1 forward: 5′‐ATATGGCAAAAGCGGACAAG‐3′, HMGB1 reverse: 5′‐GCAACATCACCAATG‐GACAG‐3′, SIRT1 forward: 5′‐GGTACCGAGATAACCTCCTG‐3′, SIRT1 reverse: 5′‐CATGTGAGGCTCTATCCTCC‐3′, GAPDH forward: 5′‐TGGAAGGACTCATGACCACAG‐3′, and GAPDH reverse: 5′‐TCCAGCTCAGGGATGACCTT‐3′. The PCR conditions were 40 cycles of 95°C 15 s, 57°C 30 s, and 72°C 45 s. Gene expression was quantified and normalized to that of the *GAPDH* housekeeping gene.

To determine the protein expression of HMGB1‐ and SIRT1‐knockdown cells, western blot analysis was performed. The antibodies used were as follows: HMGB1 (ab18256)(Abcam, Cambridge, UK), SIRT1 (ab110304, Abcam), goat anti‐rabbit IgG‐HRP (7074 s) (Cell Signaling Technology), and goat anti‐mouse IgG‐HRP (7076 s, Cell Signaling).

### 2.14 Trichostatin A (TSA) treatment

After plasmid transfection, cells were treated with 200 ng/ml TSA (Sigma‐Aldrich) for 6 h.

### 2.15 Nuclear extract

Transfected cells were collected and centrifuged for 10 min at 3000 rpm. We removed the supernatant, resuspended the cell pellets with 500 μl of hypotonic buffer solution (20 mM Tris–HCl, pH 7.4, 10 mM NaCl, 3 mM MgCl_2_) and incubated them on ice for 15 min. The cells were lysed by adding 15 μl of 10% NP40 in PBS and vortexing for 10 s. The nuclear pellets were separated by centrifugation for 10 min at 3000 rpm and resuspended in 50 μl of PBS. Five microliters of nuclear fraction were smeared in 96‐well plates and dried at room temperature for 20 min. Next, 100 μl of 3.7% formaldehyde in PBS was applied to the sample for 15 min at room temperature to fix the nucleus to assess morphology. The samples were washed five times with 100 μl of PBS to remove the fixative agent.

### 2.16 Colocalization assay

We performed colocalization experiments using a proximity ligation assay (PLA) and a DNA damage in situ ligation detected by a proximity ligation assay (DI‐PLA) following the manufacturer's protocol (Duolink® PLA detection reagent orange, DUO92007, Sigma‐Aldrich).^36^ Briefly, the samples were permeabilized with 2% Triton X solution for 10 min at room temperature. The samples were washed twice with PBS. One drop of Duolink® Blocking Solution was added, and the sample was incubated for 60 min at 37°C. The nucleus was washed five times with PBS. Then, 100 μl of the blunting solution (1 mM dNTPs, 10 μl NEB buffer 2.1 (New England Biolabs), and 1 μl T4 DNA polymerase (5 U/μl, Thermo Fisher Scientific) was applied to the samples, and the sample was incubated for 60 min at 16°C. The DI‐PLA linker with biotin, tagged was prepared in 50 μl of ligation solution (5 μl T4 ligase buffer 10×, 1.5 μl T4 DNA ligase (5 U/μls, Thermo Fisher Scientific), 0.5 μM DI‐PLA linker, 0.2 mg/ml BSA. We added 50 μl of ligation solution to the samples and incubated them overnight at 37°C. Anti‐DDDDK tag (FLAG) mouse monoclonal antibody (ab125243)(Abcam), anti‐DDDDK tag (FLAG) rabbit polyclonal antibody (ab1162)(Abcam), anti‐SIRT1 mouse monoclonal antibody (ab110304)(Abcam), anti‐biotin rabbit polyclonal antibody (ab53494)(Abcam), and anti‐γH2A.X rabbit monoclonal antibody (9817 s)(Cell Signaling) antibodies were used, and goat anti‐mouse‐Cy3 (ab97035)(Abcam) were diluted in a 1:1000 ratio with reaction buffer (1% FBS and 0.5% Tween 20 in PBS) and incubated for 3 h at 37°C. We observed protein expression in transfected cell lines with the combination of anti‐FLAG and goat anti‐mouse Cy3. For DI‐PLA, the anti‐biotin rabbit polyclonal antibody was combined with the anti‐DDDDK tag or anti‐SIRT1 mouse monoclonal antibody. Anti‐γH2A.X rabbit monoclonal antibody combined with anti‐DDDDK tag mouse monoclonal antibody and anti‐SIRT1 mouse monoclonal antibody combined with anti‐DDDDK tag rabbit polyclonal antibody were prepared for the PLA reaction. Next, we incubated the samples with Duolink® PLA plus and minus probe diluted 1:50 with Duolink® antibody diluent for 2 h at 37°C. The ligation reaction (1 μl Duolink® ligase, 8 μl Duolink® ligation buffer, 32 μl dH_2_O) was prepared and added to the samples (60 min, 37°C). The amplification solution (0.5 μl Duolink® polymerase, 8 μl Duolink® polymerase buffer, 32 μl dH_2_O) was applied and incubated for 2 h at 37°C. The samples were washed five times at room temperature. Finally, Hoechst nuclear stain (1 μg/μl) was added and incubated for 10 min at 37°C. We identified the positive fluorescent spots from the nucleus with a confocal microscope (20× and 40×).

### 2.17 Calculation of the positive fluorescent signal

We evaluated the positive fluorescent signals as red spots in the nuclei of transfected cells. The positive signals were enumerated in six fields (3 columns × 2 rows) of a 40× objective lens at the center of the well with specific exposure times, including Hoechst (20 ms) and positive spot (800 ms). The positive cells with different numbers of positive signals from 1 to 9 spots were counted and classified in the spot distribution. We calculated the percentage of positive cells by the number of positive nuclei divided by the total number of nuclei. All nuclei with positive spots were counted, and the fluorescence intensity was observed by CellSens® imaging software (Olympus® Co., Ltd.). We performed the experiments in triplicate. The fluorescence intensity of positive signals in the plasmid control‐transfected cell line was applied as an interassay variation adjustment.

### 2.18 Determination of endogenous DNA damage

Cells were harvested by trypsinization and processed for DNA extraction using DNAzol® (Thermo Fisher Scientific) according to the manufacturer's instructions. Finally, DNA was solubilized using 8 mM NaOH.

### 2.19 8‐hydroxy‐2′‐deoxyguanosine (8‐OHdG) measurement

We measured 8‐OHdG levels in DNA using an OxiSelect™ Oxidative DNA Damage ELISA Kit (8‐OHG Quantitation) (Cell Biolabs). Briefly, the unknown 8‐OHdG DNA samples and the 8‐OHdG standard were first added to a microplate coated with BSA‐conjugated 8‐OHdG. Second, an anti‐8‐OHdG antibody monoclonal antibody was added, followed by an HRP‐conjugated secondary antibody. After the incubation process, the reaction was observed by absorbance measurement using a microplate reader (Bio‐Rad) at 450 nm as a primary wavelength and 620 nm as a reference wavelength. 8‐OHdG levels in unknown DNA samples were determined by comparison with a predetermined 8‐OHdG standard curve.

### 2.20 Apurinic/apyrimidinic (AP) sites measurement

We determined the AP site level by an OxiSelect™ Oxidative DNA Damage Quantitation Kit (AP site) (Cell Biolabs). AP site levels were detected by a aldehyde reactive probe that reacted specifically with an aldehyde group on the open‐ring form of the AP site. As a result, they were tagged with biotin and then detected with a streptavidin‐enzyme conjugate. After incubation, we washed the incubated complexes in microwells and added substrate solution to measure the enzymatic reaction. Then, the reaction was quenched by adding a stop solution. Finally, we read the absorbance at 450 and 620 nm by using a microplate reader.

### 2.21 Cell proliferation MTT assay

To investigate cell proliferation, HMGB1, box A, and mutant Box A plasmids were transfected for 48 h, harvested, seeded into a 96‐well plate (5 × 10^3^ cells/well in 100 μl DMEM) and incubated at 37°C for 24 h. The next day, cell proliferation was assessed for four consecutive days using MTT reagent (5 mg/ml) (Sigma‐Aldrich). The absorbance was measured at 570 nm using a microplate reader.

### 2.22 X‐ray exposure and counting of γH2A.X foci

To observe whether Box A is more resistant to ionizing radiation and participates in DNA double‐strand break‐induced damage response prevention, Box A, Box A mutant, HMGB1, and PC‐transfected HEK293 and HK2 cells were irradiated by x‐ray exposure, and γH2A.X foci were then counted. After transfection for 48 h, cells were exposed to x‐rays. X‐ray irradiation was performed at room temperature using a 6 MV Clinac®iX system linear accelerator (Varian Medical Systems) at a dose rate of 2 Gy/min. Then, we collected cells by trypsinization and washed them three times with PBS. The cells were processed for immunofluorescent staining.

We fixed cells with 4% paraformaldehyde (Sigma‐Aldrich) for 15 min at room temperature and washed them three times for 2 min with PBS. One hundred microliters of PBS was added to the cells and resuspended. Five microliters of fixed cells was smeared into a 96‐well plate. After drying, permeabilization was performed using 0.5% Triton X‐100 in PBS (PBST) at room temperature for 5 min, followed by washing with PBS. Cells were then blocked with 1% FBS in PBS for an hour at room temperature, and a 1:100 dilution of anti‐HA tag antibody (ab18181) (Abcam) was added to the cells. Cells were incubated at 4°C overnight. Cells were washed three times with PBST for 15 min before the addition of a 1:500 dilution of preadsorbed goat anti‐mouse IgG H&L (Cy3®) (ab97035) (Abcam) and incubated at room temperature for an hour in the dark. Then, the cells were immediately washed three times with PBST and blocked with 1% FBS in PBS; 1:100 phospho‐histone H2A.X (Ser139) (20E3) rabbit mAB (9718 s) (Abcam) was added, and the cells were incubated at 4°C overnight. The antibody was discarded, and the cells were rewashed with PBST; 1:500 goat anti‐rabbit IgG H&L (FITC) (ab6717) (Abcam) was added, and the cells were incubated with the cells in the dark for an hour at room temperature. Finally, the cells were washed three times with PBST, Hoechst 33342 counterstain for nuclear DNA (Cell Signaling) was added at a final concentration of 1 μg/μl, and the cells were incubated for 15 min at room temperature. Finally, the cells were washed three times with PBST and visualized by a Zeiss LSM800 confocal laser scanning microscope (Carl ZeissMicroscopy, Oberkochen, Germany). We counted γH2A.X foci using FociCounter software (http://focicounter.sourceforge.net/download.html) following the manufacturer's instructions. The results are reported as the intensity (foci brightness), and the foci brightness of PC‐transfected cells was normalized to 100%. We performed a statistical analysis using a paired‐sample *t*‐test.

### 2.23 Western blot analysis

We transfected cells with the indicated plasmids for 48 h. After that, the cells were washed with 1× PBS and lysed on ice for 45 min with ice‐cold radioimmunoprecipitation (RIPA) buffer (Sigma Chemical) and protease inhibitor mixture (Pierce Biotechnology). The protein content of the protein lysates was analyzed using a BCA protein assay kit from Pierce Biotechnology (Rockford, IL, USA). First, equal amounts of denatured protein samples (20 μg) were loaded onto 5% and 12% SDS–PAGE gels in 1× Tris/glycine/SDS buffer before transferring to 0.45‐μm nitrocellulose membranes in 1× Tris/glycine buffer for 2 h (Bio‐Rad). Then, the transferred membranes were blocked for 1 h in 5% nonfat dry skim milk in TBST (25 mM Tris–HCl, pH 7.5, 125 mM NaCl and 0.05% Tween 20) and incubated overnight with specific primary antibodies against the indicated proteins. Then, membranes were washed three times with TBST and incubated with the appropriate horseradish peroxidase (HRP)‐labeled secondary antibodies (goat anti‐mouse IgG, HRP; 1:2000‐10 000(7076 s) (Cell Signaling), goat anti‐rabbit IgG, HRP; 1:2000‐10 000(7074 s) (Cell Signaling) for 2 h at room temperature. The immune complexes were detected by Immobilon Western Chemiluminescent HRP Substrate (Merck, DA, Germany) and exposed by Azure c300 imaging systems (Azure Biosystems). To measure the expression levels in DDR, the protein was probed with primary antibodies, including phospho‐histone H2A.X (Ser139) (1:1000, mouse mAb, cat: 05–636)(Merck Millipore) and phospho‐ATM (Ser1981) (1:1000, rabbit mAb, cat: 5883 s)(Cell Signaling). For the determination of aging markers and the expression of upstream signaling proteins, the indicated proteins were probed with p16^INK4A^ (1:1000, rabbit mAb, cat: 92803 s)(Cell Signaling), p21 (1:1000, rabbit mAb, cat: 227443)(Abcam), and p53 (1,1000, rabbit mAb, cat: 9282 s) (Cell Signaling).

### 2.24 X‐ray exposure and comet assay

To observe whether Box A is more resistant to ionizing radiation cells were irradiated by x‐ray exposure, and Comet assay were analyzed. X‐ray irradiation was performed at room temperature using a 6‐MV Clinac®iX system linear accelerator (Varian Medical Systems) at a dose rate of 2 Gy/min. Immediately, after irradiation, Comet assay was performed as previously described with some modifications^37^ . Briefly, suspension cells were mixed with 1% low melting point agarose at a ratio 1:10 (v/v), and 30 microliters of the mixture was spread on a slide that was coated with 1% melting agarose. Then, the slides were immersed in lysis solution (2.5 M NaCl, 100 mM EDTA, 10 mM Tris–HCl, 1% Triton X‐100, and 10% DMSO, pH 10.0) at 4 °C for 2 hrs. The slides were soak in alkaline electrophoresis solution (300 mM NaOH and 1 mM EDTA, pH 13.0) at 4 °C for 30 min, and subsequently subjected to gel electrophoresis (50 mA, 30 min). After electrophoresis, the slides were washed with neutralizing solution (0.4 M Tris–HCL, pH 7.5). Finally, the slides were stained with SYBR green for 20 min before visualization. The slides were examined using a fluorescence microscope (Eclipse, Nikon) and images (8–10 fields/slide) were collected. Comet tail length was measured using the NIS Elements software (Nikon).

### 2.25 DNA immunoprecipitation (DIP) of 8‐OHdG

DIP was modified as previously described.^38^ HMWDNA (1–1.5 μg) was sonicated for 7 min with a Bioruptor (30 s on, 30 s off at maximum power) to obtain 300–1000 bp DNA fragments. The sonicated DNA was denatured at 95°C for 10 min and chilled immediately on ice. One‐third of fragmented DNA was used as the input, and two‐thirds of DNA was incubated with 3–5 μg of antibodies overnight at 4°C, including antibodies specific for DNA/RNA damage (8‐OHdG) (Abcam), mouse IgG (Abcam), and rabbit IgG; then, the samples were incubated for 2 h at 4°C with protein G‐Sepharose (GE Healthcare Life Sciences). After washing with phosphate‐buffered saline, DNA was extracted from the Sepharose beads by incubation with DIP digestion buffer and proteinase K overnight at 50°C. The DNA was extracted by the phenol‐chloroform method and resuspended in sterilized dH_2_O.

### 2.26 IRS‐SSB PCR and EDSB‐SSB PCR

HMWDNA was prepared. After polishing with T4 polymerase (New England Biolabs), the 2nd EDSB linker (5‐GGTACCGGTAGGGCCTACGGGTGGTACCAT‐3 and 5′‐ATGGTACCACC‐3′) was ligated to EDSBs using T4 ligase (New England Biolabs). The plugs were then incubated at room temperature. After two nights, the cell plugs were washed, and T4 polymerase was added to fill the ends of the ligated linkers. To convert DNA single‐strand breaks (SSBs) to DSBs, DNA was treated with mung bean nuclease (New England Biolabs) at 30°C for 1 h, and EDTA (0.5 M) was added to stop the reaction. After stopping the nuclease reaction, DNA‐GAP PCR linkers were ligated to mung bean nuclease‐created DSBs using the same protocol as in HMWDNA preparation for DNA‐GAP PCR. Two sets of primers were used to perform modified DNA‐GAP PCR on DNA lesions. The first was an IRS primer using the Alu sequence (5′‐ACTGCACTCCAGCCTGGGC‐3′). The second was the 2nd EDSB linker primer (5′‐ GGTACCGGTAGGGCCTACGGGT‐3′).

### 2.27 Plasmid delivery system using Ca‐P nanoparticles in vitro and a rat model

To deliver the plasmids into the cells and the rat model, each type of plasmid was coated with Ca‐P nanoparticle solution before in vitro transfection and animal administration.^39^ The plasmid's highest effective ratio to Ca‐P nanoparticle solution for transfection was 5 μg of plasmid in 100 μl of Ca‐P nanoparticle solution both in cell culture and in the rat model. The Ca‐P nanoparticle solution was composed of a mixture of 0.5 M calcium chloride (CaCl_2_) solution (Merck Millipore), 0.01 M sodium carbonate (Na_2_CO_3_) solution (Merck Millipore), and 0.01 M sodium dihydrogen phosphate monohydrate (NaH_2_PO_4_·H_2_O) solution (Merck Millipore). The final molar ratio of the CO_3_^2−^/PO_4_^3−^ nanoparticle solution was 31:1. First, the plasmid DNA‐calcium complex was prepared by mixing 16 μl of CaCl_2_ solution and 5 μg of plasmid DNA with the final volume adjusted to 50 μl using sterile dH_2_O. Second, the plasmid DNA‐calcium complex was added to 50 μl of a mixture of Na_2_CO_3_ and NaH_2_PO_4_·H_2_O solution (16 μl) and sterile dH_2_O (34 μl). The nanoparticle‐coated plasmid solution was freshly prepared before transfection or administration. For the rat models, each plasmid type was calculated depending on rat body weight (100 μg of plasmid DNA per kg rat body weight). Then, the plasmid DNA was coated with Ca‐P nanoparticle solution as described above. Finally, the plasmid DNA‐Ca‐P nanoparticle mixture was freshly prepared before an intraperitoneal administration.

We investigated transfection efficiency using the nanoparticle‐coating solution and Ca‐P nanoparticle‐coated Box A‐GFP plasmids by transfecting them into HEK293 cells (5 × 10^4^ cells/well) in a 24‐well plate 24 h after cell seeding. The transfected HEK293 cells were observed, and confocal images were captured at 20× 48 h after transfection using a confocal microscope (ZEISS LSM 800, CARL ZEISS).

### 2.28 Animal study

All animal procedures were reviewed and approved by the Animal Care and Use Committee, Chiang Mai University, Thailand, in conformity with the Association for Assessment and Accreditation of Laboratory Animal Care (AAALAC) guidelines (Approval No. 2562/RT0013 and 09/2563). Twenty‐four male Wistar rats (6–8 weeks of age) were obtained from the National Laboratory Animal Center, Mahidol University, Bangkok, Thailand, and 24 male Wistar rats (10 weeks of age) were purchased from Nomura Siam International, Bangkok, Thailand. After 1 week of acclimatization, all animals were housed in a temperature‐controlled chamber (25 ± 0.5°C) with a 12:12‐hour light/dark cycle until they reached the desired ages (3 months of age for the D‐galactose [D‐gal] study and at 28 months of age for the natural aging study). Standard diet and sterilized water were provided *ad libitum*. All rats were monitored daily and weighed weekly. The desired aged rats of both studies were randomly assigned to subgroups by a veterinarian who was blinded to characteristics of rats. For the D‐gal study, the first group of rats (*n* = 16) was subcutaneously injected with D‐gal (150 mg/kg) (Sigma‐Aldrich) in normal saline solution (NSS) daily for 16 weeks, and the other (normal control) group of rats (*n* = 8) was administered NSS without D‐gal in the same manner. After an 8‐week D‐gal injection period, the D‐gal rats were randomly subdivided into two groups of eight rats per group. Group 1: Normal rats (7 months old) were intraperitoneally injected with 100 μg of pcDNA3.1 per kg body weight (7 m → PC) once a week for 8 weeks. Group 2: D‐gal rats (aging control) were intraperitoneally administered 100 μg/kg pcDNA3.1 (D‐gal → PC), and Group 3: D‐gal rats (treatment group) were also injected with 100 μg/kg Box A plasmid (D‐gal → Box A) in the same manner. For the D‐gal aging study, the rats were subjected to a lateral tail vein bleed of approximately 300 μl at baseline (3 months of age), after 8 weeks of the D‐gal or NSS injection (5 months of age), and after 8 weeks of the treatment (7 months of age). For the natural aging study, the rats were aged in a house until the age of 28 months and then randomly designated into three groups. Twenty‐eight‐month‐old rats were injected with Box A plasmid (30 m → Box A, *n* = 10), pcDNA3.1 (30 m → PC, *n* = 10), and p. F38G (30 m → p. F38G, *n* = 4) for consecutive 8 weeks. Rat blood samples were also collected before and after the treatment period (at 28 and 30 months of age, respectively). The rat sera were shipped to the Hematology and Biochemistry Laboratory, Small Animal Hospital, Faculty of Veterinary Medicine, Chiang Mai University, for analysis of rat liver function parameters. At the end of the treatment, the rats in all experiments were euthanized, and various rat tissues were immediately collected in 4% paraformaldehyde (PFA) (Sigma‐Aldrich) and 10% formalin buffer and snap‐frozen in liquid nitrogen at −80°C for subsequent analyses. All frozen snap tissues were shipped to the Faculty of Medicine, Chulalongkorn University, on dry ice. Visceral fat was weighed and reported as visceral fat (g) per 100 g of rat body weight.

### 2.29 Determination of serum hepatic biochemical parameters

Rat serum samples were shipped to the Hematology and Biochemistry Laboratory, Small Animal Hospital, as mentioned above. Levels of serum aspartate aminotransferase (AST), alanine aminotransferase (ALT), alkaline phosphatase (ALP), total protein (TP), and albumin were measured using an Automated Clinical Chemistry Analyzer (BX‐3010, Sysmex, Japan). Serum globulin levels were calculated from serum TP and albumin levels.

### 2.30 SA‐β‐Gal staining, immunohistochemical staining (IHC), and histopathological analysis

After euthanization, rat tissues were immediately dissected and fixed in a fresh fixative buffer. For SA‐β‐gal staining on liver cryosections, tissue was stored in 4% PFA before embedding in OCT (Sakura, Tissue‐Tek) and cryosectioning at 10 μm thickness. After rehydration of the liver section in PBS, the sections were SA‐β‐gal stained using the Cell Signaling kit (9860, Beverly, MA, USA) with a 15‐min fixation followed by 37°C incubation in the staining solution for 12 h. For IHC and histopathological analyses, tissues were fixed in 10% formalin buffer for at least 48 h. Then, the tissues were dehydrated and paraffin‐embedded before 5‐μm thick tissue sectioning by a microtome. Subsequently, the rat liver and pancreas sections were stained with hematoxylin & eosin (H&E) according to standard procedures for histopathological analysis. For IHC staining, rat spleen and muscle (gastrocnemius) were stained with anti‐DYKDDDDK tag (FLAG) rabbit monoclonal antibody (14793) (Cell Signaling) diluted 1:500. The mounted sections were captured using a Leica DM1000 inverted microscope with a color camera. Masson's trichrome staining was conducted to measure collagen accumulation in the liver according to the manufacturer's standard protocol (Bio‐Optica, 04–010802, Malino, S.p.A. For quantification of SA‐β‐gal in the liver, hepatic sinusoidal space, collagen accumulation in the liver, and islets of Langerhans size, the images were analyzed using ImageJ software (*n* = 3–4 animals per group).

### 2.31 Morris water maze (MWM) performance

Since the motor and anxiety‐like behaviors could impact cognitive assessment results, we first evaluated the modulatory effects of our treatments on locomotor activity and anxiety throughout the experiment using an open field test. We found no significant differences among all groups in the total distance traveled, average speed, and total time spent in the central zone, indicating no potential impact of any motor disorders or anxiety on cognitive assessments. Next, spatial learning and memory were assessed by the MWM test in both D‐gal and natural aging rats at the end of the treatment period. The water maze test was performed using a circular pool 200 cm in diameter filled with water (26 ± 1°C), following Morris's protocol of Vorhees & Williams.^40^ A 10 cm^2^ escape platform was placed in the designated quadrant of the pool and submerged ~1–2 cm below the water surface. The MWM test was composed of two different sessions, including the acquisition test and the probe test. In the acquisition training test, each rat was trained to navigate to the platform four trials per day for five consecutive days. Each rat was given a trial limit of 120 s per trial and guided to the platform when it reached the time limit. The rats were left on the platform for 15 s during the intertrial interval. The escape latency time was recorded from the starting point until the rats reached the platform using the camera. In the probe test (the 6th day), the platform in the target quadrant was removed, and rats were placed in a novel starting position (the opposite of the target quadrant). The time that rats spent in the target quadrant was recorded during the 120‐s testing period. The data analysis was performed from the video files using SMART 3.0 software (Panlab Harvard Apparatus).

### 2.32 Rat liver and brain protein preparation for western blotting assay

Snap frozen liver and brain tissues were thawed and immediately homogenized on ice using ice‐cold RIPA buffer (50 mM Tris pH 7.5, 150 mM NaCl, 10 mM EDTA, 1% NP, 0.1% SDS). Fifty milligrams of each tissue were mixed with 0.5 ml RIPA buffer containing protease inhibitor cocktail (Cat. No. 11836170001) (Roche Diagnostics) and phosphatase inhibitor cocktail (Cat No. 4906845001) (Roche Diagnostics). The protein concentration of the tissue extract was measured using a BCA assay, and the protein expression level was assessed by western blotting analysis, as described above. To quantitate a DNA damage marker in the rat liver, the tissue extract was probed with primary antibody phospho‐Histone H2A.X (Ser139) (1:1000, rabbit mAb) (cat: 9718) (Cell Signaling). For a detection of aging marker and upstream signaling protein expression, the liver protein was probed with primary antibodies, including p16^INK4A^ (1:1000, rabbit mAb) (cat: A0262) (ABClonal), p21 Waf1/Cip1 (1:1000, mouse mAb)(cat: sc‐6246) (Santa Cruz, CA, USA), and p53 (1:1000, rabbit mAb)(cat: 32532) (Cell Signaling), respectively. To assess protein expression levels of neuron markers in brain, the brain protein was detected with primary antibody β3‐Tubulin XP® (1:2000, rabbit mAb)(cat: 5568) (Cell Signaling) and purified anti‐FOX3 (NeuN) (0.05 μg/ml, mouse mAb, (cat: 834502) (BioLegend). We evaluated the synaptic markers in brain protein using primary antibodies including biotin anti‐Synapsin I/II/III (0.01 μg/ml, mouse)(cat: 853705) (BioLegend), purified anti‐Synaptophysin (2 μg /ml, mouse)(cat: 837103), (BioLegend), and purified anti‐PSD95 (0.5 μg/ml, mouse) (cat: 810301) (BioLegend). The memory markers were measured in the brain protein extract using c‐Fos (9F6) (1:2000, rabbit mAb) (cat: 2250) (Cell Signaling) and phospho‐CREB (Ser133) (1:1000, rabbit mAb) (cat: 9198) (Cell Signaling). We also detected the protein expression levels of inflammatory markers consisting of phospho‐NF‐κB p65 (Ser536) (1:1000, rabbit mAb)(cat: 3033)(Cell Signaling), NF‐κB p65 (D14E12) XP® (1:1000, rabbit mAb) (cat: 8242) (Cell Signaling), anti‐iNOS (1:1000, rabbit)(cat: ab15323)(Abcam), and GFAP (D1F4Q) XP® (1:1000, rabbit mAb)(cat: 12389)(Cell Signaling). The secondary antibodies were used as indicated in in vitro experiment. GAPDH (D4C6R) (1:50000, mouse mAb) (cat: 97166) (Cell Signaling) or beta‐actin [AC‐15] (HRP) (1,50 000, mouse mAb)(cat: 49900)(Abcam) was probed and quantitated as a loading control.

### 2.33 NOL test

The assessment of hippocampal‐dependent memory was performed by the NOL test.^41^ In a habituation phase, each rat was placed in a circle box and allowed to freely explore for 10 min. Then, the test in familiarizing phase was performed after 24 h: each rat was allowed to explore the arena containing two similar objects for 10 min. After 24 h, each rat was placed again in the box for the testing phase to encounter two objects, except that one of them was moved to the new location. The testing phase was performed for 10 min. The video during each phase was recorded by using a camera mounted above the box. The percentage of preference index in the testing phase was calculated from the following formula: time with new location/(time with new location + time with familiar location] × 100.

### 2.34 Statistics

We used the Pearson correlation coefficient to determine the correlation between the number of DNA‐GAP and age or aging markers. Student's *t*‐test was used for comparisons between two sets of samples, and one‐way ANOVA followed by post hoc analysis was used for comparisons among multiple groups of samples. Statistical analyses were performed with GraphPad Prism 9.0 (GraphPad Software, Inc.).

## 3 RESULTS

### 3.1 Youth‐DNA‐GAP reduction in various mammalian aging organisms

First, we investigated Youth‐DNA‐GAP reduction in aging cells. We measured the number of Youth‐DNA‐GAPs in 80 humans aged 36 to 81 years, aged rats in D‐gal‐induced aged rats and naturally aged rats, and human kidney HK2 cells undergoing chemically induced senescence. We performed DNA‐GAP PCR, which quantitates DNA gaps or DSBs from IRSs.^13^ In the eukaryotic genome, there are two types of EDSBs: Youth‐DNA‐GAPs and pathological EDSBs. Under homeostatic conditions, the detectable EDSBs are Youth‐DNA‐GAPs.^13^, ^17^, ^35^ In these older adults, the number of DNA gaps were negatively correlated with increasing age (Figure 1B). To decrease confounders between individuals, we followed the 4‐year change of Youth‐DNA‐GAPs in the same individuals. Consistently, these gaps significantly reduced in these individuals (Figure 1C).

For the aging rat, we used two extensively validated models: natural aging and D‐gal‐induced aging. A large body of evidence has demonstrated that D‐gal‐induced aging in rodents had aging characteristics similar to natural aging.^42^, ^43^, ^44^, ^45^, ^46^, ^47^, ^48^ Consistent with the human results, we also found a reduction in Youth‐DNA‐GAPs in D‐gal‐induced aging rats and chronologically aging rats (Figure 1D).

Many compounds, including etoposide, MMS, or H_2_O_2_, have been reported to induce cellular senescence in vitro.^49^ We used these three compounds to induce the senescent cellular feature characterized by SA‐β‐gal, a biomarker for senescent cells. HK2 cells were cultured with these different chemicals from 6 up to 48 h to investigate DNA gaps and SA‐β‐gal. The results showed that decreased Youth‐DNA‐GAPs under these senescent conditions was a time‐dependent manner. Moreover, the reduced gaps have a negative association with SA‐β‐gal (Figure 1E and F).

### 3.2 Generation of Youth‐DNA‐GAPs by Box A of HMGB1

**FIGURE 2:**
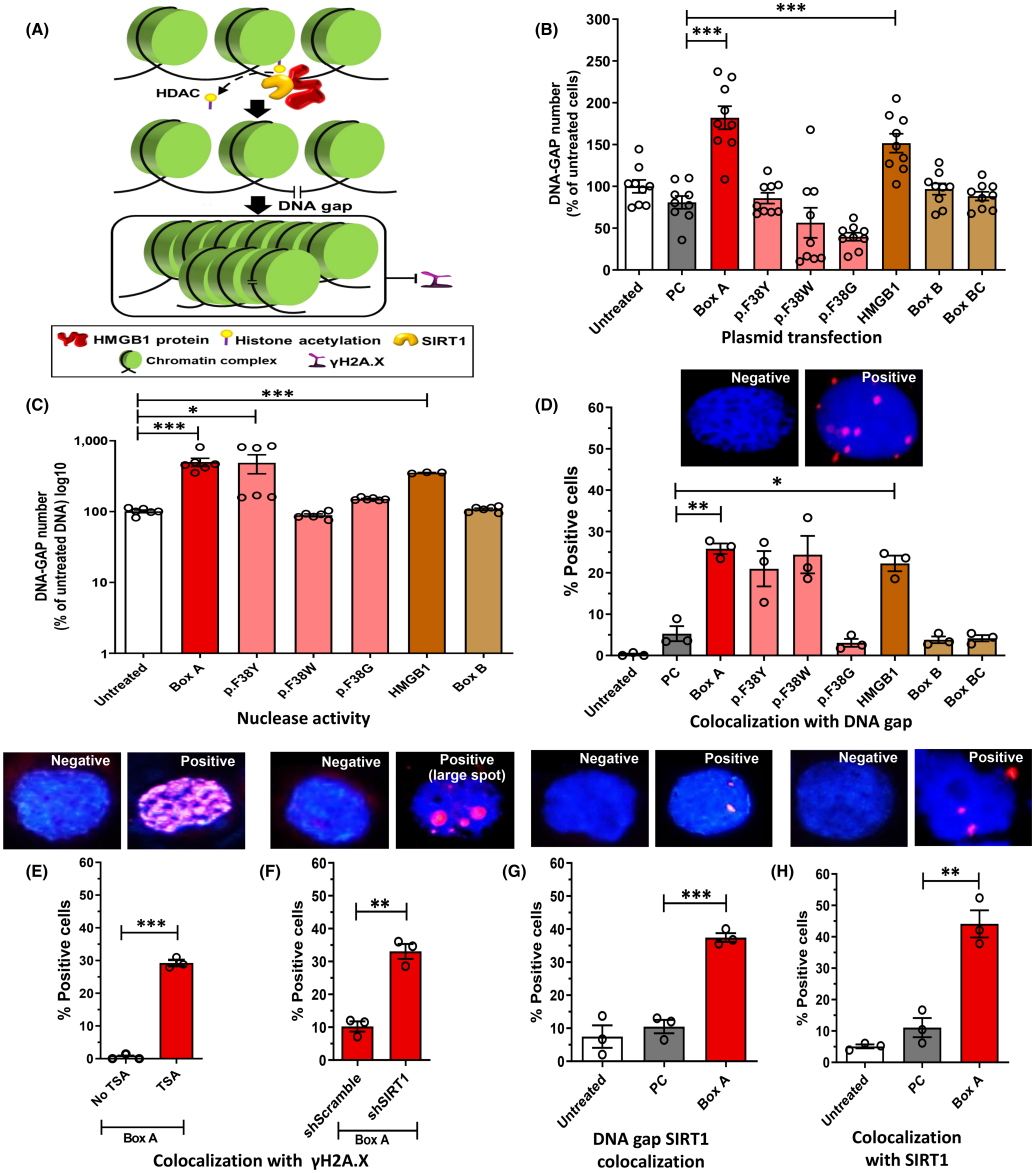
HMGB1‐produced DNA gap formation. We performed seven different experiment aspects to prove that HMGB1 Box A is molecular‐scissors producing DNA gaps, and HMGB1‐produced DNA gaps, similar to Youth‐DNA‐GAPs, are prevented from γH2A.X. (A) Schematic illustration of DNA gap formation. Using series of HMGB1 plasmids or polypeptides, DNA gap formation by plasmid transfection in cells (*n* = 9) (B) and HMWDNA digestion by polypeptides (*n* = 3‐to‐6) (C) were performed. For (C) P‐values were reported if fold change >3. The number of DNA gaps in untreated groups (B) and (C) was normalized to 100%. Colocalization analysis was performed in cells transfected with a series of HMGB1 plasmids. (D‐H) Colocalization analysis using DI‐PLA (D and G) and PLA (E, F, and H). (D) Representative colocalization staining of Flag‐tagged transfected proteins and DNA gaps (*n* = 3). (E) and (F) are the colocalization between Flag‐tagged Box A and γH2A.X. Cells were treated with and without TSA (E), or shScramble and shSIRT (F) then transfected with Box A plasmid (*n* = 3). (G) and (H) Representative colocalization staining of (G) SIRT1 and DNA gaps and (H) SIRT1 and transfected Flag‐tagged Box A (*n* = 3). Representative colocalization staining is indicated by the presence of red spots. PC is a control plasmid. All experimental data were independent biological samples. Data are mean ± SEM. *p ≤ 0.05, **p ≤ 0.01, ***p ≤ 0.001 *t*‐test

**FIGURE 3:**
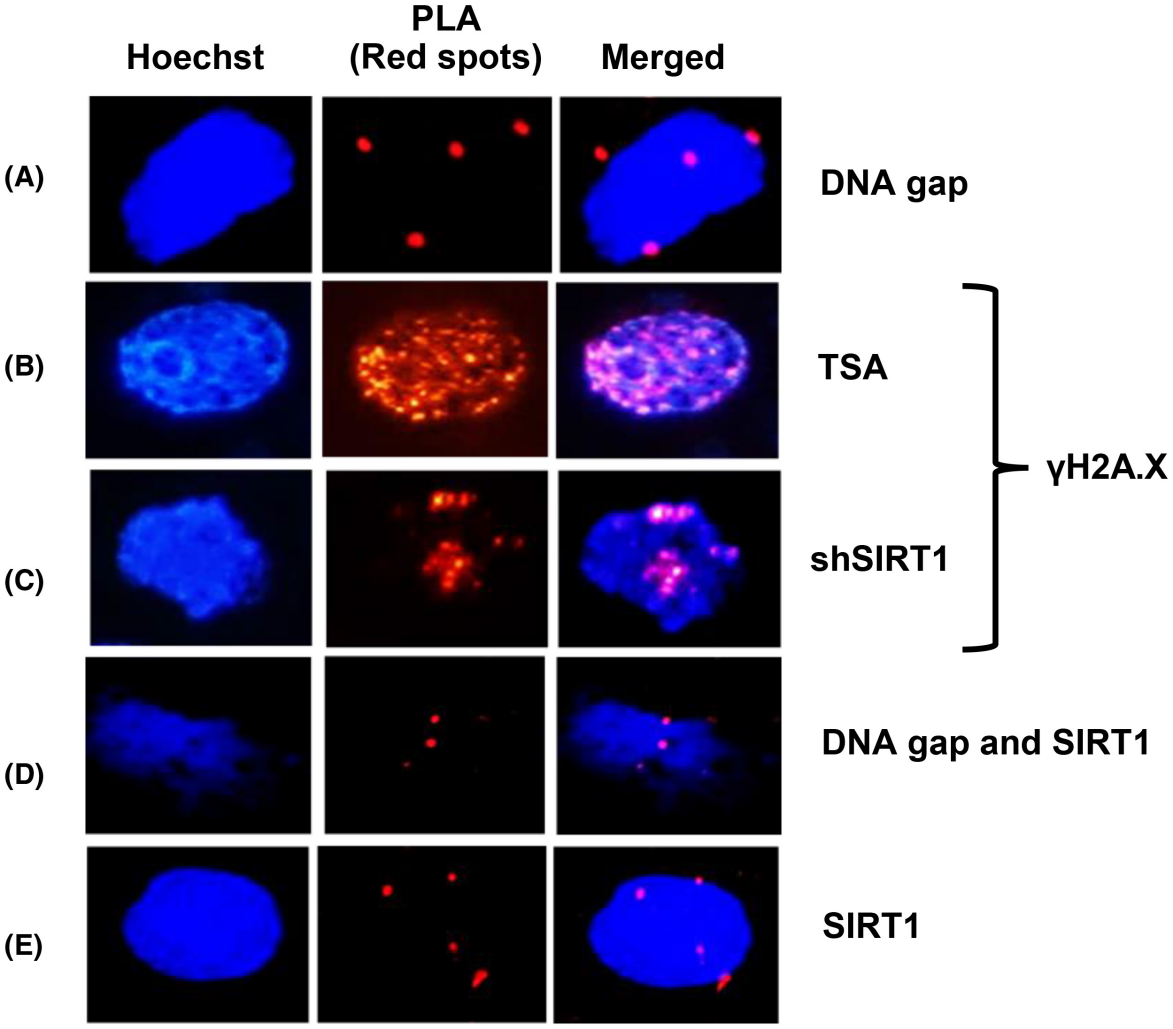
Positive signals in colocalization experiment. HK2 cells transfected with Flag‐tagged Box A plasmid was applied for colocalization analysis with DI‐PLA or PLA indicated by the presence of red spots. The positive signals of colocalization between Flag‐tagged Box A and (A) DNA gaps, (B) γH2A.X of a cell treated with TSA, and (C) γH2A.X of a cell treated with shSIRT1, (D) representative colocalization staining of SIRT1 and DNA gaps and (E) SIRT1 and transfected Flag‐tagged Box A. All experimental data were independent biological samples

We performed several experiments to investigate whether human Box A of HMGB1 acts as a molecular scissor producing Youth‐DNA‐GAPs (Figure 2A–H). We selected‐specific HMGB1 domains (i.e., Box A, Box B, and Box BC) to determine which domains carry Youth‐DNA‐GAP production activity thoroughly. Box A was singly substituted with three missense mutations, p.Phe38Tyr (p.F38Y), p.Phe38Trp (p.F38W), or p.Phe38Gly (p.F38G). These three mutant amino acid structures replaced the aromatic side‐chain functional group of phenylalanine with a similar, less similar, or completely absent side chain, respectively. To test whether these genes produce Youth‐DNA‐GAPs, we transfected these selective gene plasmids into immortalized human kidney cells (Figure S1). We found that the number of DNA gaps increased in the cells transfected with HMGB1 and Box A (Figure 2B). Second, we treated HMWDNA with these selective gene polypeptides, and DSBs were generated by HMGB1 and Box A (Figure 2C and Figure S2). Next, we investigated whether HMGB1 and Box A were located adjacent to DNA gaps in transfected cells by DI‐PLA. The results revealed that HMGB1 and Box A were next to DNA gaps (Figure 2D, 3A and Figure S3). Box B, Box BC, and Box A mutants yielded mostly no significant positive results in all three experiments (Figure 2B‐D), suggesting their low to null efficiency in producing these gaps. Taken together, these findings indicate that Phe38 of Box A possesses the DNA cutting role of Youth‐DNA‐GAP formation.

### 3.3 Prevention of the DSB response

One characteristic of Youth‐DNA‐GAPs is they can avoid DSB response, and the prevention was diminished by a histone deacetylase inhibitor, TSA.^16^ Here, we found that Box A specifically colocalized with γ‐H2A.X in cells treated with TSA by PLA technique (Figure 2E, 3B, Figure S4). The colocalization in cells treated with TSA was less extensive in Box A mutants (Figure S4). In addition, a reduction of Youth‐DNA‐GAPs was found in yeast with SIR2 deletion.^17^ Here, we tested if SIRT1 is within Box A‐produced DNA gap complex and plays a role in γ‐H2A.X prevention. The colocalization between Box A and γ‐H2A.X in cells without SIRT1 was demonstrated (Figure 2F, 3C, Figure S5 and S6). Our results also showed that SIRT1 colocalized with DNA gaps (Figure 2G, 3D Figure S7A,B) and Box A (Figure 2H, 3E Figure S7C,D) in Box A‐transfected cells. These data indicated that similar to the naturally occurring DNA gaps, Box A‐produced DNA gaps also has a defensive mechanism against γH2A.X via histone deacetylation and SIRT1 (Figure 2A).

### 3.4 DNA protection role of Box A

As mentioned above, the HMGB group prevents not only DNA damage, but also DDR.^14^, ^25^, ^27^, ^34^, ^35^ Here, we proposed the role of Box A‐produced DNA gaps in DNA protection. DNA protection refers to the prevention of DNA damage and the subsequent DDR mechanism. In contrast, DNA repair requires the DDR to recognize DNA lesions that signal repair‐associated pathways to remove damaged DNA.^50^

**FIGURE 4:**
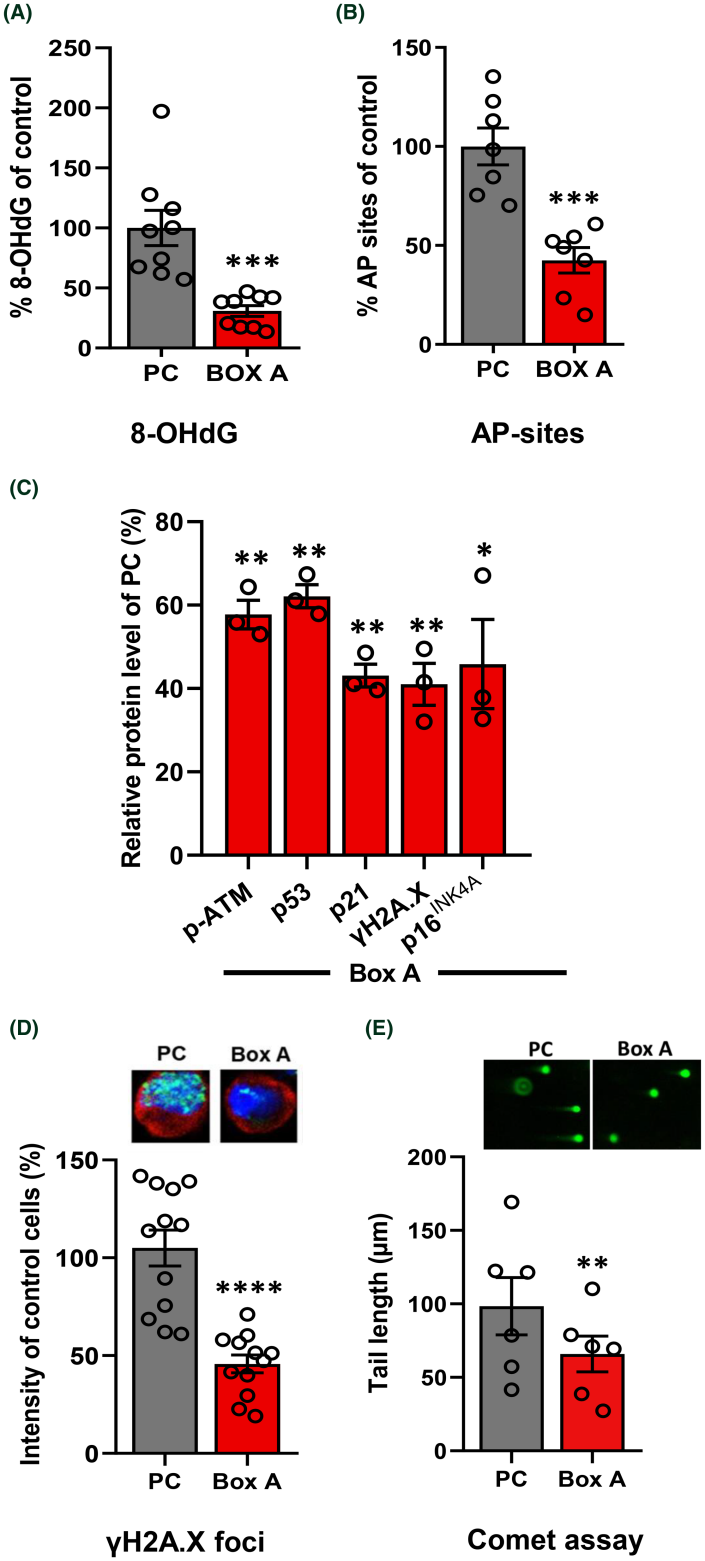
Box A prevents DNA damage and DNA damage response. Endogenous DNA damage, DDR, post‐radiation of γH2A.X foci, and radiation‐induced DSB were measured after Box A transfection. (A), (B) Effect of Box A in comparison with PC plasmids on endogenous DNA damage reduction by measuring the levels of (A) 8‐OHdG (*n* = 9) and (B) AP sites (*n* = 7). (C) Endogenous DDR protein levels of p‐ATM (Ser1981), p53, p21, γH2A.X, and p16^INK4A^ (*n* = 3). The protein levels were compared in pair with the proteins of PC plasmid transfected cells. (D) DSB response prevention determined by γ‐H2A.X foci in cells exposed to x‐ray (*n* = 12). (E) DNA breaks protection by Box A after x‐ray exposure using Comet assay (*n* = 6). Data represent mean ± SEM. *p ≤ 0.05, **p ≤ 0.01, ***p ≤ 0.001, ****p ≤ 0.0001 *t*‐test

First, we confirmed the DNA‐protective effects of Box A by measuring endogenous DNA damage. HMGB1 and Box A plasmids were transfected to two kidney cell lines, HK2 and HEK293 (Figure S1). The results showed that overexpressing Box A had low levels of endogenous DNA damage, as determined by the detection of 8‐OHdG (30%) and AP sites (49%), compared with control PC cells (Figure 4A and B, Figure S8). Moreover, Box A limited endogenous DDR signaling pathway‐associated proteins (Figure 4C, Figure S9). Next, we showed that Box A‐transfected cells resisted DNA break induced by radiation. Box A‐transfected cells had lower number of γH2A.X foci and shorter length of diffuse *tail* behind the nucleuses of Comet assay than control PC transfected cells (Figure 4D and E, Figure S10 and S11). Any Box A mutants lost the capability for genome stabilization (Figure S8‐S10). The percentages of transfected HEK293 and HK2 cell populations in the G0‐G1, S, and G2 phases of the cell cycle were determined by flow cytometry (Figure S12). The flow cytometry result suggested that the outcomes of overexpression of plasmid proteins were not related to cell cycle artifact.

### 3.5 Long‐distance DNA stability spreading from DNA gaps

Given the DNA‐stabilizing functions of Youth‐DNA‐GAPs mentioned above, these gaps should not coexist with DNA damage in the DNA strand. Here, we investigated the distribution patterns of DNA damage and HMGB1‐produced DNA gaps. Under normal physiological conditions, DNA‐GAP PCR can quantitatively detect HMGB1‐produced DNA gaps.^35^ For comparison purposes, we prepared shHMGB1 cells (Figure S13), which lack the HMGB1‐produced DNA gaps. The majority of EDSBs in yeasts without *HMGB1* homolog genes were pathological EDSBs.^14^, ^35^ Therefore, we predicted that DNA damage around each EDSB of shHMGB1 cells should be more common than HMGB1‐produced DNA gaps. We performed two experimental approaches to evaluate genome distribution. The first study of the genome distribution was the association between DNA gaps and 8‐OHdG. In the second experiment, we assessed the distance from a SSB to a DNA gap. We hypothesized that the DNA protection effect spread out along the DNA strand from HMGB1‐produced DNA gaps so that the closer to DNA gaps, the fewer DNA lesions there were.

**FIGURE 5:**
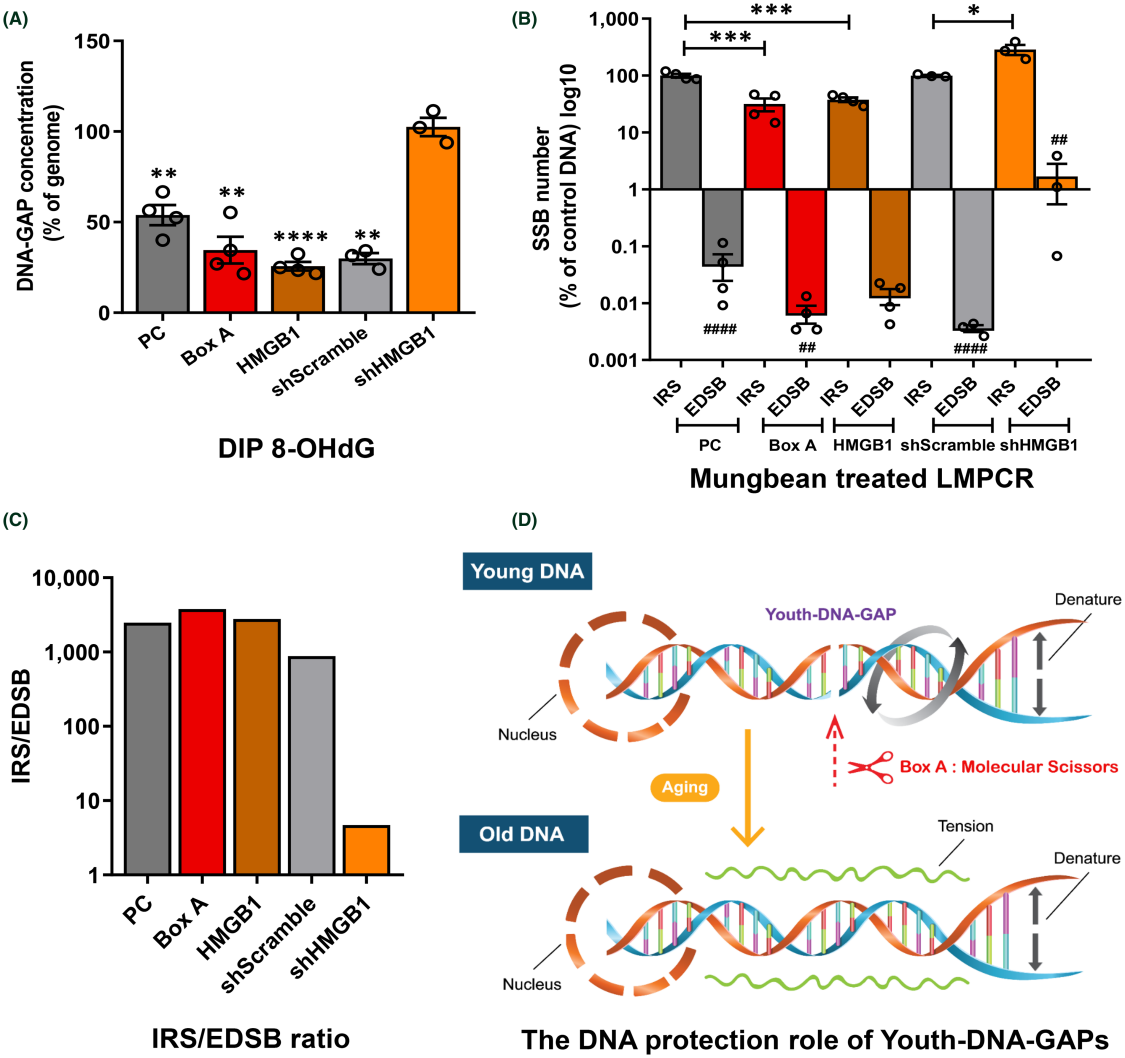
Analysis of DNA damage presents around DNA gaps. DNA of Box A engineered cells were prepared. (A) DNA containing 8‐OHdG was selected by DIP. The concentration of 8‐OHdG‐linked EDSBs measured by DNA‐GAP PCR products from DIP. To calculate the percentage concentrations, the number of DNA gaps or EDSBs (DNA‐GAP number) of the representative genome was normalized to 100. (B) The data show comparisons of two SSB PCRs (IRS‐SSB PCR and EDSB‐SSB PCR) and between IRS‐SSB PCR of Box A‐ and HMGB1‐transfected cells and PC control or shHMGB1 cells and shScramble cells. (C) The IRS/EDSB ratio (proportion of IRS‐SSB PCR and EDSB‐SSB PCR product) in PC (control), Box A, HMGB1, shScramble, shHMGB1. (D) A diagram showing DNA tension with and without Youth‐DNA‐GAP. Data in (B) represent PCR levels of PC and shScramble groups normalized to 100%. Data represent mean ± SEM. *p ≤ 0.05, **p ≤ 0.01, ***p ≤ 0.001, ****p ≤ 0.0001 *t*‐test. ## *p* ≤ 0.01, ##### ≤0.0001 when comparing IRS vs. EDSB. All experimental data were independent biological samples

In the first PCR, we studied the genome distribution association between HMGB1‐produced DNA gaps and DNA damage, 8‐OHdG. If HMGB1‐produced DNA gaps prevent DNA damage, DNA containing DNA damage should have fewer DNA gaps. First, we selected DNA containing 8‐OHdG using a DNA immunoprecipitation technique called DIP. Then, we compared the concentration of DNA gaps or EDSBs between the chosen part of the DNA and the whole genome. Changes in higher or lower DNA‐GAP PCR products of the DIP DNA are associated with higher or lower numbers of 8‐OHdG around EDSB, respectively. The shHMGB1 cells showed different results from all other cases. Except for shHMGB1, the concentrations of DNA gaps or EDSBs from DNA selected by the 8‐OHdG DIP of the other tests were lower than the genomic DNA (Figure 5A). This finding, however, indicates the distribution pattern of 8‐OHdG in which the DNA damage marker is prevented around HMGB1‐produced DNA gaps, suggesting that the DNA gap could play a role in protecting DNA.

Next, we evaluated the distance between DNA damage (SSBs) and HMGB1‐produced DNA gaps. Here, we used *Alu* as an IRS sequence, presenting every 3 kb on average as a DNA mark for comparison purposes. We converted the SSBs to DSBs via mung bean nuclease enzyme, followed by measuring these lesions using ligation‐mediated PCRs from two‐locus types (IRSs and EDSBs) (Figure 5B).^13^ The amount of IRS‐SSB PCR represents SSBs within the PCR range from IRSs, indicating the amount of genome‐wide DNA damage. The IRS‐SSB PCR results confirmed that the DNA protection provided by Box A and HMGB1 plasmids spanned extensively across the genome resulting in 77.4% of the genome depleted in SSB (Figure 5B). In contrast, the damage was elevated in shHMGB1(HMGB1 knockdown) cells (Figure 5B).

EDSB‐SSB PCR reflects DNA damage detected by PCR resulting from EDSBs, and its quantity negatively correlates with the genomic distance between EDSBs and DNA damage. Interestingly, we found that the EDSB‐SSB PCR products of all tests were lower than those of IRS‐SSB PCR (Figure 5B). Thus, EDSBs, on average, were located more distal from DNA damage than IRSs. While the IRS/EDSB product ratios were approximately 1000‐fold higher in most tested cells, that of shHMGB1 cells was just 4.7‐fold higher (Figure 5C). These PCR experiments demonstrated much further distance from DNA damage to HMGB1‐produced DNA gaps than to pathological EDSBs. In conclusion, these experiments indicated that HMGB1‐produced DNA gaps had distal protection in cis against DNA damage to the human genome (Figure 5D).

### 3.6 Rejuvenation of aging cells and rats

Next, we tested the rejuvenating effects of Box A in cultured cells and in naturally aging and D‐gal‐induced rats. Endogenous DNA damage may play roles in driving the cellular aging process by signaling the DDR, so inhibition of proteins in the DDR signaling cascade can rejuvenate aging cells.^1^, ^51^ Therefore, the formation of Box A‐induced DNA gaps, associated with reduced DNA damage and the DDR, may inhibit the senescence process and drive rejuvenation (Figure 1A). Our experiments investigated the rejuvenating effect of Box A using a cell culture system and two animal models. We selected etoposide to develop senescent cells because it induced HK2 cells to become senescent effectively the most (Figure 1E). In the aging rat models, we used two approaches. In addition to the natural aging model, we included D‐gal‐induced aging rats.

**FIGURE 6:**
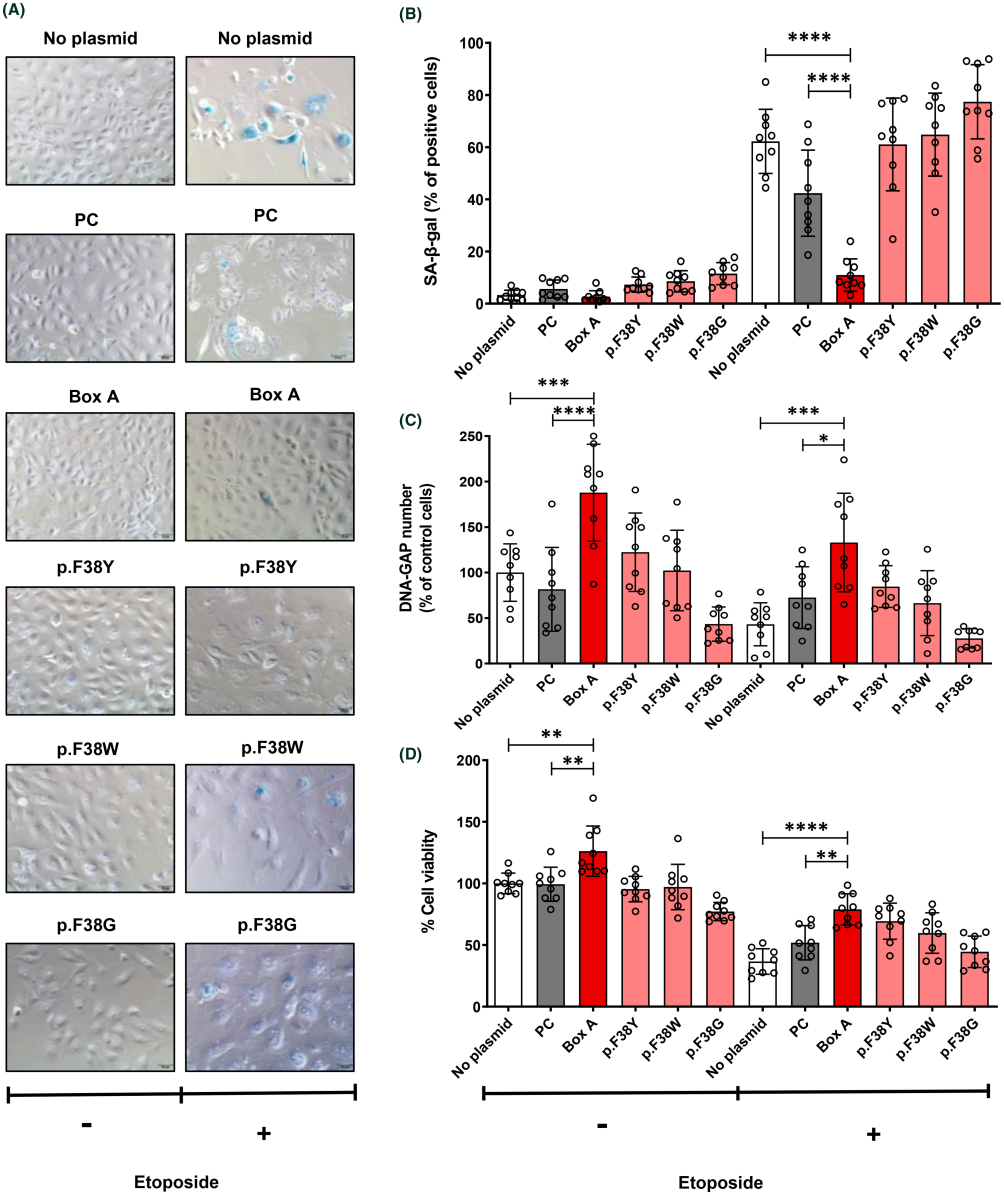
Senescence reversal in vitro. Box A‐produced DNA gaps revitalized senescence cells. We induced senescence‐associated phenotypes in the HK2 cells by etoposide (+) or no (−) treatment for 48 h. Then, the senescent cells were transfected with Box A and Box A mutant expression plasmids compared to PC cells. (A) Cell morphology and examples of SA‐β‐gal‐stained cells (blue color) observed in representative bright‐field images with scale bar = 50 μm. All cells treated with etoposide (+), but not Box A, were enlarged, flattened and stained blue. Box A reversed the effects on cell structure, restoring similarity to all cells in the etoposide‐negative (−) groups. (B) Levels of positive SA‐β‐gal staining (*n* = 9). (C) The number of Youth‐DNA‐GAPs (*n* = 9). (D) Cell viability (*n* = 9). All experimental data were independent biological samples. Data represent mean ± SEM. *p ≤ 0.05, **p ≤ 0.01, ***p ≤ 0.001, **** *p* ≤ 0.0001 *t*‐test

**FIGURE 7:**
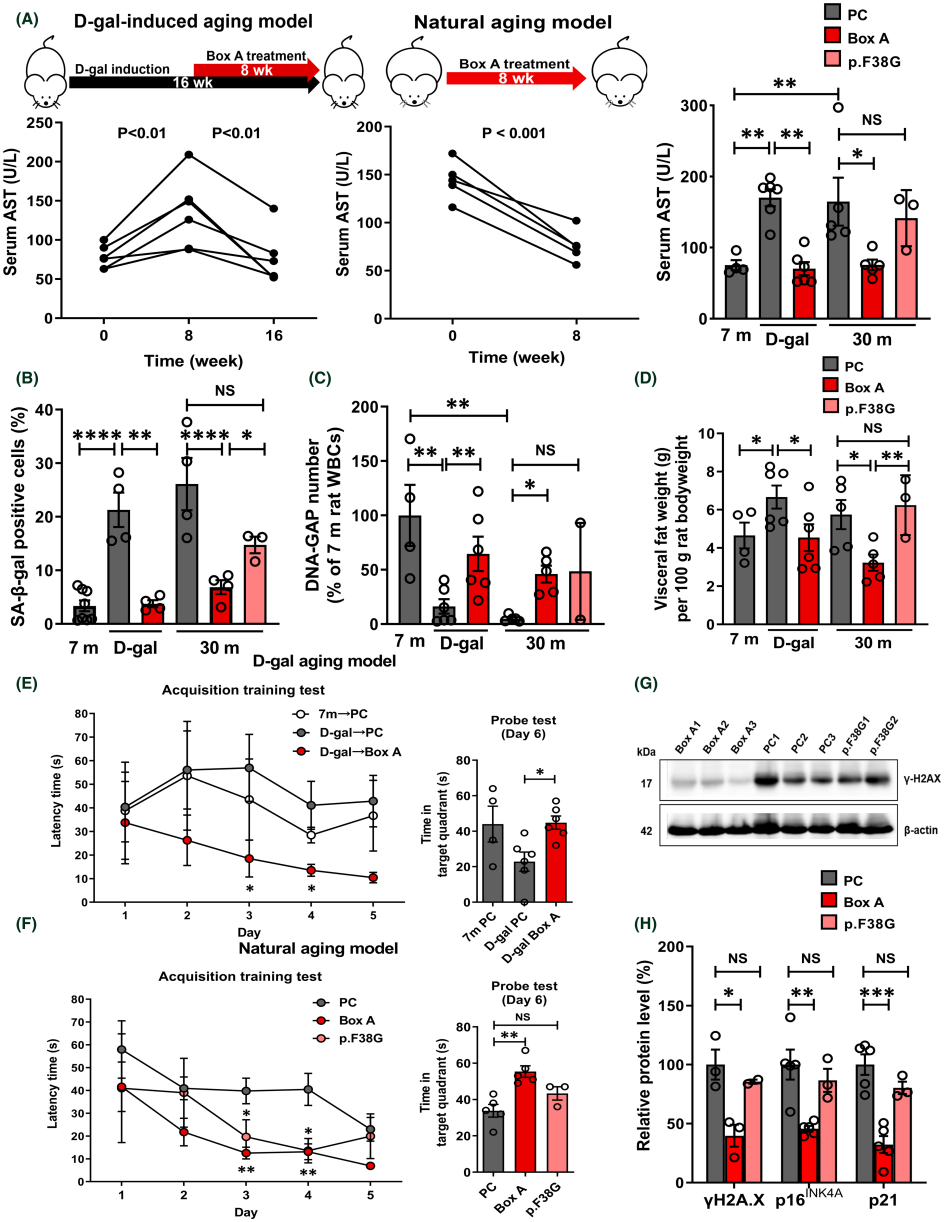
Rejuvenating effects of Box A in D‐gal‐induced and natural aging animal models. Box A‐produced DNA gaps revitalized natural aging and D‐gal‐induced aging rats. Schematic diagrams for (A) D‐gal‐induced and natural aging rats intraperitoneally injected with Box A plasmid/Ca‐P nanoparticle (100 μg of plasmid/kg rat body weight) once a week for 8 weeks compared to PC or p. F38G plasmid treatment. Serum AST levels of the D‐gal aging model (*n* = 6) were determined before D‐gal induction (baseline; rats were 3 months old), after 8 weeks of D‐gal induction, and after Box A treatment (A, right panel). For the natural aging model, the levels of serum AST were measured before (the age of 28 m) and after Box A treatment (*n* = 6) (A, middle panel). Serum AST levels of 7 m, D‐gal, and 30 m treated with PC or Box A plasmids: *N* = 4–6; 30 m with p.F38G: *N* = 3 (A, left panel). SA‐β‐gal staining in rat liver sections quantification (B) is indicated (*n* = 3–4). (C) The number of DNA gaps (DNA‐GAP number) were quantitated in rat WBCs (*n* = 4–6, p. F38G; *n* = 2). (D) Visceral fat (g) per 100 g of body weight (*n* = 5–7, p. F38G; *n* = 3). Morris water maze tests in D‐gal‐induced and natural aging models were assessed, including the acquisition training test (E and F, left panel) and the probe test (E and F, right panel), (*n* = 4–6, p. F38G; *n* = 3). For the natural aging model, (G) immunoblotting levels of γH2A.X and β‐actin proteins in 30 m rat livers (*n* = 2–3) and (H) percentage of relative protein level of γH2A.X, p16^INK4A^, and p21 (*n* = 3–6). The number of DNA gaps in rat WBCs of PC‐treated 7 m rats (D) and the relative protein level in rat liver from the PC‐treated group (H) were normalized to 100%. Paired *t*‐test (A), data represent means ± SEM. *p ≤ 0.05, **p ≤ 0.01, ***p ≤ 0.001, ****p ≤ 0.0001 one‐way ANOVA followed by post hoc analysis, unpaired *t*‐test, and not significance (NS) at 30 m p. F38G versus PC

**FIGURE 8:**
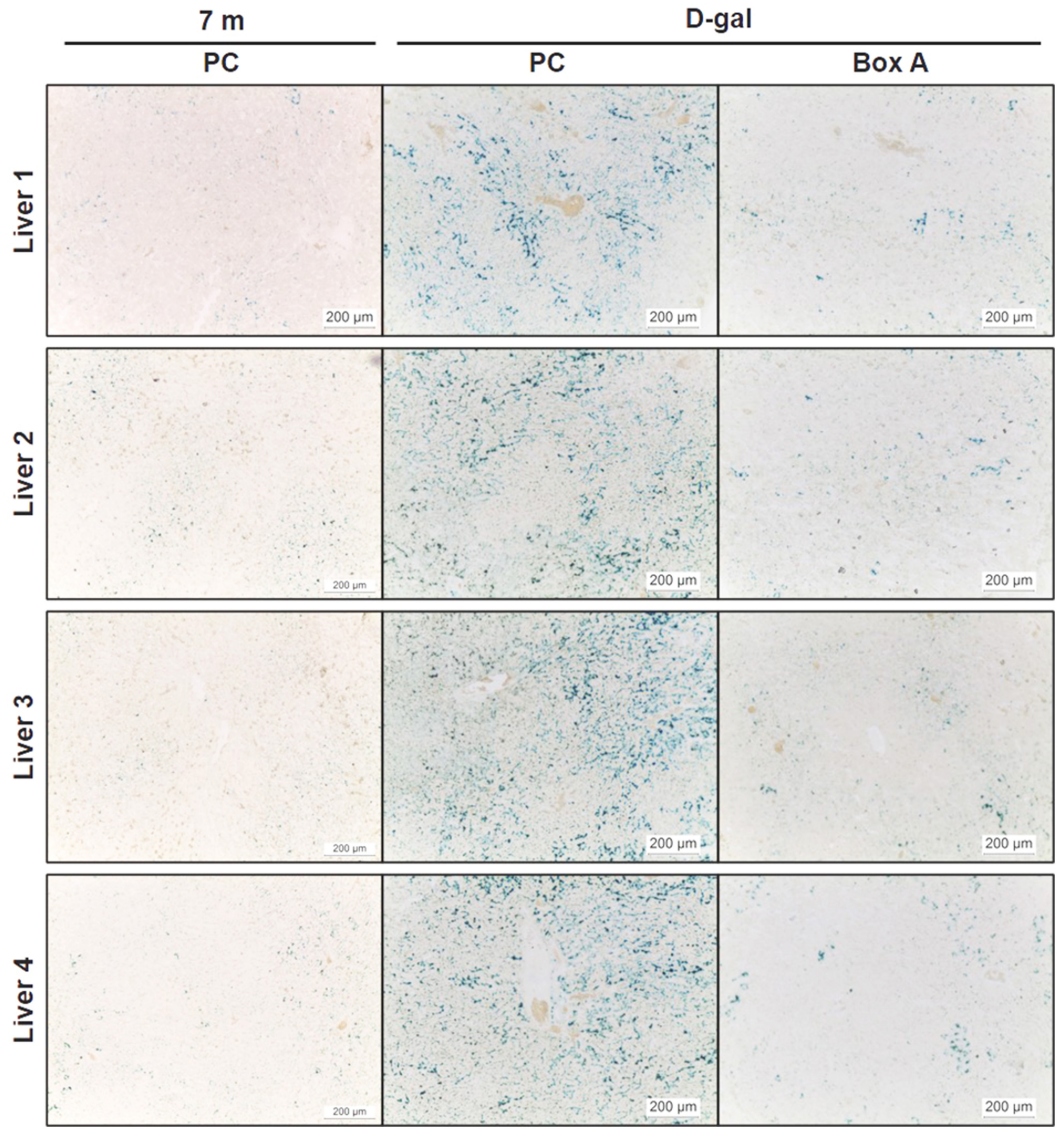
A decrease in an aging marker (SA‐β‐gal) in Box A‐treated D‐gal‐induced aging rat livers. Rat liver sections were stained for SA‐β‐gal activity (blue) to investigate the effect of Box A treatment in D‐gal rats compared to the age‐matched PC‐treated rats and normal groups (*n* = 4 rats per group). The images were captured at 10x magnification

**FIGURE 9:**
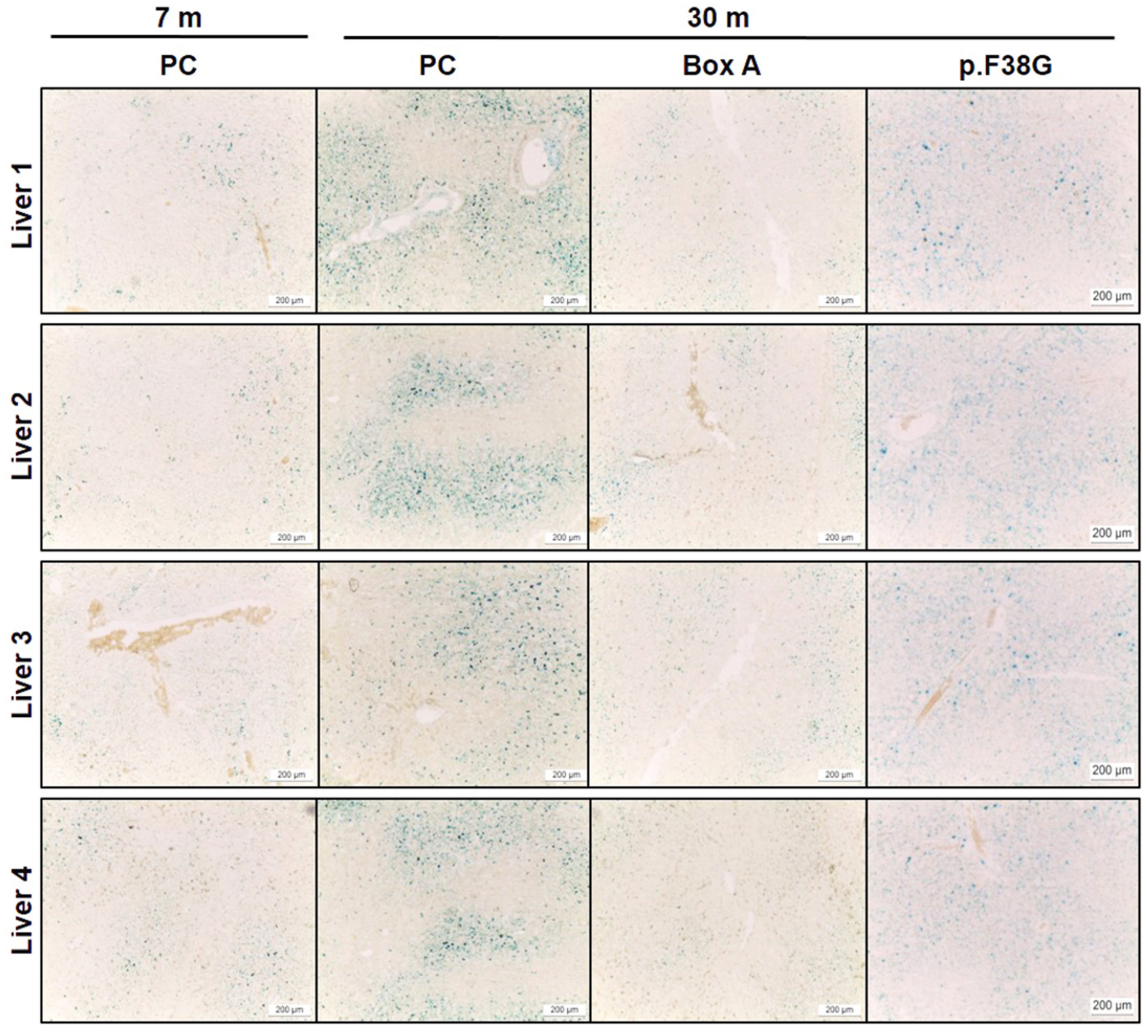
A decrease in an aging marker (SA‐β‐gal) in Box A‐treated naturally aging rat livers. Rat liver sections were stained for SA‐β‐gal activity (blue color) to investigate the effect of Box A treatment in 30‐month‐old rats compared to the age‐matched PC‐ or p.F38G‐treated rats and 7‐month‐old normal groups (*n* = 3–4 rats per group). The images were captured at 10x magnification

**FIGURE 10:**
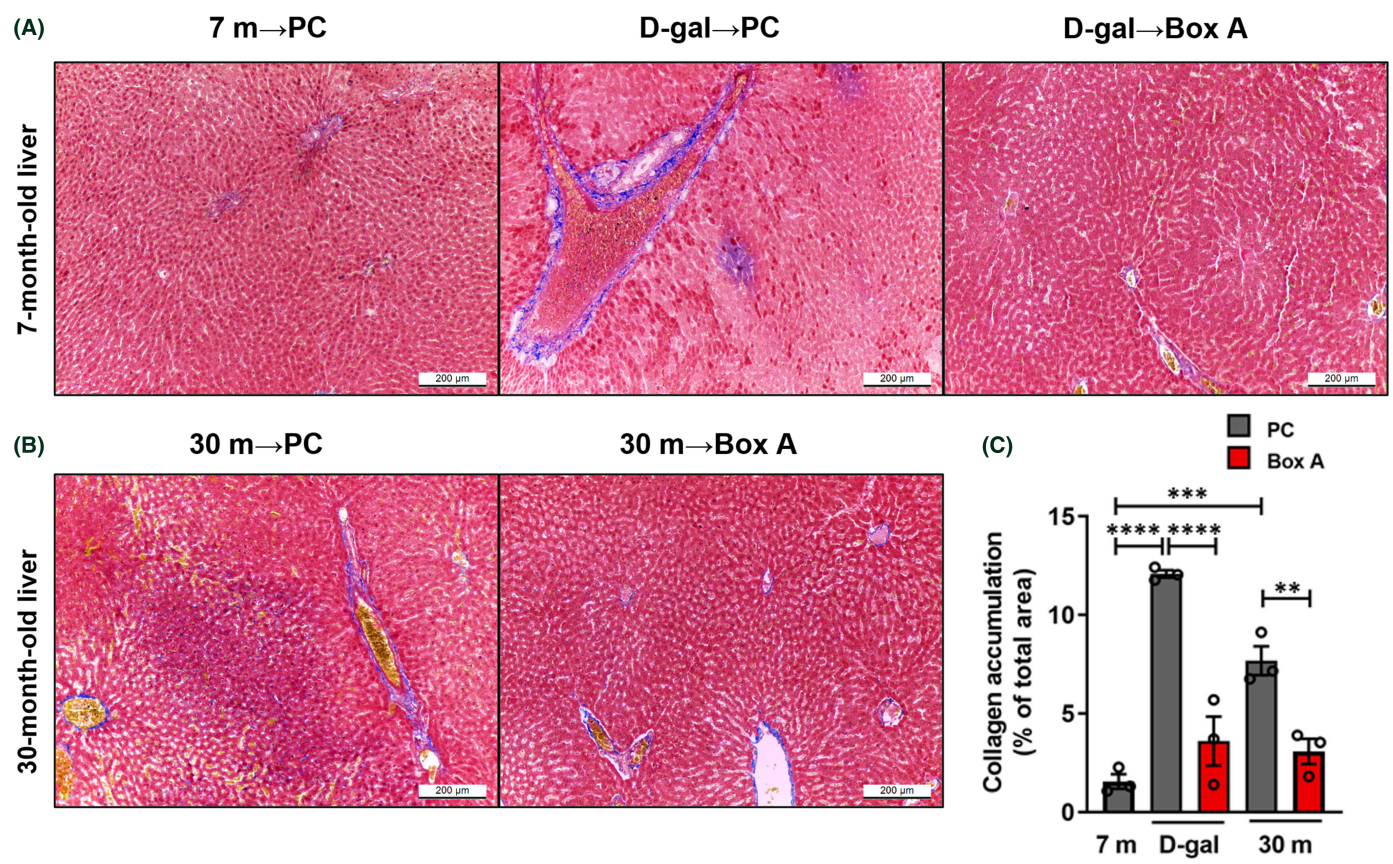
Liver fibrosis reduction by Box A treatment. Microscopic observation of liver sections stained with Masson's trichrome at 200×. Histopathology revealed that fibrosis in the liver was reduced after treating rats with Box A compared to D‐gal and natural aging models. (A) Rat liver sections from PC‐treated normal control (7 m → PC), PC‐treated D‐gal (D‐gal → PC), Box A‐treated D‐gal (D‐gal → Box A) (left to right panel) and (B) PC (30 m → PC), and Box A (30 m → Box A) exhibited accumulation of collagen (blue). (C) Quantification of collagen was performed by ImageJ. Both aging models showed significantly higher collagen accumulation levels in the liver compared to 7 m → PC. After Box A intervention, a significant reduction in collagen deposits was observed in the two aging models. Data represent means ± SEM. **p ≤ 0.01, ***p ≤ 0.001, and ****p ≤ 0.0001 one‐way ANOVA followed by post hoc analysis

The most exciting findings indicate that Box A‐treated groups had elevated Youth‐DNA‐GAPs and effectively improved aging features in senescent cells and aging rats (Figures 6, 7, 8, 9, 10 and Figure S14‐S20). In senescent cells, we demonstrated cell morphology, SA‐β‐gal, Youth‐DNA‐GAPs, and cell viability (Figure 6). The morphology of senescent cells is characterized by enlarged size, flattened cell morphology, accumulation of DNA damage foci, and increased staining of SA‐β‐gal. Whereas Box A reversed the effects on cell structure, restoring similarity to that of all cells in the etoposide negative cells. Box A also significantly reduced SA‐β‐gal, increased Youth‐DNA‐GAPs, and improved cell viability (Figure 6). In both rat models of aging, we investigated Youth‐DNA‐GAPs, DDR, aging biomarkers (SA‐β‐gal, biochemical liver function tests, and senescence‐associated proteins), and aging tissue/functional phenotypes (visceral fat size, histology including liver fibrosis, and size of liver sinusoidal space and islets of Langerhans and learning/memory behaviors) (Figures 7, 8, 9, 10 and Figure S15‐S20). Box A reversed all markers to be closed to the youth (Figures 7, 8, 9, 10 and Figure S15‐S20). We tested the levels of AST, ALT, ALP, TP, albumin, and globulin levels for liver function. Both aging rat models had high levels of serum AST, ALT, and ALP but total protein, albumin, and globulin levels of both aging groups were not different to the youth (Figure 7A, and Figure S15). Box A treatment reduced the levels of serum AST, ALT, and ALP to be closed to the youth (Figure 7A, and Figure S15). Box A did not alter the total protein, albumin, and globulin levels, significantly (Figure 7A, and Figure S15). Box A also changed the levels of the other markers to be closed to the youth. We found a marked decrease in the aging marker SA‐β‐gal of the Box A‐treated rat liver sections (Figures 7B and 8, 9). Figures 8 and 9 demonstrated that Box A treatment consistently decreased the number of SA‐β‐gal positive cells in both D‐gal‐induced and naturally aging rat livers. BoxA reduced the visceral fat in both aging models (Figure 7D). Furthermore, the MWM test showed that Box A treatment exhibited significantly improved learning behavior (latency time) at day 3 and 4 and the restored memory (probe test) in both D‐gal and naturally aging models (Figure 7E and F, respectively).

The formation of Youth‐DNA‐GAPs was significantly elevated in the aging rat DNA, both models, after Box A treatment (Figure 7C). Similar to the previous findings that Youth‐DNA‐GAPs prevent pathological EDSBs,^14^, ^16^ Box A treatment markedly decreased γH2A.X protein levels (Figure 7G‐H and Figure S16). The opposite DNA gaps and DSB response results suggested that Box A‐produced DNA gaps had a protective role against DNA damage at the organ level. Consistently, the accumulation of senescence‐related protein p16^INK4A^ and p21 significantly declined after Box A treatment (Figure 7G‐H and Figure S16). BoxA reduced the accumulation of liver fibrosis in both aging models, sinusoidal space in the D‐gal model, and enlarged pancreatic islets in naturally aging rats (Figure 10 and Figure S17‐S18). Finally, Box A improved aging brain functions by testing Novel object location test (NOL) and additional brain markers associated with inflammation and cognitive memory in the D‐gal rats (Figure S19‐S20).

All Box A mutants were tested for rejuvenating senescent cells, and p.F38G was selected to rejuvenate naturally aging rats. None of the mutant Box A plasmid experiments yield rejuvenation effects to statistically significant levels. It indicates that when Box A mutants lost the capability in producing DNA gaps, they also lost the ability to rejuvenate senescent cells and aging rats (Figures 6, 7, 9 and Figure S15 and S16).

Using two rat models helped validated the effectiveness of Box A in aging biomarker reversal. The D‐gal rat model demonstrated more extensive liver fibrosis and larger liver sinusoidal space (Figure 10 and Figure S17). Most of the other aging features, visceral fat size, liver function test, and number of positive SA‐β‐gal staining liver cells, other senescence associate proteins, learning and memory, of our D‐gal rat model were similar to those in naturally aged rats (Figure 7 and Figure S15‐S16).

## 4 DISCUSSION

This study reported a new mechanism on how DNA ages and invented a technology to treat aging DNA. We demonstrated many pieces of evidence that Box A‐produced DNA gaps prevent DNA damage and the aging process. Adding new Youth‐DNA‐GAPs by Box A of HMGB1 causes the complete rejuvenation of in vitro senescent cells and two aging models in rats in this study.

Similar to other DNA modifications such as 5‐methylcytosine that can be either epigenetic marks or DNA damage, both pathological DNA breaks and physiological DNA gaps are DNA modifications with the same DNA structure; however, pathological DNA breaks are DNA damage, and the physiological DNA gaps are epigenetic marks (Table S1). Most 5‐methylcytosines are epigenetics because they are produced by enzymes and possess physiological functions. However, there may be some 5‐methylcytosines made by methylating agents and considered as DNA damage. These pathologic 5‐methylcytosines are in the wrong cells, interfere cellular function and locations and are precursors of mutation. Previously, we found that the reduction of Youth‐DNA‐GAPs reduced cell viability, and cells lacking one of the HMGB group or SIR2 had a lower number of detectable DNA gaps by PCR. Here in this study, we found that Box A of HMGB1‐produced DNA gaps, and the Box A‐produced DNA gaps stabilized DNA. So these DNA gaps are epigenetic marks because they are made by enzymatic activity and have function benefiting cells. Notably, similar to the other DNA modification, 5‐methylcytosine, no PCR method can distinguish which of these nucleotides are epigenetic marks or DNA damage. However, most 5‐methylcytosines are found non‐randomly and generally interpreted as epigenetic marks under normal circumstances. For DNA‐GAP PCR, we previously evaluated the DNA gap distribution (with DNA methylation and against H2AX) and sequence; we found that under normal circumstance, most DNA‐GAP PCR products were from the physiological DNA gaps (Table S1).^13^, ^16^, ^35^

By evaluating the correlation between Youth‐DNA‐GAPs and age, we concluded that Youth‐DNA‐GAPs are a ubiquitous DNA change existing in a wide range of eukaryotic cells, including yeast, rodents, and humans. Additionally, the reduction of Youth‐DNA‐GAPs varies based on the aging degree and this decrease can result from chemical‐induced or natural aging (Figure 1). The reduction of Youth‐DNA‐GAPs was associated with aging phenotypes regardless of cause. In rats, decreased DNA gaps were found in both natural and D‐gal‐induced aging groups. Similarly, in cells, the DNA gap reduction has been shown in cells exposed to different aging‐inducing chemicals, and the results showed a similar correlation of Youth‐DNA‐GAPs with SA‐β‐gal (Figure 1F). In yeast, a strong correlation was observed between the reduction in Youth‐DNA‐GAPs and viability in aging yeast cells.^14^ So the gap reduction is rather a marker of biological than chronological aging. This study showed a negative relationship between the gaps and the number of senescence cells. Moreover, we found a similar reduction in 30‐month‐old naturally and 7‐month‐old D‐gal‐induced aging rats. Given these consistent data from different eukaryotic organisms, it suggests that the Youth‐DNA‐GAP is a marker of phenotype‐related aging degree. In humans, genome‐wide hypomethylation is also age associated.^52^, ^53^, ^54^ Also, changes in epigenetic marks are associated with the severity of age‐associated NCD.^55^, ^56^ Human Youth‐DNA‐GAPs are linked to methylated DNA.^13^ Therefore, Youth‐DNA‐GAPs may be a novel biomarker indicating the severity of age‐associated NCD.

Here, we performed a number of experiments to demonstrate the Youth‐DNA‐GAP formation roles of HMGB1. The DNA cutting function of HMGB1 is confounded within Box A and is Phe38‐dependent. To demonstrate the DNA cleavage function of Box A, we directly treated DNA with different portions of HMGB1, and DNA restriction by Box A was observed. In addition, we found colocalization between Box A and DNA gap. Moreover, the colocalization results also confirmed the DNA cleaving ability of Box A by demonstrating the colocalization between Box A and γ‐H2A.X when the prevention of DSB response via histone deacetylation or SIRT1 was disrupted. The results also showed that the DNA cutting function disappeared when Box A was mutated at the hypothesized key amino acid.

Moreover, we proved that Box A‐produced DNA gaps can avoid DSB responses similar to the naturally occurring DNA gaps. When SIRT1 was removed, or histone was deacetylated by TSA, the extensive Box A and γ‐H2A.X colocalization results were demonstrated. Notably, the PLA signaling patterns of cells treated with TSA or shSIRT1 differed. While several reports showed that TSA could inhibit class III HDAC,^57^, ^58^ TSA is a known specific inhibitor of class I and II HDACs. Therefore, other HDACs may also help prevent cells to recognize Box A‐produced DNA gaps as pathologic DSB. SIRT1 has been reported to be involved in the prevention of cellular senescence and aging.^59^ Further evaluation if the Youth‐DNA‐GAP retention explains the aging prevention role of SIRT1 is suggested.

There are three types of DNA damage: base change, base loss, and strand breaks. Here, we showed that Box A reduced endogenous 8‐OHdG (most common base change), endogenous AP sites (base loss) and endogenous SSBs, and prevented radiation‐induced DSBs. The DNA protection effect of Box A expression plasmid expanded to a large proportion of the whole genome. The ability to prevent all kinds of DNA damage suggests that Box A plays a role in altering DNA structure rather than enzymatic activity in DNA repair. The capability of Box A in preventing radiation‐induced DSBs and the DSB response against the DNA breakage insult indicates that Box A fortified DNA durability. Any Box A mutants lost the DNA protection capability for genome stabilization, and DIPPCR demonstrated preferentially reduction of DNA damage around DNA gaps. Thus, the fully protective function of Box A requires an intact DNA gap formation function.

The flexibility introduced into DNA structure by Youth‐DNA‐GAPs may be the underlying mechanism protecting DNA from insults (Figure 5D). The double helix structure of DNA forces DNA to twist when bending.^60^ Producing DNA gaps explains how HMGB1 can bend DNA and stabilize DNA against denaturation simultaneously.^21^ When the DNA ends were fixed, its movement energy from any mechanism causing DNA denaturation caused a twisted wave and torsional force to destabilize DNA.^60^, ^61^, ^62^, ^63^ Box A‐produced Youth‐DNA‐GAP's physical structure allows DNA to move to relieve the torsional force from the traveling twist wave (Figure 5D). The tightened double helix hydrogen bonds strengthen DNA to counteract any mechanisms altering chemical bonds in DNA, leading to increased DNA resistance against environmental insults and low numbers of DNA lesions in the genome (Figure 5D). Therefore, one of the crucial roles of Youth‐DNA‐GAPs is to prevent genomic instability, similar to visualizing a small gap in railway tracks. Our data indicated that human DNA gaps are not only youth associated, but also prevent genomic instability. Therefore, human DNA gaps can be termed “youth‐associated genome‐stabilizing DNA gaps” (Figure 5D).

HMGB1 has been widely studied for its role as a secretory protein that induces inflammation and senescence,^31^, ^32^, ^33^ perhaps mainly because of the established molecular mechanism. However, a few reports demonstrated a role in preventing DNA damage.^14^, ^25^, ^27^, ^34^, ^35^ This aspect has not been extensively investigated due to the lack of DNA protection‐related mechanistic information. Moreover, most HMGB1 findings involve its conservative intact form that can adversely function in response to inflammation. Here, we used genetically engineered selected part of HMGB1, containing essential function in producing and maintaining DNA gap, to prove that the select HMGB1 Box A can protect DNA due to DNA gap formation function. For these reasons, this study will facilitate future studies in the field.

There are logical connection pieces of evidence that Box A‐produced DNA gaps may play a role in reversing aging. First, DNA gaps protect DNA. Our previous study done by reducing the DNA gap without reducing any HMGB group gene functions in yeast showed that DNA gaps prevent DNA damage.^14^ The physical mapping between DNA gap and DNA damage analyses showed that the DNA durability spread from the DNA gaps (Figure 5). Second, DNA gaps prevent aging. Aging yeast, human, rat, and senescence cells had a low number of DNA gaps. Moreover, the yeast study reported that reduction of Youth‐DNA‐GAP promoted aging.^14^ Third, DNA damage promotes aging while DDR inhibition leads to rejuvenation. Several reports demonstrated that DNA damage and DDR drive cellular senescence, and inhibition of DDR, notably P16, resulted in rejuvenation.^51^, ^64^, ^65^, ^66^, ^67^ We reported that Box A increased DNA gaps, reduced endogenous DNA damage predominantly around DNA gaps, limited DDR, including P16, and rejuvenated senescence cells and rats. These reasons support the DNA protection and a cellular senescence halt role of DNA gaps created by Box A.

However, our experiments did not exclude other possible molecular mechanisms involving the rejuvenation effect. HMGB1 has a variety of activities intranuclear, in the cytoplasm, and extracellular.^68^ So there may be other potential mechanisms that Box A promoted cellular rejuvenation. For example, the extracellular roles of Box A inhibit inflammation induced by HMGB1.^69^

Box A reversed all these aging features of both models to features associated with younger organisms. Therefore, the rejuvenating effect of Box A is highly effective regardless of the causes of the aging process. These results demonstrated the potential impact of using Box A to treat age and DNA damage‐associated diseases. Aging and DNA damage are associated with the occurrence and severity of many diseases, including common NCDs and infections, such as COVID‐19.^5^, ^70^ Because of the role of Youth‐DNA‐GAPs in DNA damage prevention and aging marker reversal, it is reasonable to hypothesize that reducing the gaps mediates the pathogenesis of these diseases. Moreover, introducing new Youth‐DNA‐GAPs is a promising intervention for patients suffering from aging‐associated diseases.

In conclusion, this study revealed a novel finding of how DNA ages and developed an innovative approach to aging rejuvenation. The number of Youth‐DNA‐GAPs are a biomarker for youthful DNA. HMGB1 Box A, a molecular scissor producing DNA gaps, is a technology to increase DNA durability and reverse aging molecular and physical phenotypes to be close to youths. DNA damage and cellular senescence can cause health deterioration in the elderly.^5^, ^71^ Whereas DNA damage drives cellular senescence, Box A produces DNA gaps. The gaps improve DNA durability, reduce DNA damage and DDR, and rejuvenate DNA. Future studies on the homeostasis and dynamics of organ rejuvenation after Box A treatment are essential. We hope this innovation will help us understand the aging process and aged‐associated diseases pathogenesis. Therefore, Box A is a novel genomic stabilizing medicine that can be a realistic hope for revitalizing many organismal aging features and maybe an intervention that revolutionizes our aging society.

## CONFLICT OF INTEREST

Authors declare that they have no competing interests.

## AUTHOR CONTRIBUTIONS

Conceptualization: AM. Methodology: SY, PW, NB, JT, CP, SiS, KC, WK, RW, MaP, MoP, SuS, SC, and AM. Investigation: SY, PW, NB, JT, CP, SiS, KC, WK, AK, MaP, CS, PP, MO, MoP, SuS, TTO, BA, and WP. Visualization: SY, PW, NB, JT, CP, KC, WK, MoP, SuS, and AM. Funding acquisition: AM. Project administration: AM. Supervision: AM, DJ, SuS, SC, and NC. Writing – original draft: AM, JT, NB, PW, KC, CP, SiS, SY, and SuS. Writing – review & editing: AM, SY, MoP, SuS, and SC.

## Supporting information


Figure S1‐20

Table S1

## Data Availability

All data are available in the main text or the supporting information materials.
