# Erratum to “The roles of HMGB1‐produced DNA gaps in DNA protection and aging biomarker reversal”

**DOI:** 10.1096/fba.1365

**Published:** 2023-01-25

This erratum corrects the following:

Yasom, S., Watcharanurak, P., Bhummaphan, N., Thongsroy, J., Puttipanyalears, C., Settayanon, S., Chalertpet, K., Khumsri, W., Kongkaew, A., Patchsung, M., Siriwattanakankul, C., Pongpanich, M., Pin‐on, P., Jindatip, D., Wanotayan, R., Odton, M., Supasai, S., Oo, T. T., Arunsak, B., Pratchayasakul, W., Chattipakorn, N., Chattipakorn, S., Mutirangura, A. (First published March 9, 2022) The roles of HMGB1‐produced DNA gaps in DNA protection and aging biomarker reversal. *FASEB BioAdvances*. 2022;4:408–434. doi: 10.1096/fba.2021‐00131

The authors report that inadvertent errors were made in assembling two of the figures submitted for publication. In both Figures 8 and 9, the left‐hand row labels “Liver 1” to “Liver 4” are misleading and should not have been inserted. In Figure 8, the images in the 7m PC column should be the same as those shown in the 7m PC column in Figure 9, as the same group of rats is represented in both figures. In Figure 9, a different view of the image in row two of the 30m PC column was inadvertently placed in row four of the 30m PC column. The authors apologize for these oversights and for any confusion caused by the errors. These errors do not affect the results and conclusions reported in the article.

The correct versions of Figures 8 and 9 are as follows:

**FIGURE 8:**
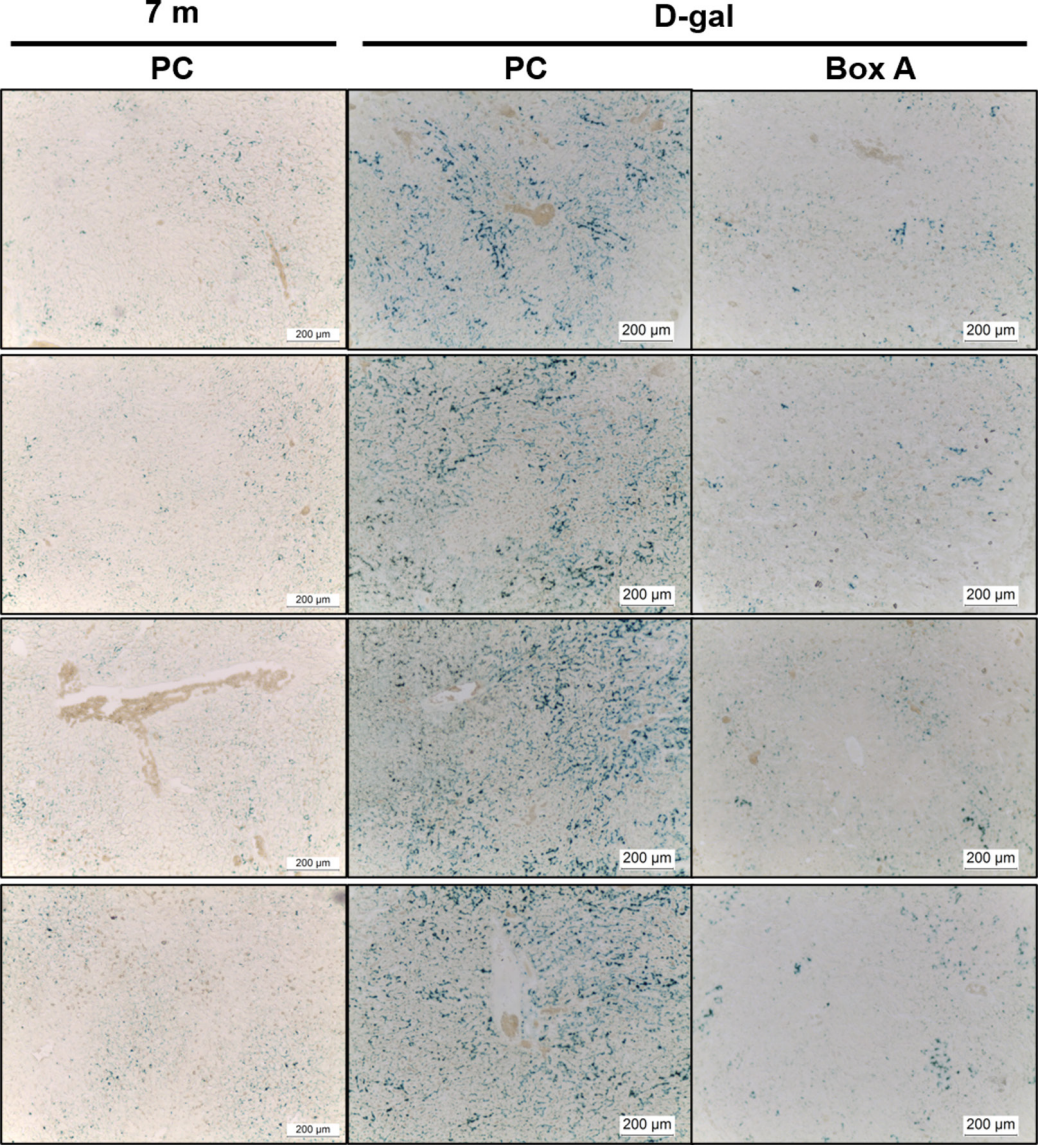
A decrease in an aging marker (SA‐β‐gal) in Box A‐treated d‐gal‐induced aging rat livers. Rat liver sections were stained for SA‐β‐gal activity (blue) to investigate the effect of Box A treatment in d‐gal rats compared to the age‐matched PC‐treated rats and normal groups (*n* = 4 rats per group). The images were captured at 10× magnification.

**FIGURE 9:**
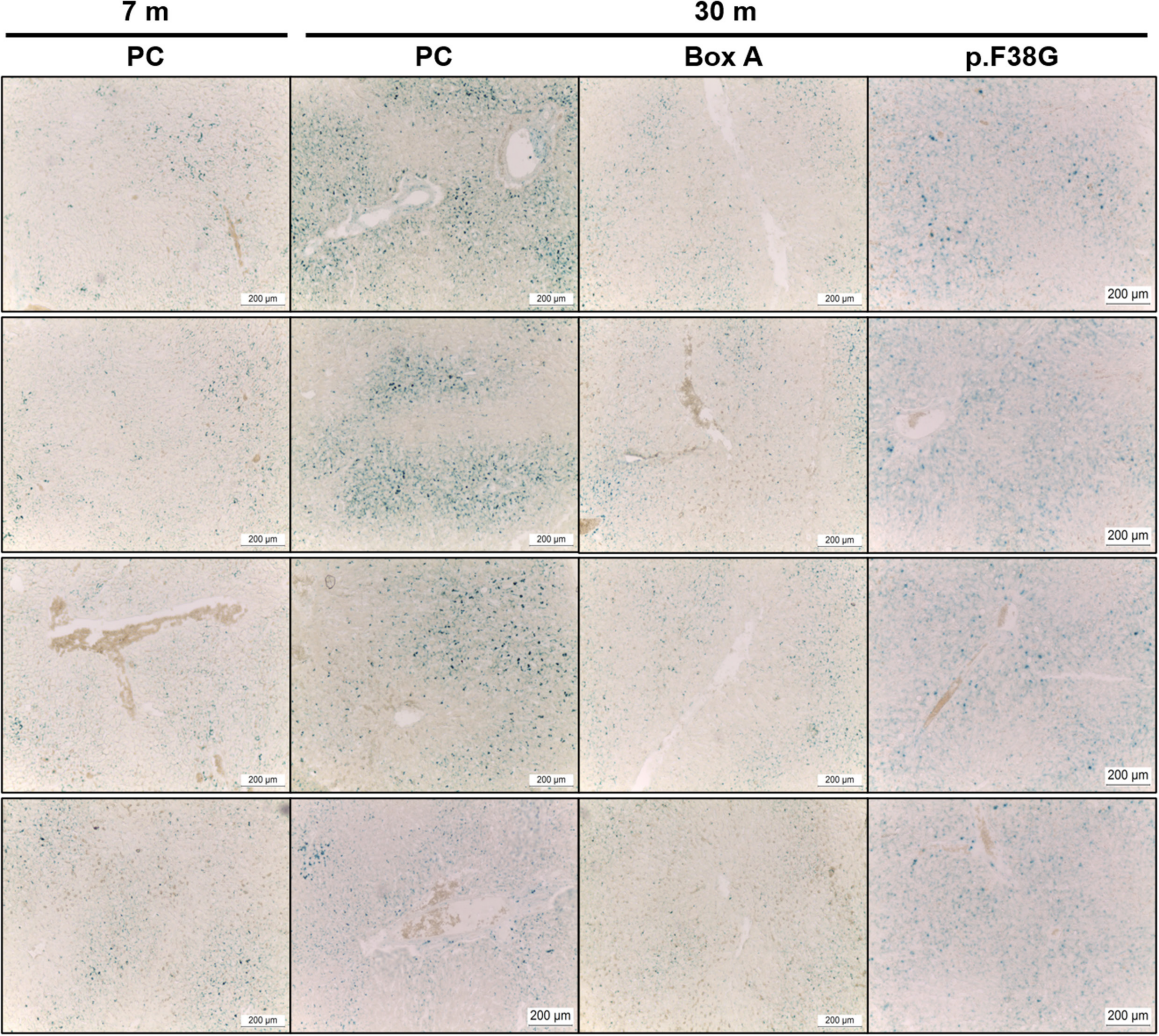
A decrease in an aging marker (SA‐β‐gal) in Box A‐treated naturally aging rat livers. Rat liver sections were stained for SA‐β‐gal activity (blue color) to investigate the effect of Box A treatment in 30‐month‐old rats compared to the age‐matched PC‐or p.F38G‐treated rats and 7‐month‐old normal groups (*n* = 3–4 rats per group). The images were captured at 10× magnification.

