# Supplementary material for: The roles of HMGB1‐produced DNA gaps in DNA protection and aging biomarker reversal
Source: FASEB Bioadv. 2022 Mar 28;4(6):408–34. doi: 10.1096/fba.2021-00131 (PMC9164245; doi:10.1096/fba.2021-00131)
Supplement: Supplementary file 1 — Figure S1‐20 [file FBA2-4-408-s001.pdf]

Supplementary information materials

Figure S1. Engineered HMGB1 protein expression and localization.

Figure S2. The DNA restriction enzyme properties of the HMGB1 protein and Box A polypeptide.

Figure S3. Box A colocalization with the DNA gap result pattern of Figure 2D.

Figure S4. The pattern of Box A colocalization with γH2A.X in trichostatin A (TSA)-treated cells (additional data for Figure 2E.)

Figure S5. Western blotting analysis of shSIRT1 cells.

Figure S6. The pattern of colocalization of Box A and γH2A.X in SIRT1 knockdown cells (additional data for Fig. 2F.)

Figure S7. The results of colocalization experiments on SIRT1 and DNA gap and SIRT1 and Box A (additional data for Fig. 2G and 2H).

Figure S8. Reduction of endogenous DNA damage. Extended results of Fig. 4A and 4B with Box A mutants and HMGB1.

Figure S9. Reduction of DDR. Extended results from Fig. 4C with Box A mutants and HMGB1.

Figure S10. Reduction of γH2A.X foci. Extended results from Fig. 4D with Box A mutants and HMGB1.

Figure S11. Detection of DNA damage by Comet assay after exposure to x-ray.

Figure S12. The percentages of transfected HEK293 and HK2 cell population in the G0-G1, S and G2 phases of the cell cycle.

Figure S13. Western blotting analysis of shHMGB1 cells in Fig. 5.

Figure S14. Box A with GFP-expressing HEK293 cells 48 hours after Ca-P nanoparticle transfection.

Figure S15. Improvements in serum liver function parameters by Box A treatment.

Figure S16. Aging marker protein reduction by Box A treatment.

Figure S17. Reduced D-gal increased sinusoidal space under Box A treatment.

Figure S18. Islets of Langerhans sizes of naturally aging rats reduced by Box A treatment.

Figure S19. The effect of Box A on the hippocampal-dependent learning and memory of normal rats and D-gal-induced aging rats.

Figure S20. Box A treatment restores memory and decreases proinflammatory proteins but does not affect the number of neurons or synapses in the brains of D-gal-induced aging rats.

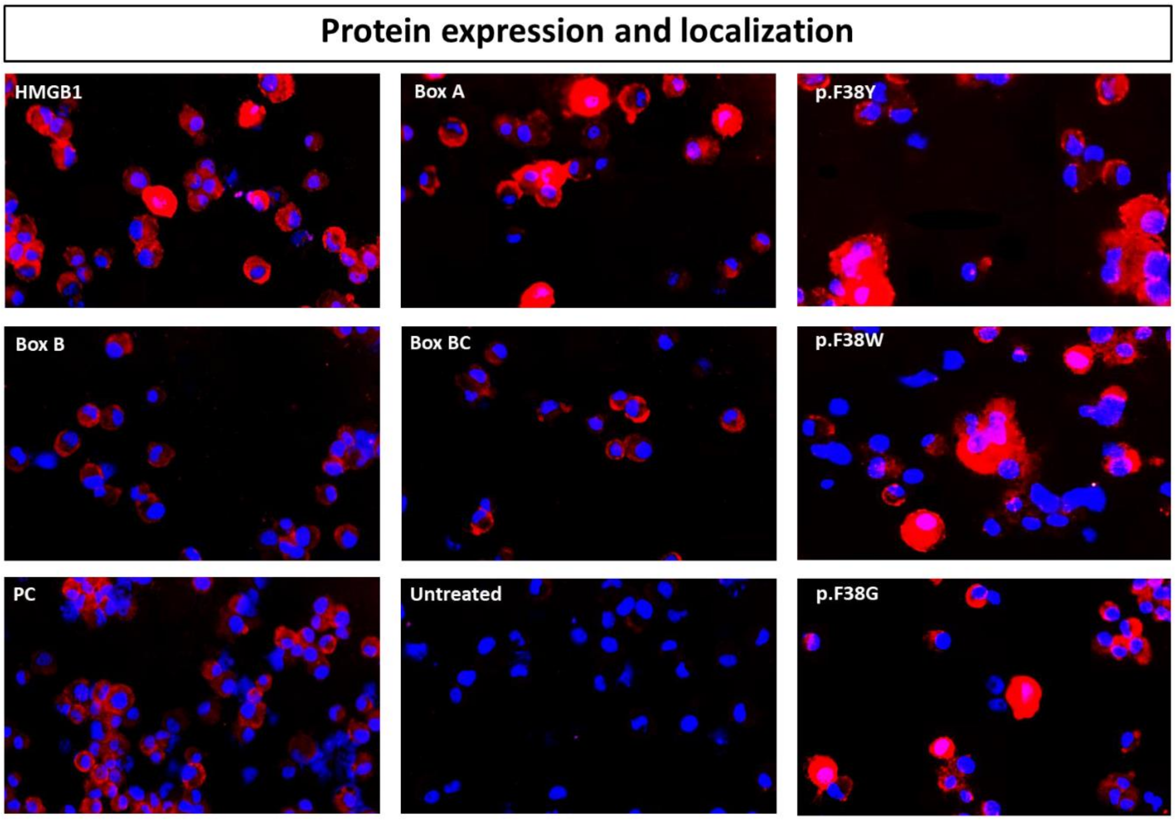

Figure S1. Engineered HMGB1 protein expression and localization. We demonstrated transfection efficiency. Protein expression and localization in transfected HK2 cells were observed with confocal microscopy using a 20x objective lens. The plasmid protein expression signal was observed using an anti-DDDDK tag (FLAG) mouse monoclonal antibody, represented in red (Cy3). HK2 cells were transfected with HMGB1, Box A, p.F38Y, p.F38W, p.F38G, Box B, Box BC, and PC plasmids. HMGB1-, p.F38Y-, p.F38W-, p.F38G- and Box A-transfected cells showed protein localization in the nucleus and cytoplasm. Box B-, Box BC-, and PC-transfected cells showed protein localization only in the cytoplasm. Cells without transfected plasmid (untreated cells) showed a negative signal for plasmid protein expression.

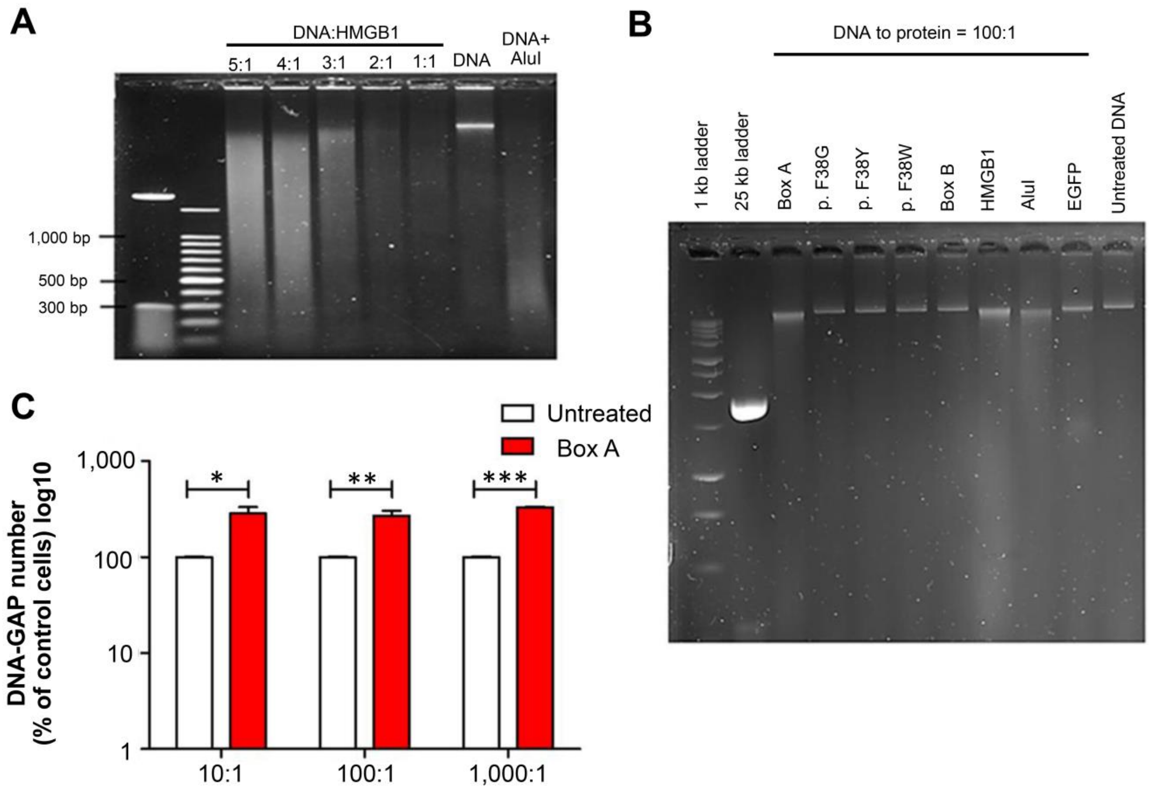

Figure S2. The DNA restriction enzyme properties of the HMGB1 protein and Box A polypeptide. (A and B). To confirm the DNA restriction enzyme properties (Fig. 2C), HMGB1, Box A, Box A mutants, and Box B polypeptides were incubated with HMWDNA; DNA fragmentation was observed using 1% agarose gel electrophoresis. Lysed DNA was demonstrated in smeared bands, while bulk DNA without digestion was sharp bands at the HMW level. Untreated DNA and EGFP incubated DNA were used as negative control groups, and AluI-incubated DNA was used as a positive control. **(C)** Box A peptide was incubated with DNA at various weight ratios of DNA per peptide, and the number of DNA cut sites (DNA-GAP number) was measured using real-time PCR. The data shown are representative of independent biological samples (n=3). Means of DNA-GAP number of untreated groups were normalized to 100%. Data represent mean ± s.e.m. *P ≤ 0.05, **P ≤ 0.01, ***P ≤ 0.001 t-test.

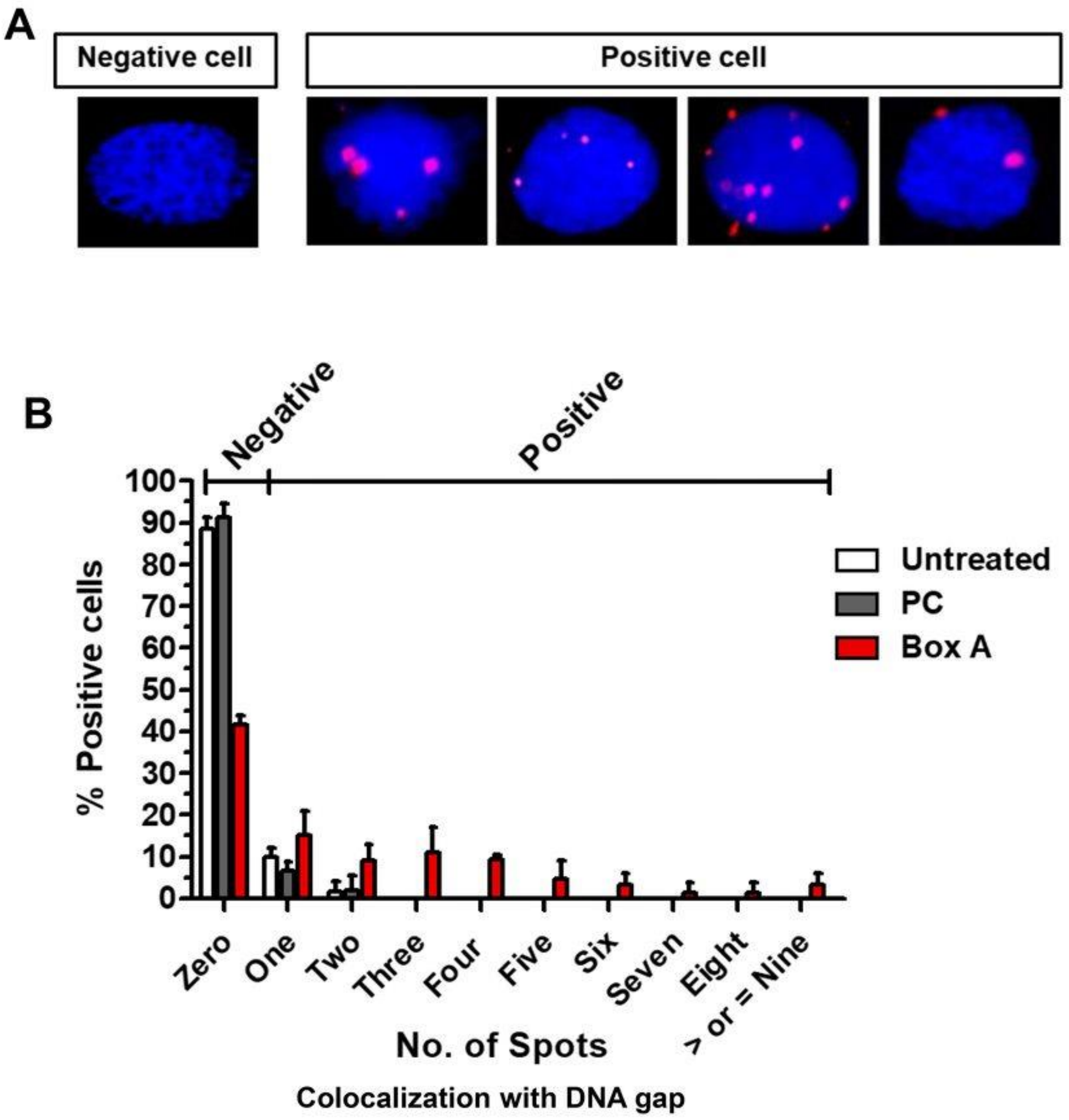

Figure S3. **Box A colocalization with the DNA gap result pattern of Fig. 2D.** We analyzed the distribution of the colocalization signals to determine Box A colocalization signal with DSB. **(A)** Representative images of positive immunofluorescence spots after DNA damage in situ ligation followed by a proximity ligation assay (DI-PLA). HK2 cells showed that Box A (red) colocalizes with DNA gaps. DNA was counterstained with Hoechst (blue). **(B)** The positive cells with different numbers of positive signals (1 to ≥ 9 spots) were enumerated and calculated as the percentage of positive cells in the spot distribution. The nuclei with more than one signal spot were counted as positive cells. The percentage of positive cells is presented as the mean ± s.e.m. and was higher in Box A-transfected cells than in PC- and untreated cells.

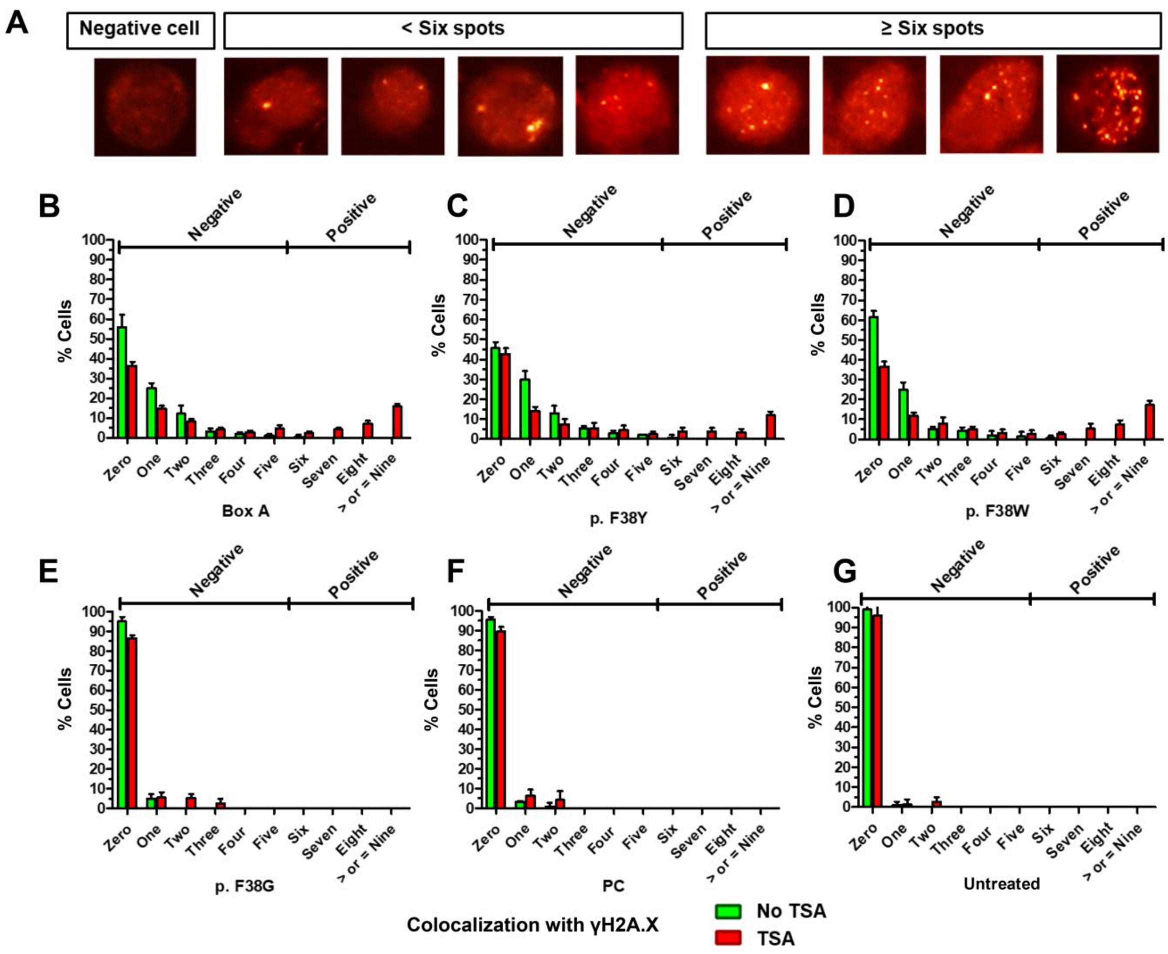

Figure S4.

The pattern of Box A colocalization signals with γH2A.X in trichostatin A (TSA)-treated cells (additional data for Fig. 2E). Box A transfected cells were treated with and without TSA and analyzed Box A colocalization signals with γH2A.X. (A) Representative immunofluorescence staining of transfected cells. Colocalization (red spot) was observed by using the combination of anti-DDDDK tag (FLAG) mouse monoclonal antibody and anti-γH2A.X rabbit monoclonal antibody. The transfected cells showed 1 to ≥ 9 positive spots and were counted and calculated as the percentage of positive cells in Box A (B), p.F38Y (C), p.F38W (D), p. F38G (E), PC (F), and untreated (G) cell lines. The percentage of positive cells was increased in the TSA treatment group compared to the untreated TSA group. Moreover, a large number of positive spots were observed in the TSA-treated group. The transfected cells that exhibited ≥ 6 spots for Box A colocalization with γH2A.X were counted as the positive cells.

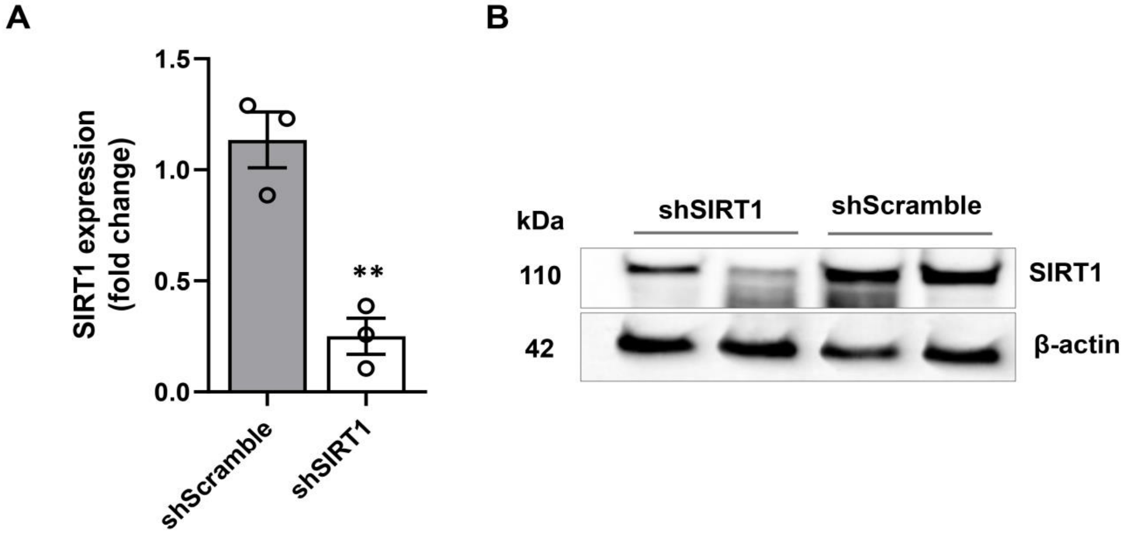

Figure S5. Expression analysis of shSIRT1 cells. To confirm the SIRT1 down regulation in shSIRT1-transfected cells, real-time PCR was performed to detect *SIRT1* expression, and Western blotting analysis showed the protein expression levels of SIRT1 in shScramble- and shSIRT1-transfected cells studied in Fig. 2F. The mean of shScramble was normalized to 1. Immunoblots were reprobed with β-actin as a control to confirm the equal loading of samples. Data represent mean ± s.e.m. **P ≤ 0.01 t-test.

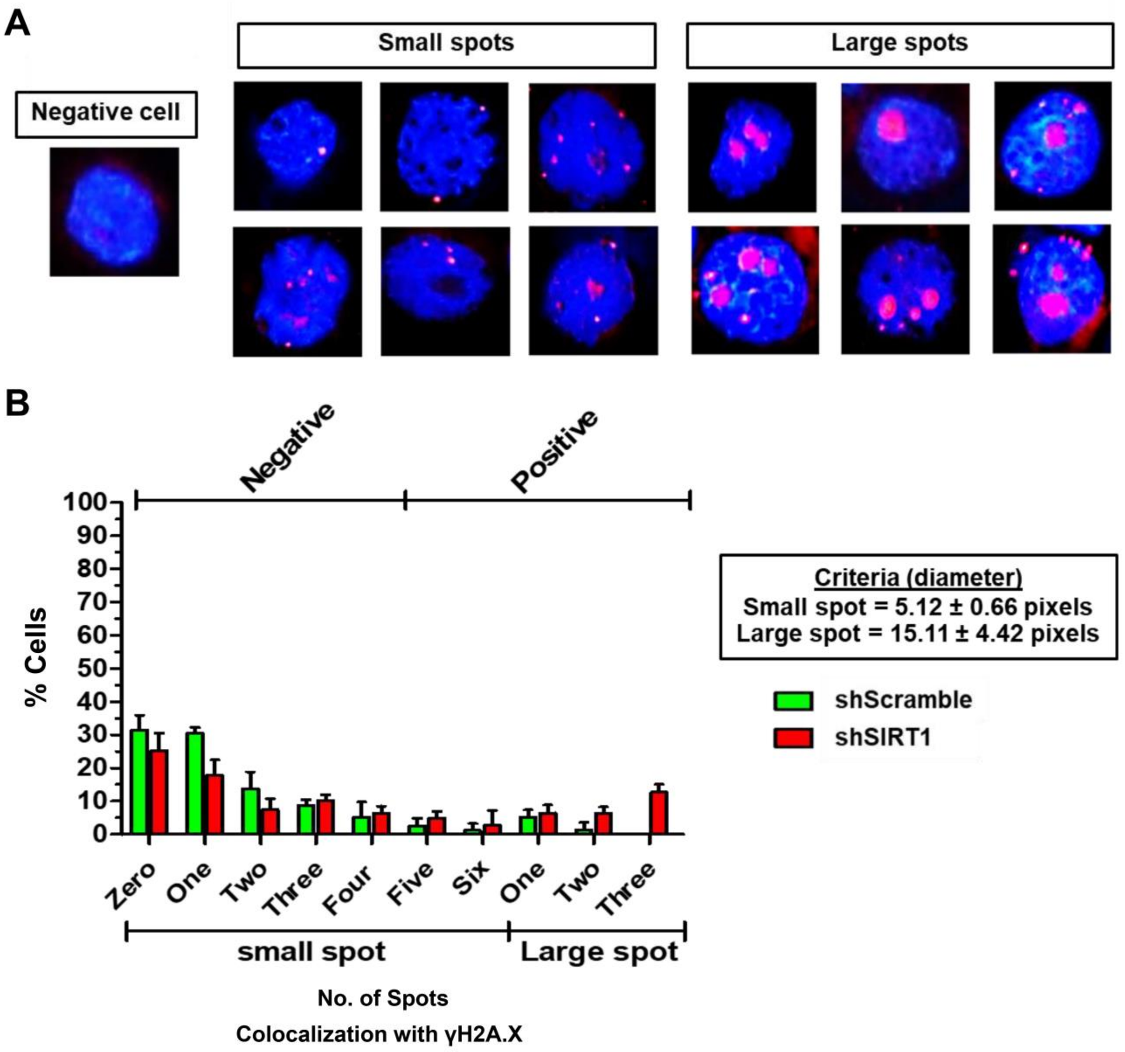

Figure S6.

The pattern of colocalization signals of Box A and γH2A.X in SIRT1 knockdown cells (additional data for Fig. 2F). SIRT1 knockdown cells and control were analyzed Box A colocalization signals with γH2A.X. (A) Colocalization of Box A and γH2A.X (pink spot) in Box A transfected cells were observed using the combination of anti-DDDDK tag (FLAG) mouse monoclonal antibody and anti-γH2A.X rabbit monoclonal antibody. The diameter of the positive spot was applied as a criterion for small (5.12 ± 0.66 pixels) and large (15.11 ± 4.42 pixels) positive signals. (B) The transfected cells with positive signals were counted and classified as the percentage of positive cells. Nuclei with ≥ 5 small spots or ≥ one large spot were identified as belonging to positive cells for the colocalization of Box A and γH2A.X. The percentage of positive cells is presented as the mean ± s.e.m. The shSIRT1 group showed that most of the transfected cells exhibited higher numbers of positive spots and larger spot sizes than the shScramble group.

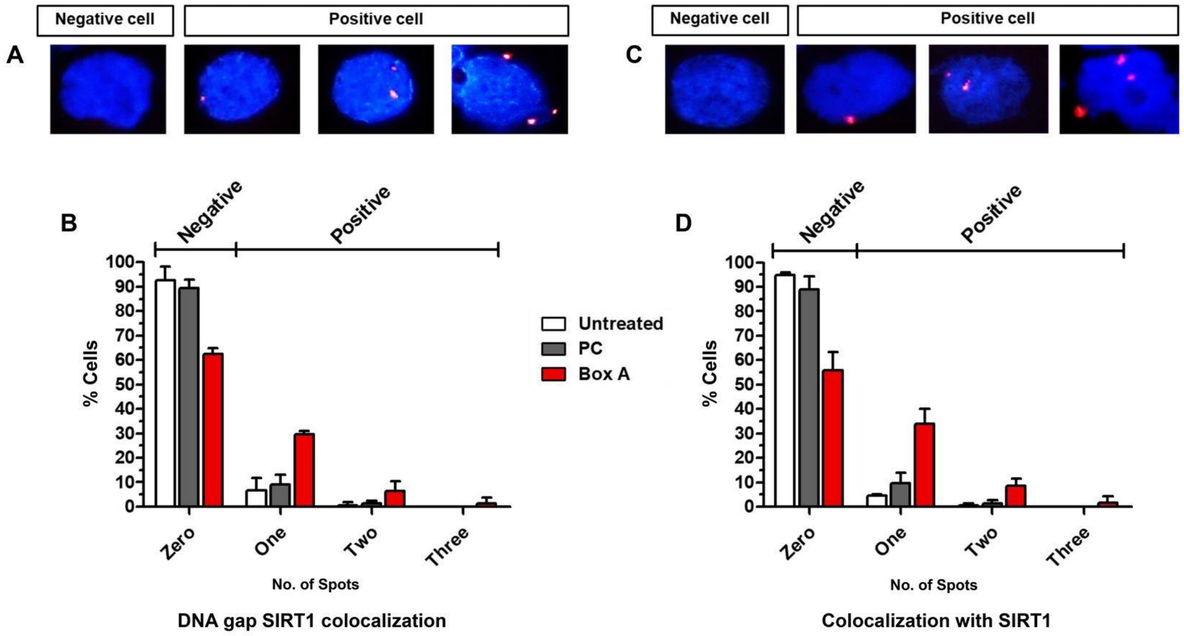

Figure S7. The pattern of colocalization signals of on SIRT1 and DNA gap and SIRT1 and Box A (additional data for Fig. 2G and 2H). Box A transfected cells were analyzed for the colocalization between DNA gap or Box A and SIRT1. **(A)** Colocalization of DNA gap and SIRT1 (red dots) in the positive cells was observed using an anti-biotin rabbit polyclonal antibody combined with an anti-SIRT1 mouse monoclonal antibody. **(B)** The numbers of positive cells with 1, 2, and 3 spots of DNA gap and SIRT1 colocalization were calculated as the percentage of positive cells. **(C)** Colocalization of Box A and SIRT1 (red dots) in the nuclei was observed using the combined reaction of anti-SIRT1 mouse monoclonal antibody and anti-DDDDK tag (FLAG) rabbit polyclonal antibody. **(D)** The number of cells with 1, 2 and 3 spots of Box A and SIRT1 colocalization were calculated as the percentage of cells. The percentage of cells is presented as the mean ± s.e.m. revealed increasing levels of DNA gap-SIRT1 and Box A-SIRT1 colocalization in Box A transfected cells compared to the PC and untreated groups.

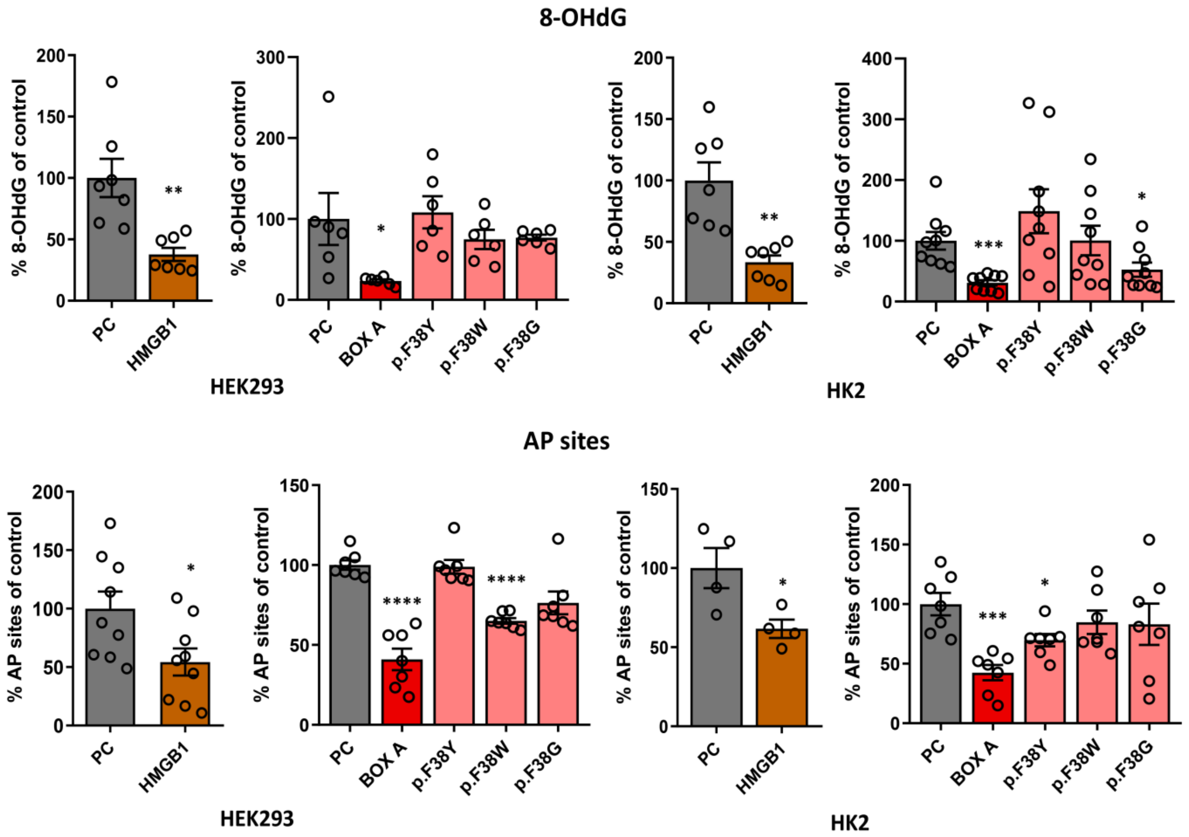

Figure S8. Reduction of endogenous DNA damage. Extension results of Fig. 4A and 4B with Box A mutants and HMGB1. To compare DNA protection of Box A with HMGB1 and other Box A mutants, we measured the level of endogenous DNA damage, 8-OHdG (%8-OHdG of control), and AP sites (%AP sites of control) of cells transfected with Box A, Box A mutants, and HMGB1 expression plasmids in comparison with PC; (n = 6-to-9 independent biological samples). Data represent mean ± s.e.m. *$P \leq 0.05$, **$P \leq 0.01$, ***$P \leq 0.001$, ****$P \leq 0.0001$; t-test.

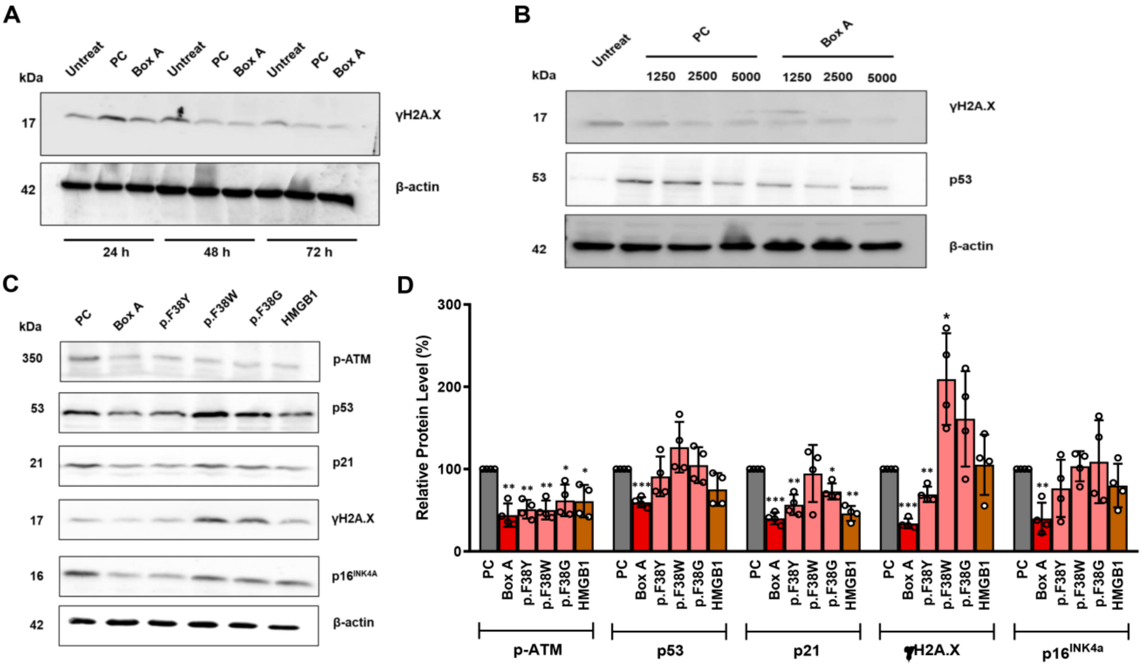

Figure S9. Reduction of DDR. Extended results from Fig. 4C with Box A mutants and HMGB1. To compare the DNA protection of Box A with HMGB1 and other Box A mutants, we measured endogenous DDR. (**A**) To evaluate the DDR, Box A and PC plasmid transfections were optimized by using various time points: 24, 48, and 72 hours to assess the protein expression levels of γH2A.X compared to those in the untreated control by western blotting analysis. (**B**) Box A and PC plasmids were optimized using 1250, 2500, and 5000 ng per test for 48 hours (the optimal time conditions for protein expression levels) to detect γH2A.X protein levels by western blots compared to the untreated control. Immunoblots were reprobed with β-actin to confirm the equal loading of samples. (**C**) To further evaluate the protein expression levels of the DDR signaling pathway, western blotting analysis was used to compare the results of Fig. 3c with Box A mutants and HMGB1. Cells were transfected with Box A, Box A mutants, and HMGB1 expression plasmids and compared to PC cells using 2500 ng per test for 48 hours. Immunoblots were reprobed with β-actin to confirm the equal loading of samples (n=3 independent biological samples). (**D**) The relative protein level percentages were determined for p-ATM (Ser1981), p53, p21, γH2A.X, and p16^{INK4A}. Data represent mean ± s.e.m. *P ≤ 0.05, **P ≤ 0.01, ***P ≤ 0.001 t-test.

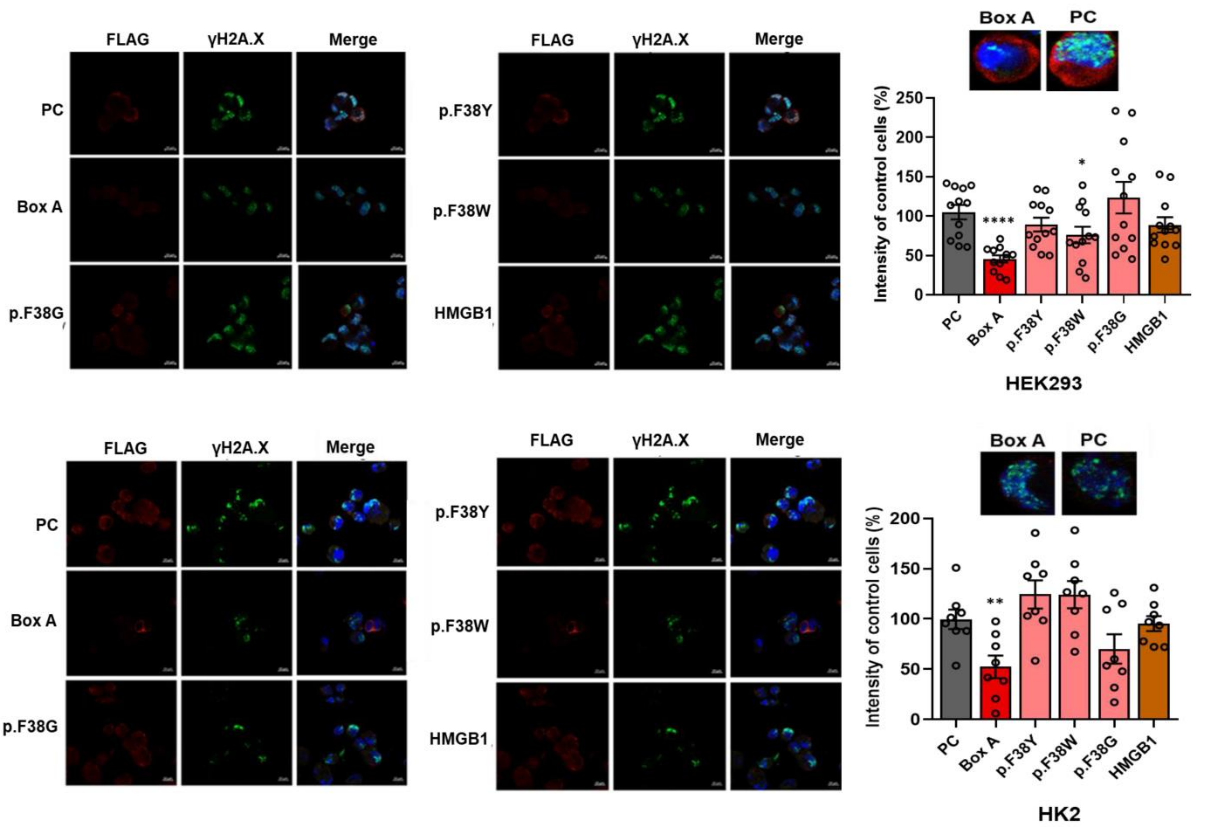

Figure S10. Reduction of γH2A.X foci. Extended results from Fig. 4D with Box A mutants and HMGB1. To compare the DNA protection of Box A with HMGB1 and other Box A mutants, we measured γ-H2A.X foci after radiation. HEK293 and HK2 cell lines were transfected with PC, Box A, Box A mutants, and HMGB1 expression plasmids for 2 days and then subjected to X-ray or gamma irradiation at a dose rate of 2 Gy/min. Representative immunofluorescence staining is shown for Flag (red), γ-H2A.X (green) and merge combined signal. DNA is counterstained with Hoechst 33342 (blue). HEK293 n=12. HK-2 n=8. All experimental data were independent biological samples: scale bar, 10 µm. Foci brightness was measured using FociCounter software. The foci intensity of each plasmid was compared to that of PC. Data represent mean ± s.e.m. *P ≤ 0.05, **P ≤ 0.01, ****P ≤ 0.0001 t-test.

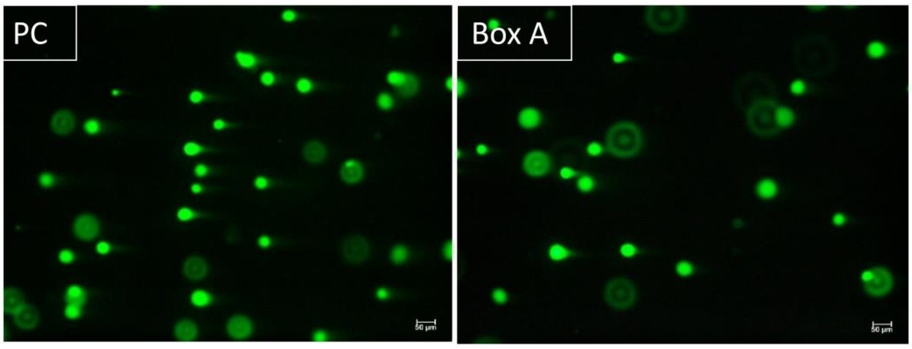

Figure S11. Detection of DNA damage by Comet assay after exposure to x-ray. Representative image of comet assay in cells overexpression with Box A and PC. Cells were transfected with indicated plasmids for 24 h and then exposed to X-ray irradiation at a dose rate of 2 Gy/min using a 6 MV Clinac®iX system linear accelerator (Varian Medical Systems, CA, USA). Comet images of the transfected cells stained with SYBR green and visualized under fluorescent microscope; Box A- transfected cells (right panel) and PC-transfected cells (left panel). Scale bar= 50 µm.

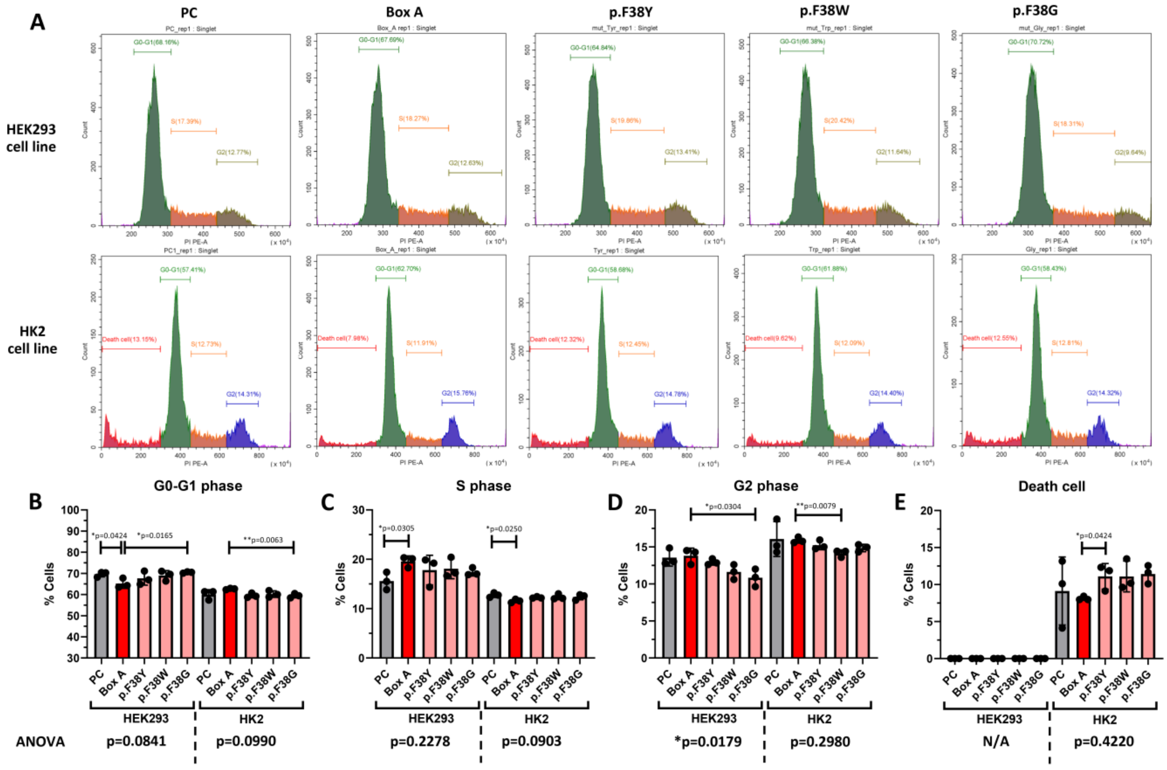

Figure S12. The percentages of transfected HEK293 and HK2 cell population in the G0-G1, S and G2 phases of the cell cycle. The transfected HEK293 (n=3) and HK2 (n=3) cells were stained with propidium iodine (PI) to observe amount of DNA in nucleus and evaluate the phase of cell cycle by flow cytometry. (A) Representative flow cytometry histograms of cell cycle analysis of HEK293 and HK2 that transfected with PC, Box A, p.F38Y, p.F38W and p.F38G. The percentages of transfected cell population in the different phase of cell cycle were calculated and represented in (B) G0-G1, (C) S, (D) G2 and (E) death cell. All experimental data were independent biological samples. Data are mean ± s.e.m. *P ≤ 0.05, **P ≤ 0.01, ***P ≤ 0.001 from one-way ANOVA.

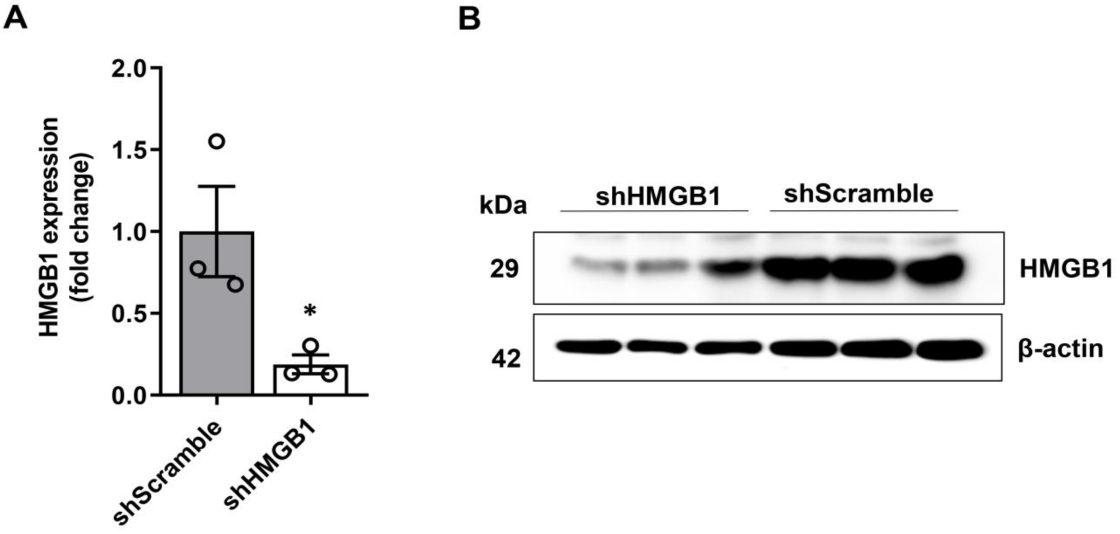

Figure S13. Expression analysis of shHMGB1 cells in Fig. 5. To confirm the HMGB1 down regulation in shHMGB1-transfected cells. HEK293 cells were transduced with the shHMGB1 lentiviral particles for 72 hours to knockdown *HMGB1* gene expression and the protein expression levels of HMGB1 in shScramble- and shHMGB1-transfected cells were measured. Immunoblots were reprobed with β-actin as a control to confirm the equal loading of samples. Data represent mean ± s.e.m. *P ≤ 0.05 t-test.

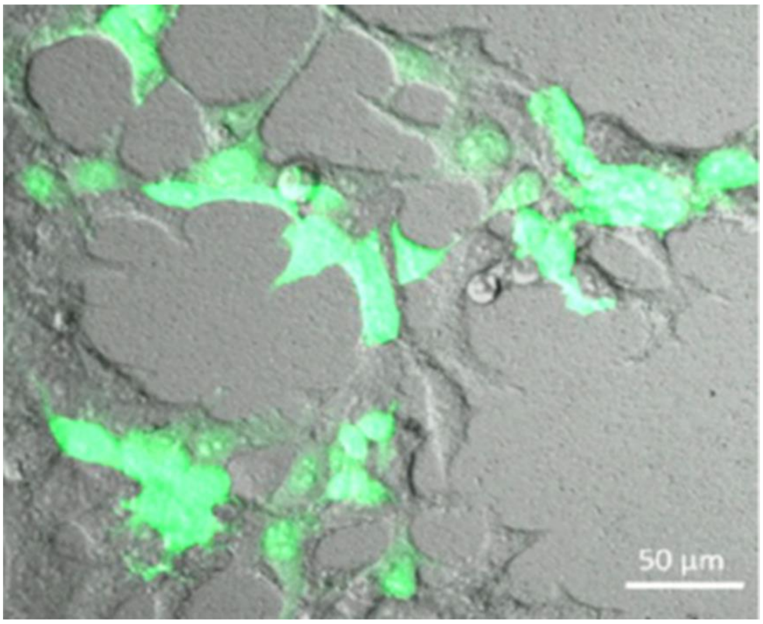

Figure S14. Box A with GFP-expressing HEK293 cells 48 hours after Ca-P nanoparticle transfection. To validate Ca-P nanoparticle transfection efficiency, a total of 5×10^4 cells were transfected with nanoparticle-coated Box A-GFP plasmid (5 µg plasmid/100 µl Ca-P nanoparticle solution). At 48 hours after transfection, the images were captured using a confocal microscope at 20X (Zeiss LSM 800, Carl Zeiss, USA). The green color displays the GFP produced by the transfected cells.

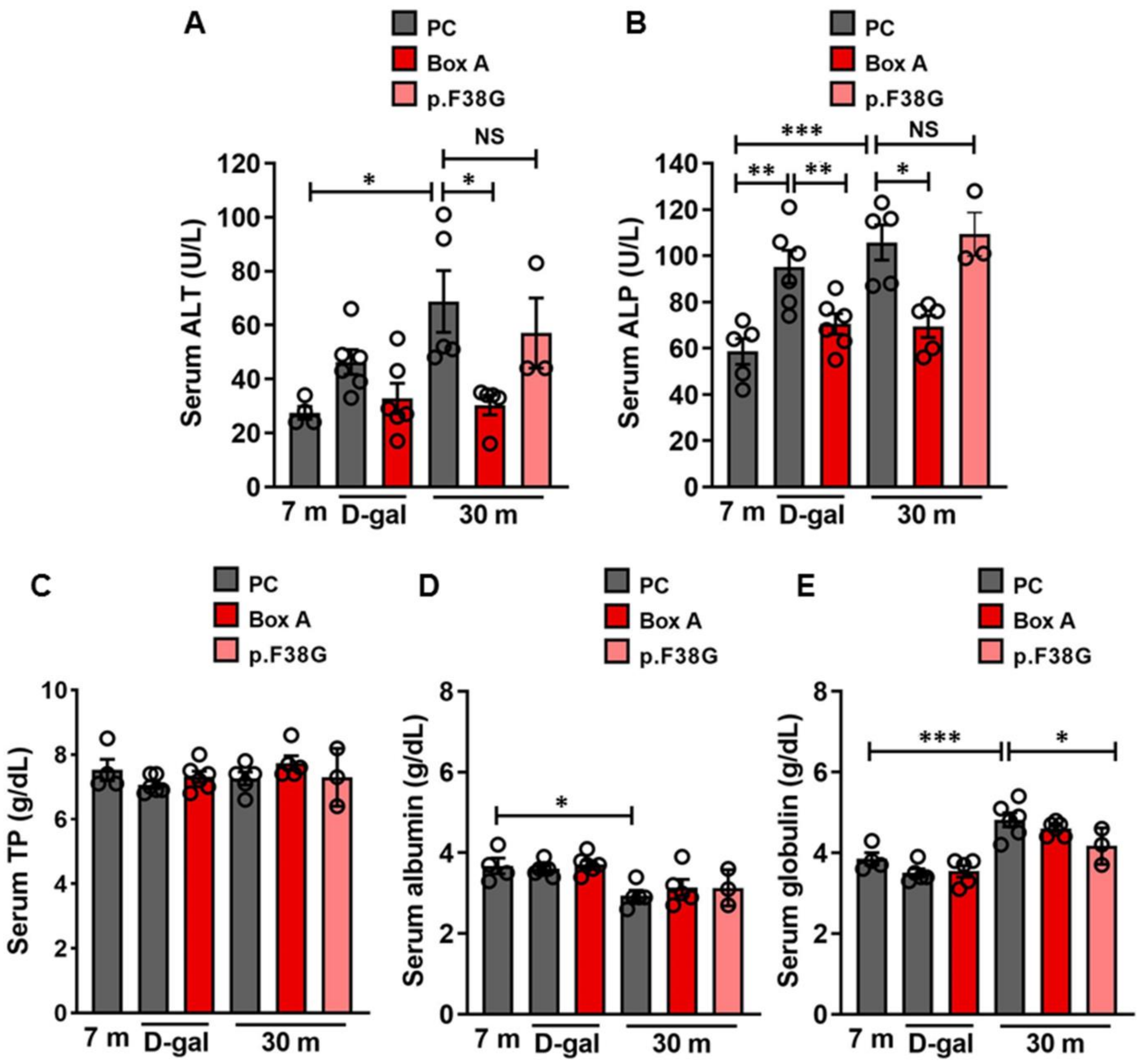

Figure S15. Improvements in serum liver function parameters by Box A treatment. The levels of (**A**) ALT, (**B**) ALP, (**C**) TP, (**D**) albumin, and (**E**) globulin in rat serum were also detected after 8 weeks of both D-gal treatment and natural aging studies (PC and Box A, n=4-6; p.F38G, n=3). Box A treatment restored liver dysfunction in both aging rat models, shown by significant decreases in serum ALT and ALP levels (A-B). Levels of TP, albumin and globulin were not significantly different among all groups (C-E). Serum globulin levels were calculated from serum TP and serum albumin levels, as mentioned in the Methods. Data represent the means ± s.e.m., $^*P \leq 0.05$, $^{**}P \leq 0.01$ and $^{***}P \leq 0.001$ one-way ANOVA followed by post hoc analysis.

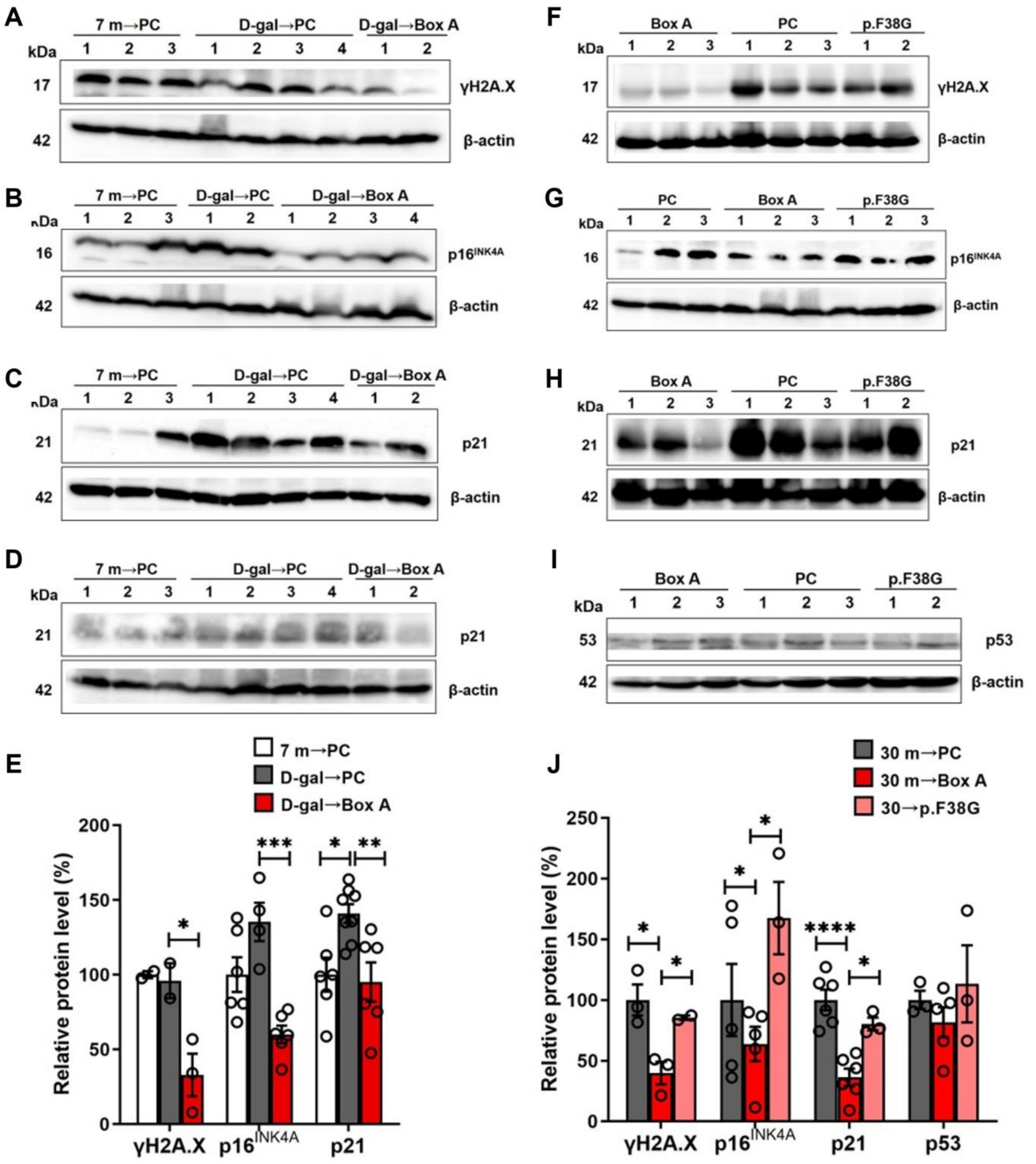

Figure S16. Aging marker protein reduction by Box A treatment. Not only γH2A.X but also the late-stage aging marker proteins $p16^{INK4A}$ and p21 were reduced in both aging rat models. (**A**, **B**, **C**) γH2A.X, $p16^{INK4A}$, and p21 protein levels in D-gal rat liver after Box A treatment compared to normal vehicle (7 m→PC) or D-gal vehicle (D-gal →PC) (n=2-6). (**E**, **F**, **G**, **H**) γH2A.X, $p16^{INK4A}$, p21, and p53 protein expression levels of 30 m rat liver after treatment, compared to age-matched vehicle or p.F38G mutant. β-Actin was reprobed and used as a loading control. The percentages of relative protein levels of D-gal (**D**) and natural (**I**) aging studies were evaluated. Each protein of PC-treated rats was normalized to 100%.

Data represent means ± s.e.m. *$P \leq 0.05$, **$P \leq 0.01$ and ***$P \leq 0.001$ one-way ANOVA followed by post hoc analysis.

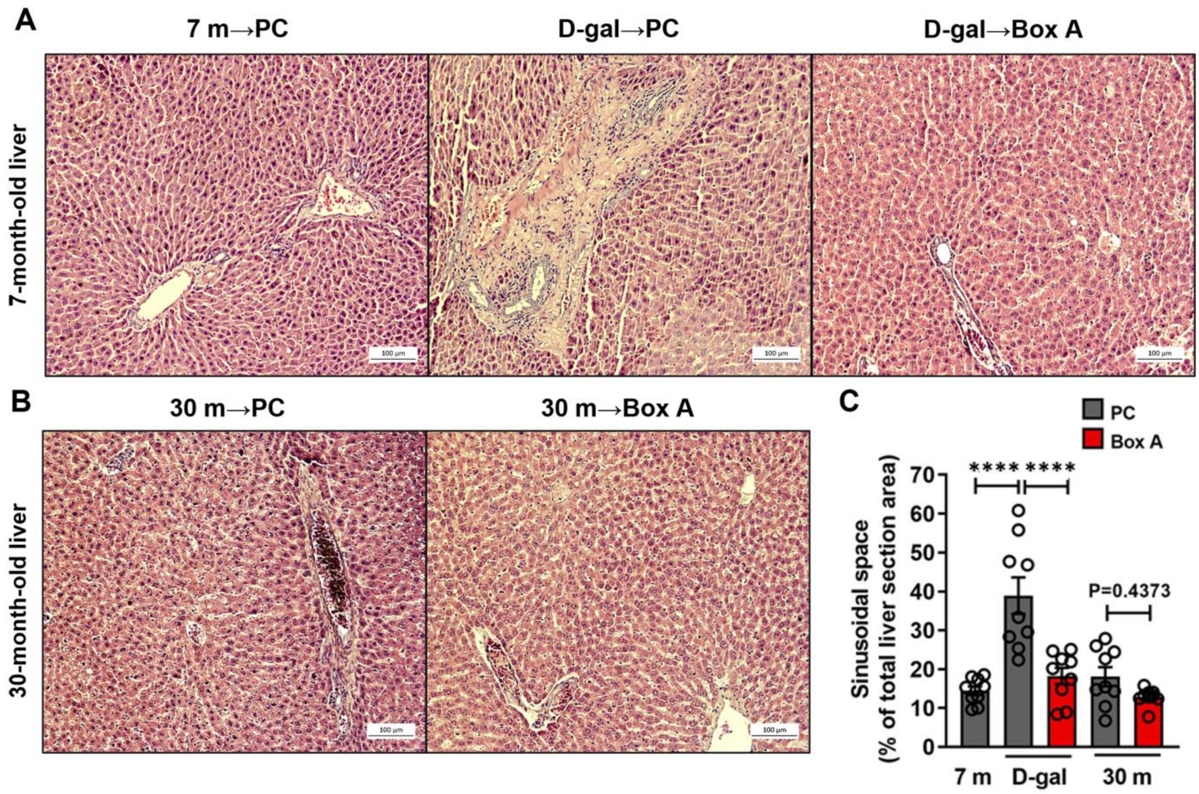

Figure S17. Reduced D-gal increased sinusoidal space under Box A treatment. Histopathological examination of rat liver tissue sections after 8 weeks of Box A treatment in D-gal and natural aging studies. (**A**) For the D-gal aging model, rat liver histopathological sections of PC-treated normal control (7 m→PC), PC-treated D-gal (D-gal→PC), and Box A-treated D-gal (D-gal→Box A) rats were examined using standard H&E staining (n=3 rats per group). (**B**) For the natural aging model, rat liver sections of PC- (30 m→PC)- or Box A- (30 m→Box A)-treated rats were also analyzed for anatomical structures after the treatment period (n=3 rats per group). A high degree of fibrosis was found in (A) D-gal→PC but not D-gal→Box A. The percentage of sinusoidal space per total area of the liver section was evaluated using ImageJ (**C**). D-gal rats displayed a significantly larger hepatic sinusoidal space than normal rats (7 m→PC). Box A-treated (D-gal→Box A) rats showed a lower percentage of the sinusoidal area than untreated D-gal rats. No significant difference in sinusoidal area among 7 m→PC, 30 m→PC, and 30 m→Box A was found. Data represent means ± s.e.m. ****$P \leq 0.0001$ one-way ANOVA followed by post hoc analysis.

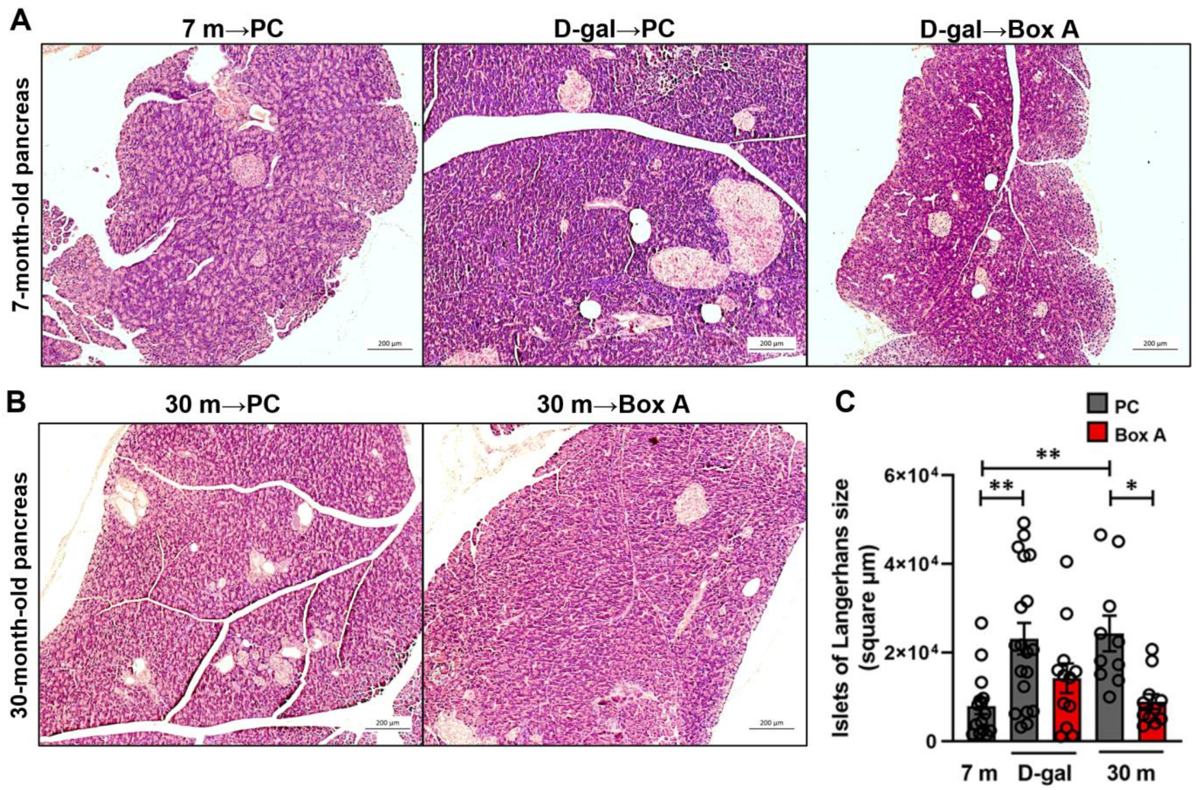

Figure S18. Islets of Langerhans sizes of natural aging rats reduced by Box A treatment. Histopathological examination of rat pancreas tissue sections after 8 weeks of Box A treatment in D-gal and natural aging studies. (**A**) For the D-gal aging model, rat pancreatic histopathological sections of PC-treated normal control (7 m→PC), PC-treated D-gal (D-gal→PC), and Box A-treated D-gal (D-gal→Box A) rats were examined using standard H&E staining (n=3 rats per group). (**B**) For the natural aging model, rat pancreatic sections of PC- (30 m→PC)- or Box A- (30 m→Box A)-treated rats were also analyzed for anatomical structures after the treatment period (n=3 rats per group). (A) The sizes of the islets of Langerhans of D-gal→PC were larger than those of both 7 m→PC and D-gal→BoxA. (B) Fat infiltration was found in the majority of the islets of Langerhans of 30 m→PC. Islets of Langerhans size were measured using ImageJ (**C**). D-gal and naturally aging rats exhibited enlarged islets of Langerhans (average size). In contrast, Box A treatment in both aging models unveiled the restoration of islets of Langerhans size compared to normal rats (7 m→PC). Data represent means ± s.e.m. $*P \leq 0.05$ and $**P \leq 0.01$ one-way ANOVA followed by post hoc analysis.

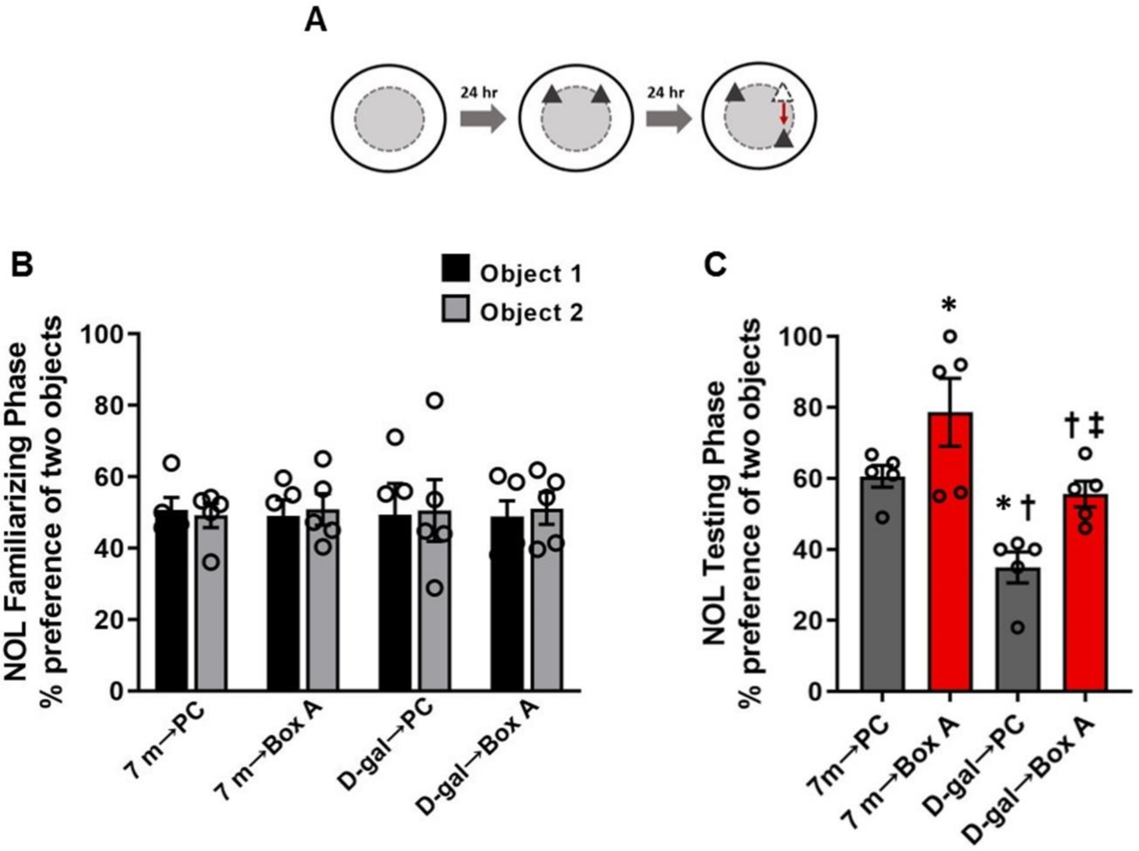

Figure S19. The effect of Box A on the hippocampal-dependent learning and memory of normal rats and D-gal-induced aging rats. (**A**) NOL protocol. Closed triangles represent the placement of the objects in the familiarizing (A, middle panel) and testing (A, right panel) phases. (**B**) Percentage of preference index in the NOL test during the familiarizing phase. (**C**) Percentage of preference index in the NOL test during the testing phase. *P < 0.05 vs. 7 m→PC, † *P*<0.05 vs. 7 m→Box A, ‡ P<0.05 vs. D-gal→PC; n=5 rats per group.

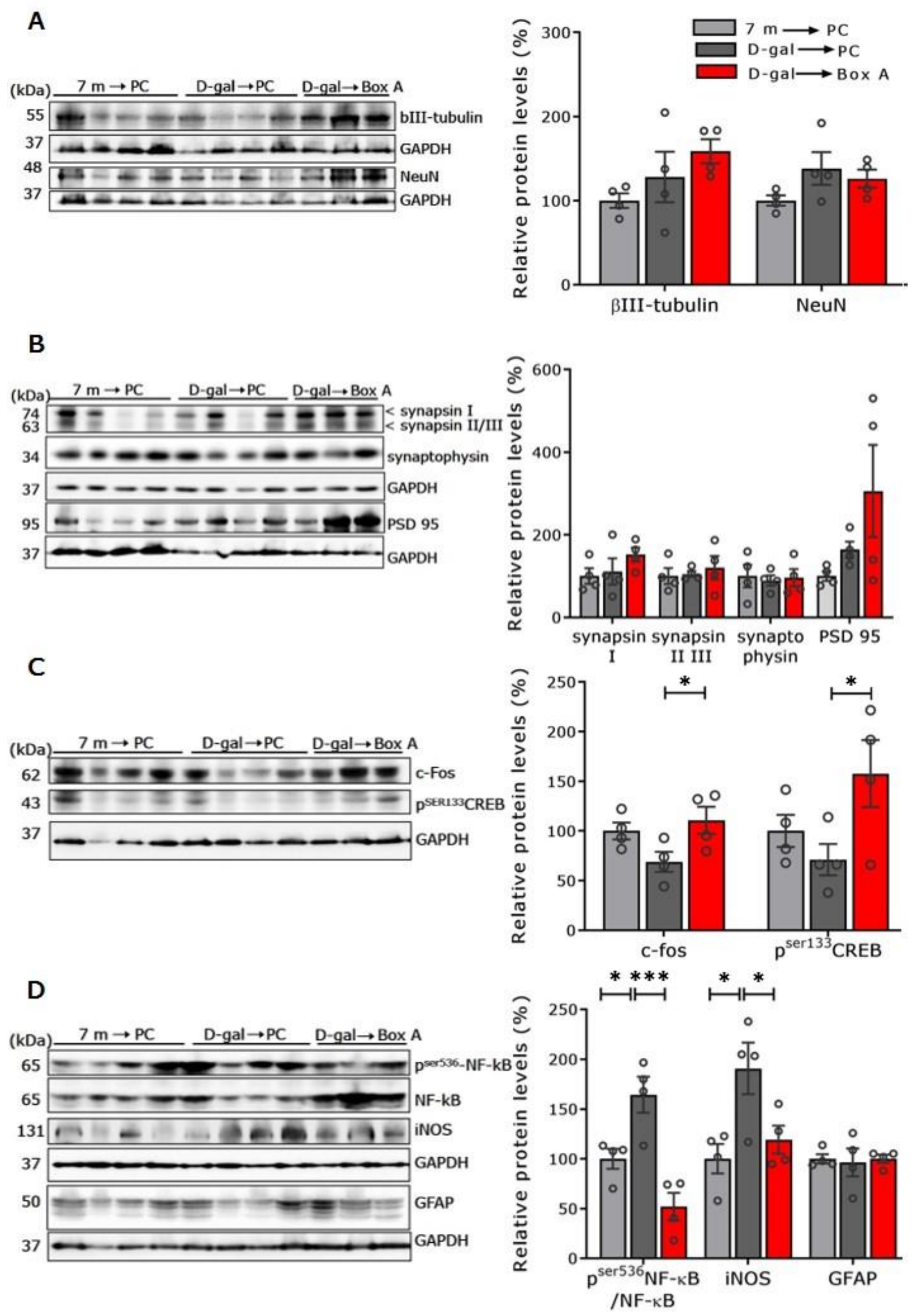

Figure S20. Box A treatment restores memory and decreases proinflammatory proteins but does not affect the number of neurons or synapses in the brains of D-gal-induced aging rats. Whole-brain lysates of D-gal-induced aging rats with or without Box A treatment compared to control rats (7 months) without D-gal injection were measured for the following protein levels using western blot: (**A**) neuron markers (BIII-tubulin and NeuN), (**B**) presynaptic markers (synapsin I II and III, and synaptophysin) and postsynaptic marker (PSD95), (**C**) proteins involved in memory function (c-fos and phosphor-CREB), and (**D**) proteins involved in inflammation (phospho-NF-κB and iNOS) and astrogliosis (GFAP). Quantification is presented as the mean optical intensity normalized to GAPDH (loading control). Data are represented as

the mean ± SEM of 4 animals/group. *P ≤ 0.05 and ***P ≤ 0.001 one-way ANOVA followed by post hoc analysis.
