# Supplementary material for: The roles of HMGB1‐produced DNA gaps in DNA protection and aging biomarker reversal
Source: FASEB Bioadv. 2022 Mar 28;4(6):408–34. doi: 10.1096/fba.2021-00131 (PMC9164245; doi:10.1096/fba.2021-00131)
Supplement: Supplementary file 2 — Table S1 [file FBA2-4-408-s002.pdf]

Table S1. Differentiation of DNA modifications, epigenetic mark vs. DNA damage

	**Epigenetic marks**			**DNA damage**			
	General	Methyl-cytosine	Youth-DNA-GAP	General	Methyl-cytosine	8-OHdG	Pathologic DSB
Source/cause	Cellular enzyme	DNMTs	HMGB1	Environ-mental hazard	Methylating agents	Oxidative stress	Radiation, conversion of SSB during DNA replication
Function/Effect	Physiologic functions	Gene control, genomic stability	DNA protection	Promote DDR, halt cell proliferation, and hasten senescence, cell death, mutation, and cancer.			
Associated proteins	Producers and maintenances	DNMTs and methylation-associated proteins	SIRT1	DNA repair proteins and DNA repair-associated proteins			
Presentation/location	Depend on cause, function, and cell types	Gene promoter or intersperse repetitive sequence & heterochromatin	Human – methylated DNA & deacetylated histone Yeast – sequence specific DNA	Depend on DNA environment, types of DNA damage, and repair. Mostly random. Generally, immediately repaired.			
DNA detection	Presentation and genomic distribution based on experimental conditions						
